# Next Generation Therapeutic Strategy for Treatment and Prevention of Alzheimer’s Disease and Aging-Associated Cognitive Decline: Transient, Once-in-a-Lifetime-Only Depletion of Intraneuronal Aβ (*i*Aβ) by Its Targeted Degradation via Augmentation of Intra-*i*Aβ-Cleaving Activities of BACE1 and/or BACE2

**DOI:** 10.3390/ijms242417586

**Published:** 2023-12-18

**Authors:** Vladimir Volloch, Sophia Rits-Volloch

**Affiliations:** 1 Department of Developmental Biology, Harvard School of Dental Medicine, Boston, MA 02115, USA; 2 Division of Molecular Medicine, Children’s Hospital, Boston, MA 02115, USA; 3 Department of Biological Chemistry and Molecular Pharmacology, Harvard Medical School, Boston, MA 02115, USA

**Keywords:** Alzheimer’s disease (AD), Aging-Associated Cognitive Decline (AACD), Amyloid Cascade Hypothesis 2.0 (ACH2.0), intraneuronal Aβ (*i*Aβ), Aβ protein precursor (AβPP), AβPP-independent *i*Aβ production pathway, ACH2.0-based drugs, BACE1/BACE2 activators

## Abstract

Although the long-standing Amyloid Cascade Hypothesis (ACH) has been largely discredited, its main attribute, the centrality of amyloid-beta (Aβ) in Alzheimer’s disease (AD), remains the cornerstone of any potential interpretation of the disease: All known AD-causing mutations, without a single exception, affect, in one way or another, Aβ. The ACH2.0, a recently introduced theory of AD, preserves this attribute but otherwise differs fundamentally from the ACH. It posits that AD is a two-stage disorder where both stages are driven by *intraneuronal* (rather than extracellular) Aβ (*i*Aβ) albeit of two distinctly different origins. The first asymptomatic stage is the decades-long accumulation of Aβ protein precursor (AβPP)-derived *i*Aβ to the critical threshold. This triggers the activation of the self-sustaining AβPP-*independent i*Aβ production pathway and the commencement of the second, symptomatic AD stage. Importantly, Aβ produced independently of AβPP is retained intraneuronally. It drives the AD pathology and perpetuates the operation of the pathway; continuous cycles of the *i*Aβ-stimulated propagation of its own AβPP-independent production constitute an engine that drives AD, the AD Engine. It appears that the dynamics of AβPP-derived *i*Aβ accumulation is the determining factor that either drives Aging-Associated Cognitive Decline (AACD) and triggers AD or confers the resistance to both. Within the ACH2.0 framework, the ACH-based drugs, designed to lower levels of extracellular Aβ, could be applicable in the prevention of AD and treatment of AACD because they reduce the rate of accumulation of AβPP-derived *i*Aβ. The present study analyzes their utility and concludes that it is severely limited. Indeed, their short-term employment is ineffective, their long-term engagement is highly problematic, their implementation at the symptomatic stages of AD is futile, and their evaluation in conventional clinical trials for the prevention of AD is impractical at best, impossible at worst, and misleading in between. In contrast, the ACH2.0-guided Next Generation Therapeutic Strategy for the treatment and prevention of both AD and AACD, namely the depletion of *i*Aβ via its transient, short-duration, targeted degradation by the novel ACH2.0-based drugs, has none of the shortcomings of the ACH-based drugs. It is potentially highly effective, easily evaluable in clinical trials, and opens up the possibility of once-in-a-lifetime-only therapeutic intervention for prevention and treatment of both conditions. It also identifies two plausible ACH2.0-based drugs: activators of physiologically occurring intra-*i*Aβ-cleaving capabilities of BACE1 and/or BACE2.

## 1. Introduction: From the ACH to the ACH2.0

### 1.1. Amyloid Cascade Hypothesis: Extracellular Aβ Causes and Drives AD

The Amyloid Cascade Hypothesis 2.0 (ACH2.0) was formulated [1,2,3,4,5] to fill in an acute need for a new, all-encompassing theory of Alzheimer’s disease following the discreditation of the initial Amyloid Cascade Hypothesis, ACH, that was introduced over three decades ago [6]. At that time, it was assumed that extracellular plaques containing amyloid-beta (Aβ) as their main component are specific for the disease. However, the immediate rationale for the introduction of the ACH was a discovery of the Aβ protein precursor (AβPP) mutation [7] affecting the production of Aβ and segregating with, and apparently causing the early onset of AD (familiar AD, FAD). The ACH postulated that increased levels of extracellular Aβ drive the disease and trigger a cascade of cellular events, including the formation of neurofibrillary tangles (NFTs, or tau-tangles), which culminates in neuronal loss. At the time of its introduction, the ACH appeared consistent with and provided a sufficient explanation for the accumulated body of empirical data; it was widely accepted and extracellular Aβ became the major target for numerous candidate AD drugs.

### 1.2. Inconsistencies of the ACH: Extracellular Aβ Can Be Ruled out as the Causative Agent of AD

A number of those drugs exhibited spectacular successes in stopping the progression of and even reversing the disease in various AD models [8,9,10]. They all, however, failed as spectacularly in human clinical symptomatic AD trials despite fulfilling their mechanistic mission, e.g., reducing substantially, up to 80%, levels of extracellular Aβ [11,12] (two apparent exceptions, clinical trials of lecanemab and donanemab, are discussed and interpreted in detail below). The complete inefficacy of the depletion of extracellular Aβ in numerous clinical trials for symptomatic AD indicated that the underlying theory, ACH, is incorrect. Moreover, as observational data accumulated, it became clear that there is no good correlation between levels of extracellular Aβ and the occurrence of AD. In a substantial fraction, about a third, of the general population, extracellular Aβ accumulates with aging to levels equal to or exceeding those seen in AD patients, yet without any cognitive impairment or the accompanying neurodegeneration [13,14,15,16,17,18,19]. The reverse is also true: cases of AD without an excessive extracellular Aβ deposition were detected [20]. Thus, the observations described above, taken cumulatively, decidedly rule out extracellular Aβ as the causative agent and driver of AD pathology.

### 1.3. Centrality and Causative Role of Aβ Are the Cornerstone of Any Theory of AD: Intraneuronal Aβ, iAβ

On the other hand, another category of accumulated data asserts undisputedly the centrality and causative role of Aβ in Alzheimer’s disease. Indeed, since 1991, when the first FAD-causing mutation was discovered [7], numerous other mutations that cause the early onset of AD were detected. All of them, without a single exception, affect either the structure or the production of Aβ. Moreover, a mutation that replaces a single residue in Aβ, the Icelandic mutation, confers upon its carriers the protection from both AD and Aging-Associated Cognitive Decline, AACD [21,22]. Together, these observations make the case for the central and causative role of Aβ in AD (and AACD) powerfully persuasive (reviewed in [1,4]). Moreover, they suggest that these two features, the centrality and the causative role of Aβ in AD, constitute the essential cornerstone of any conceivable theory of AD. At first glance, it appears that this assertion contradicts the above statement that extracellular Aβ can be ruled out as the cause of the disease. The two, however, are not inconsistent because of the occurrence of another pool of amyloid-beta, namely intraneuronal Aβ, *i*Aβ.

### 1.4. Amyloid Cascade Hypothesis 2.0: The Essence

The ACH2.0 preserves this cornerstone, i.e., the centrality and the causative role of Aβ in AD [1] (hence “ACH” in the ACH2.0) and, in addition, posits that it is also the central and causative agent in AACD [2,4]. This is the only “overlap” between the two theories of AD, but even it is rather superficial: in the ACH2.0, the Aβ in question is intraneuronal, whereas in the ACH, it is extracellular. All other features of the ACH2.0 are drastically different from those of the ACH. Thus, the ACH2.0 posits that AD is a two-stage disease (in contrast to its single-stage nature in the ACH). The first stage is asymptomatic. In it, *i*Aβ produced in the AβPP proteolytic pathway accumulates in a decades-long, even a life-long, process. If and when it reaches and crosses the critical threshold, it triggers the activation of the self-sustaining AβPP-independent *i*Aβ production pathway and the commencement of the second, devastating AD stage that includes the tau pathology and culminates in neuronal loss. The dynamics of AβPP-derived *i*Aβ accumulation appear to be the determining factor that either drives AACD and triggers AD or confers the resistance to both [4]. Thus, *all* known mutations that cause the early onset of AD augment the rate of accumulation of AβPP-derived *i*Aβ, resulting in the earlier, accelerated crossing of the second-AD-stage-activating threshold, activation of the AβPP-independent *i*Aβ production pathway, and commencement of the disease [4]. In sharp contrast, the protective Icelandic mutation reduces the rate of accumulation of AβPP-derived *i*Aβ, resulting in the delay or prevention of the threshold crossing, of the activation of the AβPP-independent *i*Aβ generation pathway, and, consequently, of the commencement of symptomatic AD [4]; it also delays or prevents the crossing of the AACD-triggering threshold and thus protects from this condition as well [2,4].

### 1.5. The AβPP-Independent Aβ Generation Pathway: The Active Core of AD

The AβPP-independent Aβ generation pathway is, therefore, the active core of AD. Its product is not secreted but is retained within the cell [1,4]; it both drives the AD pathology and propagates the operation of the AβPP-independent pathway that, in turn, produces more of it. The rationale for these two attributes of the ACH2.0, i.e., that in AD *i*Aβ is produced in the AβPP-independent pathway and that the bulk and, possibly, the entire production output is retained intraneuronally, is straightforward. In this theory of AD, *i*Aβ causes and drives the disease. Yet, the documented suppression of Aβ production by the AβPP proteolysis had no effect on the progression of AD [11,12]; hence, it is generated independently of AβPP in the disease. Likewise, a substantial depletion of the levels of extracellular Aβ had no efficacy whatsoever in clinical symptomatic AD trials; hence, Aβ generated in the AβPP-independent pathway is retained intraneuronally (reviewed in [1,4]).

## 2. The Engine That Drives AD

### 2.1. Life-Long Accumulation of AβPP-Derived iAβ at the First AD Stage: Mechanistic Aspects

Conventionally, Aβ is produced in the proteolytic/secretory pathway by two cleavages within the AβPP. The first, beta-cleavage, occurs between residues 671 and 672 and produces the C99, 99 amino acids long C-terminal fragment, which contains Aβ in its N-terminal portion. Subsequent gamma-cleavage occurs within an internal segment of C99 and generates Aβ of variable length, typically 40 (most common) or 42 residues long. The bulk of the gamma-cleavages of C99 take place on the plasma membranes, and the resulting Aβ is secreted. However, a small fraction of gamma-cleavages of C99 occurs physiologically on the intracellular membranes within various organelles, such as endoplasmic reticulum, endosomes, lysosomes, Golgi apparatus, trans-Golgi network (TGN), and mitochondria [23,24,25,26,27,28,29,30,31]. Aβ resulting from gamma-cleavages of C99 at these locations is not secreted but is retained intraneuronally and remains *i*Aβ. The physiologically occurring intraneuronal retention of a small fraction of AβPP-derived Aβ constitutes one of the two sources of *i*Aβ. The second source is the internalization of secreted Aβ [32,33,34,35,36,37]. In this case, extracellular Aβ is converted to *i*Aβ. It appears that the oligomerization of extracellular Aβ is a prerequisite for its importation inside the cell [35,36,37]. Aβ42, for example, is taken up by the cell twice as effectively as other Aβ species [33] because of its propensity to aggregate and form soluble oligomers. The importation of extracellular Aβ into the cell is facilitated by multiple cellular receptors [38,39,40,41,42,43,44,45,46]. As discussed below, when AβPP-derived *i*Aβ reaches the critical threshold, it triggers the activation of the AβPP-independent *i*Aβ production pathway, ignites the AD Engine and commences the second AD stage.

### 2.2. AβPP-Derived iAβ Accumulation Triggers AD but Is a Normal Physiological Process: At the First AD Stage the Difference between Health and Disease Is Quantitative, Not Qualitative

Numerous studies have demonstrated that *i*Aβ is the major cause of Alzheimer’s pathology [47,48,49,50,51,52,53,54,55,56,57,58,59]. It should be emphasized, however, that the mechanisms underlying the occurrence of AβPP-derived *i*Aβ, both the importation of extracellular Aβ inside the cell and the intraneuronal retention of Aβ derived from AβPP processed on the intracellular membranes, are normal physiological processes. They are capable of causing the disease, yet they occur in both healthy individuals who never develop AD and in future AD patients. Both accumulate AβPP-derived *i*Aβ but at different rates, and/or the extents of their second-AD-stage-activating thresholds are, apparently, also different. Thus, the difference between the two is not qualitative but quantitative, i.e., it is not how *i*Aβ accumulates but how fast it accumulates and how soon (if at all) it reaches the critical second-AD-stage-activating threshold, as illustrated further in the following sections below. Indeed, all known factors that facilitate the accumulation of *i*Aβ either predispose to or cause AD. For example, the cellular uptake of extracellular Aβ was shown to require ApoE [36], which occurs in several isoforms. One of those, ApoE4, was demonstrated to be much more effective in mediating the importation of extracellular Aβ than other ApoE isoforms [36,52]; it also constitutes the major risk factor for the disease. Some presenilins (PSEN) mutations cause increased production and secretion of Aβ42 [34], and thus facilitate, as discussed above, the cellular uptake of secreted Aβ. These mutations also cause the early onset of AD [34]. The Swedish AβPP mutation causes a marked increase in the fraction of AβPP processed on intracellular membranes [60] and, consequently, the augmented rate of its intraneuronal retention and so do certain PSEN mutations [61]. Both types of mutations also cause the early onset of AD [60,61]. On the other hand, the Icelandic Aβ mutation increases the rate of physiologically occurring cleavage within *i*Aβ and, consequently decreases the rate of its accumulation [21,22]; it also protects from the disease [21,22]. In an inverse example, the Flemish Aβ mutation decreases the rate of physiologically occurring cleavages within *i*Aβ [62] and thus increases the rate of its accumulation; it also causes the early onset of AD [62].

### 2.3. iAβ-Mediated Activation of the PKR and HRI Kinases Elicits the Integrated Stress Response (ISR) and Triggers Operation of the AβPP-Independent iAβ Production Pathway: Transition to the Second AD Stage

Ultimately, AβPP-derived *i*Aβ, accumulated to sufficient levels (the “critical threshold” referred to above), triggers activation of the operation of the AβPP-independent *i*Aβ production pathway and thus initiates the second AD stage. This, however, occurs indirectly, in several distinct steps. The first step is *i*Aβ-mediated activation of the PKR and HRI kinases. The connection between the occurrence and levels of *i*Aβ and the activity of PKR has been established in numerous studies in cell and animal models [63,64,65]. Physiologically importantly, activated PKR was detected in neuronal cells of AD patients [66,67]. Studies with animal models suggested the involvement of TNFα in the *i*Aβ-mediated activation of PKR [68]. Another possible mechanism involves the participation of the PKR Activator (PACT). The occurrence of the latter was indicated by the detection of co-localized PACT and activated PKR in the AD-affected human neuronal cells [69].

On the other hand, the *i*Aβ-mediated activation of the HRI kinase results from mitochondrial distress. The connection between AD and mitochondrial dysfunction is well established [70,71,72,73,74,75,76,77,78,79,80,81,82,83,84,85,86,87]; mitochondrial distress is, actually, one of the earliest pathological occurrences in the progression of AD. For mitochondrial distress to activate a cytoplasmic enzyme, a signal has to be transmitted across the mitochondrial membrane to the cytosol. This occurs via mitochondrial dysfunction-activated proteolytic cascade. First, the dysfunction activates the OMA1 mitochondrial protease, which cleaves another mitochondrial protein, DELE1. One of the two resulting fragments of DELE1 is exported to the cytosol. There, it binds to and thus activates the HRI kinase [88,89].

Both PKR and HRI are members of the eIF2α family of kinases [90] (the other two members are PERK and GNS2). When activated, either PKR or HRI, or both, phosphorylate eIF2α at its serine-51 residue thus triggering the integrated stress response, ISR. The eIF2α phosphorylation is, in fact, the “integrating” factor of the ISR: all numerous and variable cellular events and stresses that activate the ISR do so by the phosphorylation of eIF2α at the serine 51 [90,91,92,93,94,95,96,97,98,99]. The main manifestation of the ISR is a substantial reduction in global cellular protein synthesis. This occurs via the inhibition of the cap-dependent initiation of translation. At the same time, the integrated stress response propagates cap-independent translation of certain mRNAs. Notably, those include mRNA species encoding numerous transcription factors. The ACH2.0 posits that either some of these transcription factors or products of the genes regulated by them are the “missing” component(s) required for the operation of the AβPP-independent *i*Aβ production pathway [1]. Once these components are available, the pathway is activated and the second AD stage commences [1,4].

### 2.4. AβPP-Independent iAβ Production (and Retention) Pathway: Potential Underlying Mechanisms

There are several, at least four, cellular mechanisms capable of generating Aβ independently of AβPP [1,4]. Regardless of their nature (discussed below), they all share one common feature: in every conceivable AβPP-independent Aβ production pathway, translation initiates from the AUG encoding methionine 671 (Met671) of AβPP and preceding immediately, contiguously, and in-frame the C99 (and Aβ)-encoding segment of human AβPP mRNA. The occurrence of an AUG codon in such a propitious position was recognized [100] soon after human AβPP cDNA had been cloned and sequenced [101,102,103]. Moreover, not only is its location auspicious, it is situated within an optimal translation initiation nucleotide context [100], and in this respect, is unique for human AβPP mRNA where of 20 in-frame AUG codons, the AUG in question is the only one located within an optimal translation initiation nucleotide context; even the AUG encoding Met1 (the translation-initiating methionine) of human AβPP is not in an optimal translation initiation complex [100]. This circumstance suggested a possibility that the AUG encoding Met671 of human AβPP is utilized physiologically to generate C99 and Aβ independently of AβPP and that this pathway is operating inducibly in AD and, in fact, is underlying the disease.

One proposed mechanism capable of generating C99 and Aβ independently of AβPP is the internal initiation of translation of the intact AβPP mRNA commencing with the Met671 [100]. Following this proposal, several studies were carried out to test it [104,105]. They excluded it on dubious premises, as discussed elsewhere [4]. This possibility remains, in fact, viable and should be tested in a suitable AD model (see [4] on the subject of a suitable AD model). Three more potential mechanisms of AβPP-independent generation of Aβ utilize 5′-truncated AβPP mRNAs as translational templates. In every case, the AUG encoding Met671 of AβPP is the first functional translation initiation codon; in each of these three mechanisms, translation initiates conventionally (rather than via an unconventional internal initiation). One such mechanism is the internal initiation of transcription of the AβPP gene, which produces a suitable 5′-truncated AβPP mRNA. Another mechanism is a site-specific cleavage of the intact AβPP mRNA, which produces a molecule where the AUG in question is the first translation initiation codon. The third and apparently the most plausible mechanism is the asymmetric RNA-dependent amplification of human AβPP mRNA, which produces 5′-truncated AβPP mRNA with the AUG in question in the conventional translation-initiating position [1,4]. Importantly, this mechanism is not specific to AβPP mRNA but is rather widely used physiologically with a variety of mRNA species. It is defined by the activity of the RNA-dependent mRNA amplification pathway and the eligibility of mRNA species. Human AβPP mRNA is eligible and its amplification requires only the activation of the pathway; this option was, in fact, tested and proven plausible in a model experiment (rev. in [4]). It should be noted that another, arguably less probable, version of this option, yielding the same end result, is also possible, namely that the AβPP mRNA amplification pathway is constitutively operative in human neurons but the resulting C100-encoding mRNA is functionally, i.e., translationally, “silent” because it is cap-less, and its translation is enabled only under the ISR conditions.

Regardless of which of the mechanisms discussed above is employed in the AβPP-independent production of C99 and Aβ, the primary translation product in each case is not C99 but rather C100, i.e., Met-C99. This is because, when the translation-initiating methionine is followed by a residue larger than valine (in case of C100, the initialing Met is followed by aspartate, much larger than valine), it cannot, due to the geometry of the active site, be removed by the N-terminal aminopeptidases 1 and 2 (MAP1 and MAP2) that cleave-off the initiating methionine co-translationally [106,107,108,109,110,111]. When MAP1/MAP2 are inoperative, the translation-initiating methionine is eventually removed by other aminopeptidases [111] with broad specificity, but this happens, invariably, post-translationally. If C100 and, potentially, Met-Aβ were indeed produced in AD-affected neurons, the detection of either species would constitute proof of the occurrence of the process. Such proof cannot be obtained from postmortem samples because, in dying cells, C100 synthesis would cease long before the proteolysis does, and without its influx, N-terminal methionine would be completely removed from both Met-C99 and Met-Aβ. On the other hand, both C100 and Met-Aβ should be readily detectable in a suitable AD model, as described elsewhere [1,4]. The detection of Met-Aβ would be feasible because, in the ACH2.0 framework, it is retained intraneuronally [1,4]. This is presumably because Met-C99 and C99 produced independently from AβPP are processed only or mainly on the intracellular membranes. Not only this type of C99 processing was shown to occur, but also C99 gamma-cleavage on ER and TGN membranes was shown to be neuron-specific [26,27].

### 2.5. Collateral Effect: AβPP-Independent iAβ Production Pathway also Generates AβPP Intracellular Domain, AICD

Aβ is only one of the two molecules that are given rise to by the cleavage of C99. The other is AβPP Intracellular Domain, AICD. Roughly one AICD molecule is produced per molecule of Aβ generated by C99 (or C100) cleavage (in both AβPP-dependent and -independent pathways). It is generated by the epsilon cleavage of C99 (downstream from, preceding and apparently independent of the gamma-cleavage) [112], and it is not inert. AβPP intracellular domain was shown to interact with and affect a number of cellular signaling pathways and regulatory proteins [113,114,115,116,117,118,119,120,121,122,123]. It was implicated in the regulation of gene expression, in cytoskeletal dynamics, and in apoptosis [124,125]. It was demonstrated to influence the expression of neprilysin [126] and, consequently, the *i*Aβ clearance, and to impact the phosphorylation of tau protein, thus potentially contributing to the formation of neurofibrillary tangles [113,124]. It affects neuronal activity, impacts oscillations in the hippocampus, and causes deterioration of spatial memory encoding [127].

The production and accumulation of AICD produced by AβPP proteolysis occurs at the first AD stage alongside the accumulation of AβPP-derived *i*Aβ, and it is conceivable that both contribute to the transition to the second AD stage. The operation of the AβPP-independent *i*Aβ and AICD production pathway at the second AD stage markedly increases the levels of both. Therefore, there is significantly more AICD in Alzheimer’s patients (i.e., at the second AD stage) than in healthy individuals (or at the first AD stage). It is highly plausible that augmented levels of AICD contribute to AD pathology; however, the full extent of this contribution remains to be elucidated. Importantly, any drug interfering with the operation of the AβPP-independent *i*Aβ and AICD-generating pathway (see below on the subject) would stop the production of not only *i*Aβ but also of AICD generated in an AβPP-independent manner.

### 2.6. The AD Engine: Continuous Cycles of the iAβ-Stimulated Propagation of Its Own AβPP-Independent Production

To summarize the above discourse, the ACH2.0 posits that AD is a two-stage disease. At the first, asymptomatic stage AβPP-derived *i*Aβ accumulates in a decades-long process. This occurs via the retention of a fraction of Aβ resulting from the processing of AβPP on intracellular membranes and through the importation into the cell of a fraction of secreted Aβ. When AβPP-derived *i*Aβ reaches and crosses the critical threshold, it triggers the activation of the AβPP-independent *i*Aβ (and AICD) production pathway. More specifically, it mediates, via the engagement of TNFα and/or PACT, activation of the PKR kinase. Alternatively or additionally, it triggers mitochondrial distress, which, in turn, activates mitochondrial protease OMA1. OMA1 cleaves another mitochondrial protein, DELE1. One of the resulting two DELE1 fragments is exported into the cytosol where it finds, binds, and thus activates the HRI kinase. Both PKR and HRI are members of the eIF2α family of kinases. Upon activation, they phosphorylate eIF2α at the specific site thus eliciting the integrated stress response. The global cellular protein synthesis is drastically reduced via inhibition of cap-dependent initiation of translation but, concurrently, translation of a subset of mRNA species is increased in a cap-independent manner. This subset includes mRNA species encoding several transcription factors, and the ACH2.0 postulates that some of these transcription factors or products of genes up-regulated by them are the components required for the operation of the AβPP-independent *i*Aβ (and AICD) production pathway and absent in unstressed neuronal cells.

When the “missing” components are supplied, the AβPP-independent *i*Aβ generation pathway becomes operational. This marks the commencement of the second stage of AD. At this stage, the difference between health and disease is patently qualitative. The bulk or the entire Aβ output of this pathway is retained intraneuronally and *i*Aβ levels rapidly increase. This has two major consequences. First, elevated *i*Aβ levels drive AD pathology, including the generation of misfolded or unfolded *i*Aβ aggregates and the formation of neurofibrillary tangles. They can do the latter by, for example, suppressing the ubiquitin-proteosome system, consequently accelerating the accumulation of tau protein and expediting its phosphorylation [128,129,130,131] (reviewed in [4]). The second major consequence is the perpetualization of the operation of the AβPP-independent *i*Aβ production pathway for the remaining lifespan of the affected neurons. Indeed, the increasing levels of *i*Aβ produced independently of AβPP and retained intraneuronally sustain the activity of eIF2α kinases; this, in turn, maintains the integrated stress response. With all required components of the AβPP-independent *i*Aβ generation pathway continuously supplied, the pathway’s operation is perpetuated. These continuous cycles of the *i*Aβ-stimulated propagation of its own AβPP-independent production constitute the engine driving AD, the AD Engine.

**Figure 1:**
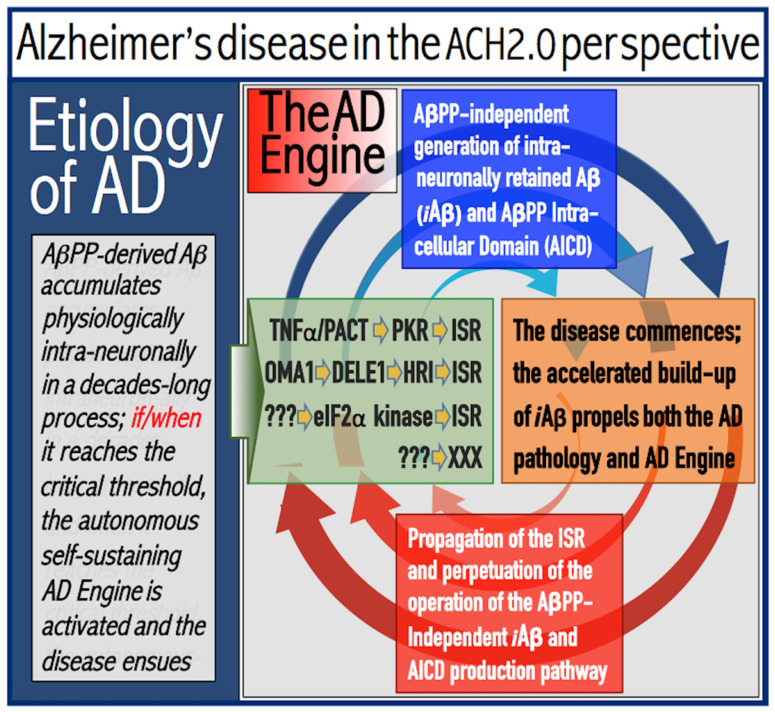
**Etiology of AD in the ACH2.0 perspective: Continuous cycles of the *i*Aβ-stimulated propagation of its own AβPP-independent production constitute the engine that drives AD.** *i*Aβ: Intraneuronal Aβ. *eIF2α*: Eukaryotic translation initiation factor 2 alpha. *PKR*, *HRI*: eIF2α kinases. *TNFα*—tumor necrosis factor alpha, *PACT*-PKR activator; both mediators of the activation of PKR. *OMA1*, *DELE1*: Mitochondrial proteins mediating the activation of HRI. *ISR*: Integrated stress response. *AICD*: AβPP intracellular domain. “*???*”: Yet-unidentified mediator(s). Note that it can act independently of AβPP-derived *i*Aβ, as apparently occurs in the case of chronic neuronal inflammation. “*XXX*”: Yet-unknown pathway activating AβPP-independent production of *i*Aβ. *Left (gray) box*: Decades-long accumulation of AβPP-derived *i*Aβ. *Middle (green) box*: Sufficient levels of *i*Aβ mediate the phosphorylation of eIF2α and the elicitation of the ISR; some proteins synthesized under the ISR conditions are the “missing” components required for the operation of the AβPP-independent *i*Aβ production pathway; alternatively, the ISR enables translation of constitutively expressed, otherwise “silent”, Met-C99-encoding cap-less mRNA. *Top (blue) box*: The AβPP-independent *i*Aβ production pathway is activated; its end products, *i*Aβ and AICD are both retained intraneuronally. *Right (pink) box*: Following the activation of the AβPP-independent *i*Aβ production pathway, steady-state levels of *i*Aβ rapidly increase. *Bottom (red) box*: The increased levels of *i*Aβ propagate the ISR and perpetuate the operation of the AβPP-independent pathway of its further production; they also drive the AD pathology. *Large Grey box*: The continuous cycles of the *i*Aβ-stimulated propagation of its own AβPP-independent generation constitute an engine that drives AD, the AD Engine; when ignited, it is autonomous, self-sustaining, and completely independent of AβPP-derived *i*Aβ.

The activation and operation of the AD Engine are illustrated in Figure 1. The left box of Figure 1 depicts a decades-long accumulation of *i*Aβ produced in the AβPP proteolytic/secretory pathway and constituting the first AD stage. As shown in the middle (green) box, if and when AβPP-derived *i*Aβ levels reach and cross a critical threshold, it mediates the activation of PKR (via TNFα and/or PACT) and HRI (by triggering mitochondrial distress, which, in turn, activates the OMA1-DELE1-HRI signaling cascade). Activated kinases phosphorylate eIF2α, thus eliciting the integrated stress response. Among new proteins produced under the ISR conditions, there are component(s) required for the operation of the AβPP-independent *i*Aβ (and AICD) production pathway. When they are supplied, the pathway is activated (upper blue box). Aβ produced independently of AβPP is retained intraneuronally and its cellular levels rapidly increase (right pink box). Steadily increasing levels of *i*Aβ propagate the ISR and perpetuate its own production via the operation of the AβPP-independent *i*Aβ/AICD generation pathway (bottom red box). Arched blue and red arrows indicate the repeated feedback cycles and denote the operation of the AD Engine; it culminates in neuronal death. Please note that while the AD Engine is operational, it is self-sustaining (due to the influx of *i*Aβ produced independently of AβPP) and completely independent from the influx of *i*Aβ produced by the AβPP proteolysis, which at this point contributes only marginally to the cellular *i*Aβ pool.

Thus, AD is a disease that is due to and completely depends upon the operation of the AβPP-independent *i*Aβ production pathway, and, apparently, the elicitation of the ISR is sufficient to activate this pathway. In other words, the AD Engine could be ignited by means other than AβPP-derived *i*Aβ. In these terms, the observed role of the persistent neuronal inflammation in the predisposition to AD could be due to its contribution to the elicitation of the integrated stress response in neuronal cells and, consequently, to the facilitation of activation of the AβPP-independent *i*Aβ production pathway even if the levels of AβPP-derived *i*Aβ are below the critical ISR-triggering threshold (further discussed in Section 17.3).

## 3. Dynamics of AβPP-Derived *i*Aβ Accumulation in Health and Disease: Alzheimer’s Disorder and Aging-Associated Cognitive Decline and the Conditionality of the “First Stage of AD”

### 3.1. Dynamics of AβPP-Derived iAβ Accumulation in Health and Disease: Alzheimer’s Disease

**Figure 2:**
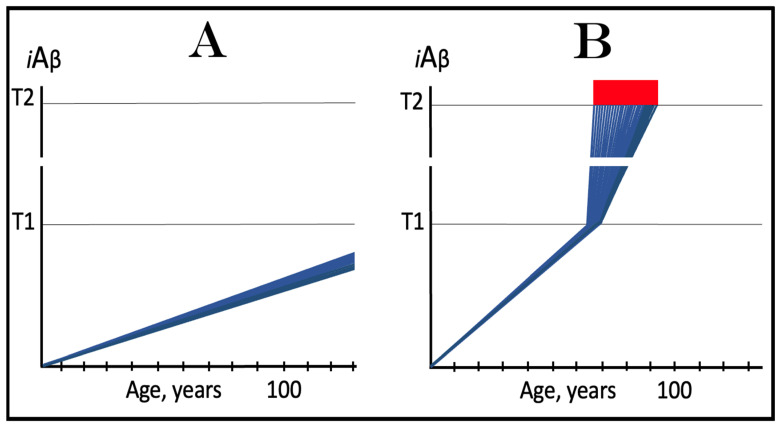
**Dynamics of AβPP-derived *i*Aβ accumulation in health and in Alzheimer’s disease.** *i*Aβ: Intraneuronal Aβ. *Blue lines*: Levels of *i*Aβ in individual neurons. ***T1*** *threshold*: The concentration of AβPP-derived *i*Aβ that mediates elicitation of the ISR and triggers the activation of the AβPP-independent *i*Aβ production pathway. ***T2*** *threshold*: The level of *i*Aβ, produced mainly in the AβPP-independent pathway, which triggers neuronal apoptosis. *Red box*: Apoptotic Zone, the range of *i*Aβ concentrations that cause the commitment to apoptosis. Panel (**A**): The levels of *i*Aβ do not cross the T1 threshold within the lifespan of a person; consequently, no disease occurs. Panel (**B**): The levels of *i*Aβ reach and cross the T1 threshold within a narrow temporal window; the AβPP-independent *i*Aβ production pathway is activated and AD commences. Eventually, the *i*Aβ levels cross the **T2** threshold. When a sufficient fraction of neurons die or lose functionality, the disease enters the end stage.

The dynamics of *i*Aβ accumulation in health and disease are depicted in Figure 2. Panel A of Figure 2 shows the dynamics of AβPP-derived *i*Aβ accumulation in individuals who do not develop AD in their lifetime. The T1 threshold, which triggers the activation of the AβPP-independent *i*Aβ production pathway, is not reached, the pathway is not activated, and there is no disease. Panel B of Figure 2 illustrates the dynamics of *i*Aβ accumulation in individuals who develop sporadic AD. The levels of *i*Aβ produced in the AβPP proteolytic pathway reach and cross the T1 threshold within a relatively narrow temporal window [1,4]. This triggers the activation of eIF2α kinases, the elicitation of the integrated stress response, and the initiation of the AβPP-independent *i*Aβ generation pathway. The activation of the latter commences the second AD stage. Levels of *i*Aβ in individual neurons increase in a broad stochastic distribution [1,4], and when they reach and cross the T2 threshold, cells commit apoptosis. When a sufficient fraction of the affected neurons lose functionality or die, AD symptoms manifest. Importantly, by the time the symptoms manifest, the bulk of the affected neurons have already crossed the T1 threshold and activated the AβPP-independent *i*Aβ production pathway [1,4]. With the continuous loss of the affected neurons, the disease eventually reaches its end state.

### 3.2. Conditionality of the “First Stage of AD”

The scenario depicted in panel A of Figure 2 is the prevailing one: the majority of the population does not develop AD. This, however, is not because these individuals are resistant to AD but because of the kinetics of their AβPP-derived *iAβ* accumulation. They simply are running out of time, i.e., the lifetime, to accumulate enough *i*Aβ and to cross the T1 threshold. Moreover, in terms of the ACH2.0, given sufficiently long lifetime, *every* individual would eventually develop AD. As discussed above, the difference between health and disease at the first AD stage is purely quantitative, AβPP-derived *i*Aβ accumulates by the very same, normal, physiological mechanisms in both cases. In individuals who eventually develop AD, either the rate of this accumulation is faster or the extent of the T1 threshold is lower. Therefore, the first AD stage cannot be defined unconditionally as a “decades-long accumulation of AβPP-derived *i*Aβ”. This is precisely what is depicted in panel A of Figure 2, yet there is no disease there and therefore no “first stage” of it. It follows that the “first stage of AD” can be such only conditionally and, moreover, only post-factum, if and when the T1 crossing occurs and the disease develops. Under any other circumstances, it is just a normal physiological occurrence. Furthermore, the “first AD stage” is mostly a term of convenience since there is no AD prior to the T1 crossing. The disease commences only with the crossing of the T1 threshold and the activation of the AβPP-independent *i*Aβ production pathway, a phase referred to in the ACH2.0 as the “second stage of AD”. Accordingly, in terms of ACH2.0, the terms “second stage of AD” and “AD” are synonymous and can be used interchangeably.

### 3.3. Dynamics of AβPP-Derived iAβ Accumulation in Health and Disease: Aging-Associated Cognitive Decline

The above considerations make it clear that the lower extent of the T1 threshold would accelerate its crossing by AβPP-derived *i*Aβ and, consequently, would expedite the commencement of the second AD stage. And indeed, statistically, in individuals predisposed to AD, the extent of the T1 threshold appears to be relatively low, lower than in the general population [132,133]. Therefore, in these individuals, as AβPP-derived *i*Aβ accumulates, no significant *i*Aβ-related damage would occur until post-T1 crossing. It is easy to visualize that with the increase in the extent of the T1 threshold, a sub-T1 level of AβPP-derived *i*Aβ would be eventually reached that causes consequential neuronal damage [4]. The ACH2.0 defines it as the T^0^ threshold: the sub-T1 level of AβPP-derived *i*Aβ, which triggers neuronal damage that manifests as Aging-Associated Cognitive Decline [2,4]. Within the framework of the ACH2.0, AACD is defined as an extended segment of the first AD stage, which occurs in individuals with a sufficiently high T1 threshold and is driven by AβPP-derived *i*Aβ. Moreover, the ACH2.0 establishes the T^0^ and T1 thresholds as the boundaries of the “AACD Zone”: the condition commences with the crossing of the former and morphs into AD when the latter is traversed [4].

**Figure 3:**
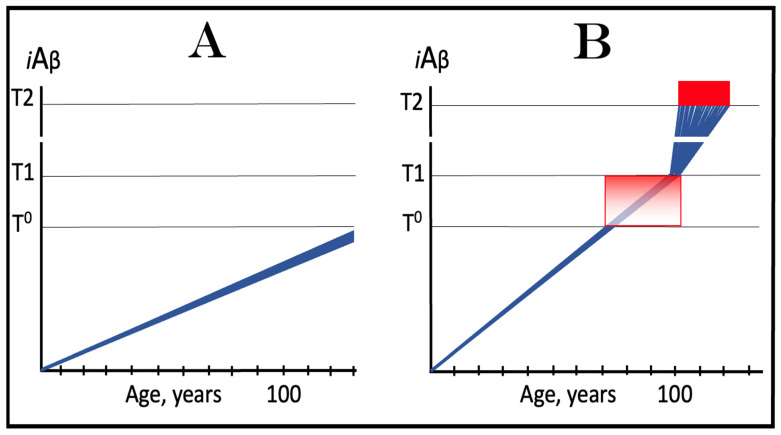
**Dynamics of AβPP-derived *i*Aβ accumulation in health and in Aging-Associated Cognitive Decline.** *i*Aβ: Intraneuronal Aβ. *Blue lines*: Levels of *i*Aβ in individual neurons. ***T*^0^** *threshold*: The concentration of *i*Aβ that triggers the neurodegeneration manifesting as AACD. ***T1*** *threshold*: The concentration of AβPP-derived *i*Aβ that mediates elicitation of the ISR and triggers the activation of the AβPP-independent *i*Aβ production pathway. ***T2*** *threshold*: The level of *i*Aβ, produced mainly in the AβPP-independent pathway, which triggers neuronal apoptosis. *Pink gradient box*: AACD Zone, the range of concentration of AβPP-derived *i*Aβ between the T^0^ and T1 boundaries. *Red box*: Apoptotic Zone, the range of *i*Aβ concentrations that cause the commitment to apoptosis. Panel (**A**): The levels of *i*Aβ do not cross the T^0^ threshold within the lifespan of a person; consequently, no AACD occurs. Panel (**B**): The levels of *i*Aβ reach and cross the T^0^ threshold; this triggers the commencement of AACD. If the T1 threshold is not crossed within the lifespan of an individual, the condition will persist for the remaining lifetime. If the T1 threshold was crossed (provided the lifespan is long enough), AACD would morph into AD. For detail, see the main text.

The dynamics of *i*Aβ accumulation in AACD versus that in health is presented diagrammatically in Figure 3. The extents of the T^0^ and T1 thresholds are identical in both panels of Figure 3. In panel A, the rate of AβPP-derived *i*Aβ accumulation is such that it does not reach the T^0^ threshold within the lifespan of an individual. Neither the T^0^ crossing nor AACD occurs. In contrast, in panel B of Figure 3, the levels of AβPP-derived *i*Aβ cross the T^0^ threshold and trigger AACD. If, subsequently, the T1 threshold were not crossed within the individual’s lifetime, the AACD would persist for the remaining lifespan, increasing in its severity in parallel with the increase in the levels of AβPP-derived *i*Aβ. If, however, the T1 threshold would be crossed within the lifetime of an individual, AACD would morph into AD (see Section 12 below on the potential overlap of AACD and AD symptoms).

## 4. ACH-Based AD Drugs: A Definition

Below, the present study analyzes the limits of the utility of the type of AD drugs that have been developed to date and are being utilized currently. It concludes that this type of drug is patently unsuitable for the treatment of AD despite its efficiency in fulfilling its mechanistic mission. The type of drug in question is comprised of ACH-based drugs, the drugs suggested by and designed on the basis of the ACH theory of AD. In the framework of the ACH, the disease is caused and driven by the extracellular Aβ. Accordingly, the rational way to treat the disease is to eliminate or reduce the cause, i.e., extracellular Aβ. Therefore, ACH-based AD drugs can be defined as agents that reduce the levels of extracellular Aβ. ACH-based AD drugs can be divided into two categories. One category comprises drugs that act outside the cell. It is exemplified by lecanemab, donanemab or other monoclonal antibodies that sequester Aβ. Any agent directly degrading or clearing or removing in any imaginable way extracellular Aβ would fall into this category. Another category of ACH-based drugs consists of agents that interfere with the intracellular production and secretion of Aβ. Since in terms of the ACH, Aβ is produced solely in the AβPP proteolytic/secretory pathway, this category includes drugs that interfere with the AβPP proteolysis. It is exemplified by verubecestat, a small-molecule drug that penetrates inside the neuron and inhibits the activity of BACE1 (Beta-Site Cleaving Enzyme). This category of drugs can be quite effective: they prevent the production and, consequently, secretion of AβPP-derived Aβ and enable effective physiological clearance of extracellular Aβ. In human clinical trials, for example, verubecestat reduced, in a dose-dependent manner, the levels of extracellular Aβ by up to 80% [11,12]. The ACH-based AD drugs fulfilled their mechanistic mission, i.e., significantly reduced the levels of extracellular Aβ, and were spectacularly successful in relieving and even reversing [8,9,10] symptoms of the disease in transgenic AD models but not in human clinical trials of symptomatic AD. The reasons for this are discussed below.

## 5. In the ACH2.0 Paradigm, Conceptually, ACH-Based Drugs Cannot Be Effective at Symptomatic Stages of AD

All tested ACH-based drugs were ineffective for symptomatic AD in human clinical trials (marginal effect of lecanemab and donanemab is discussed in Section 7 below). In light of their efficiency in animal models, their failure in human clinical trials was highly unexpected in the framework of the ACH, but this is precisely how it should be in the ACH2.0 paradigm: ACH-based drugs *cannot* be effective in symptomatic AD. The reason for this is the AβPP-independent *i*Aβ production pathway. Within the ACH2.0 framework, this process is, in fact, the cornerstone of the disease. In relation to it, AβPP-derived *i*Aβ carries out, in a way, the same function that the starter motor does in a car: when it accumulates over a critical threshold, it triggers the activation of *i*Aβ generation in an AβPP-independent manner, i.e., it ignites the AD Engine. Only then does AD commence. The production of *i*Aβ in the AβPP proteolytic pathway is simply not sufficient to drive AD pathology. On the other hand, following the activation of the AβPP-independent pathway, the production of *i*Aβ increases drastically for several reasons. First, the entire Aβ output of the pathway is retained within the neuron (in contrast to only a small fraction of the Aβ output of the AβPP proteolysis). Second, the primary translation product derived independently of AβPP (C100) comprises only 13% of 771 residues-long protein precursor of the proteolytic pathway; its production would be significantly more efficient. Third, if, as suggested elsewhere [1,4], the AβPP-independent *i*Aβ generation pathway employs the asymmetric amplification of AβPP mRNA [4], the rate of generation of *i*Aβ in this pathway would be orders of magnitude higher than the rate of production of Aβ in the AβPP proteolytic pathway. Importantly, similarly to the starter motor/engine relationship in a car, once activated, the operation of the AβPP-independent *i*Aβ production pathway is, as described above, self-sustaining and completely independent from the contribution of *i*Aβ produced by AβPP proteolysis (which becomes at this point marginal in comparison with that generated independently of AβPP). Thus, once ignited, the AβPP-independent *i*Aβ production pathway (the “AD Engine”) renders the AβPP proteolytic pathway irrelevant to the progression of AD and makes any attempt at the therapeutic utilization of the ACH-based drugs futile.

## 6. In the ACH2.0 Paradigm, Conceptually, ACH-Based Drugs Can Be Effective in Prevention of AD

Whereas, as argued in the preceding section, the production of *i*Aβ in the AβPP proteolytic pathway is irrelevant to the progression of symptomatic AD, it is, in the ACH2.0 paradigm, instrumental in triggering the disease. For this to happen, the levels of AβPP-derived *i*Aβ should reach and cross the T1 threshold (see Figure 2 above); if the T1 threshold is not crossed within the lifetime of an individual, no AβPP-independent *i*Aβ production pathway is activated and no AD occurs. This presumption lucidly suggests an effective therapeutic strategy: suppress AβPP-derived *i*Aβ accumulation and preclude its levels from reaching the T1 threshold and you will prevent the disease. And in this, the ACH-based drugs could potentially be quite effective.

As discussed above, the influx of AβPP-derived *i*Aβ occurs in two ways. One is the importation into the cell of the secreted extracellular Aβ. This is clearly a function of the levels of soluble extracellular Aβ. Reduce the levels of soluble extracellular Aβ and the rate of its cellular uptake will be reduced. The ACH-based drugs, such as monoclonal Aβ antibodies, were designed to do just this (the first category of the ACH-based AD drugs; see Section 4 above). Another mechanism of the influx of AβPP-derived *i*Aβ is the intraneuronal retention of Aβ (*i*Aβ) resulting from a fraction of AβPP processed on intracellular rather than on plasma membranes. The way to reduce the influx of retained *i*Aβ is to suppress the AβPP proteolytic pathway: less Aβ will be produced and less will be retained. Drugs, such as verubecestat (the second category of the ACH-based AD drugs; see Section 4 above), carry this out effectively [10,11,12]. ACH-based drugs of this type, actually, perform a double duty by suppressing both venues of the influx of AβPP-derived *i*Aβ. By reducing the production of AβPP-derived Aβ, they reduce its retention, whereas by lowering its secretion (less produced, less secreted), they decrease its extracellular levels and reduce the rate of its cellular uptake. By reducing the influx of AβPP-derived *i*Aβ, the ACH-based drugs would suppress the rate of its accumulation and thus delay or prevent the crossing of the T1 threshold and, consequently, the occurrence of AD.

### 6.1. Long-Term Treatment with ACH-Based Drugs: Two Possible Modes of Action

**Figure 4:**
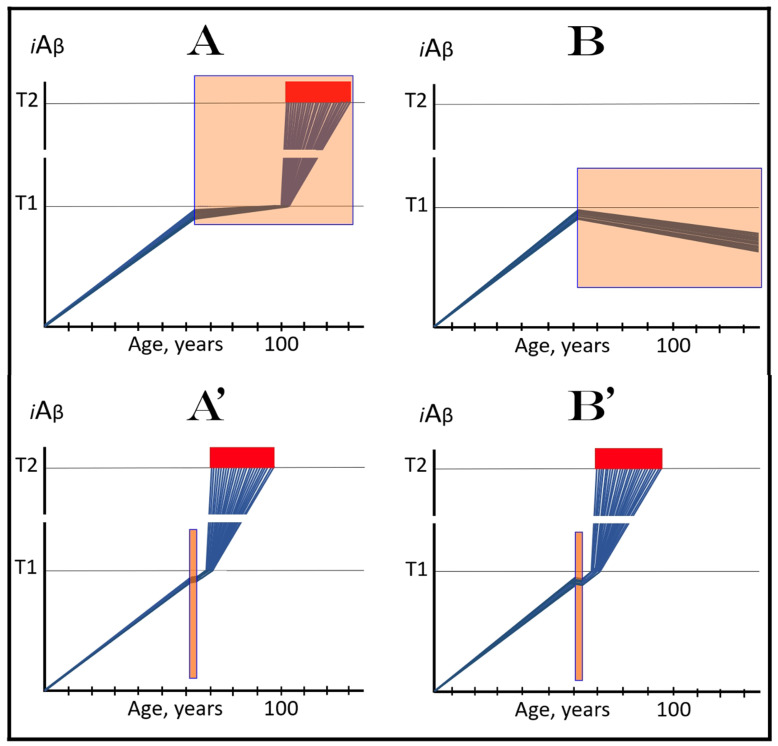
**ACH-based drugs in prevention of AD.*** i*Aβ: Intraneuronal Aβ. *Blue lines*: Levels of *i*Aβ in individual neurons. ***T1*** *threshold*: The concentration of AβPP-derived *i*Aβ that mediates elicitation of the ISR and triggers the activation of the AβPP-independent *i*Aβ production pathway. ***T2*** *threshold*: The level of *i*Aβ, produced mainly in the AβPP-independent pathway, which triggers neuronal apoptosis. *Red boxes*: Apoptotic Zone, the range of *i*Aβ concentrations that cause the commitment to apoptosis. *Orange boxes*: The duration of administration of the ACH-based drug. Panels (**A**,**B**): The administration of the ACH-based drug commences prior to the T1 crossing and continues for the remaining lifetime. Panel (**A**): The drug lowers the influx of AβPP-derived *i*Aβ and reduces the rate of its accumulation, but its levels are still increasing. Provided a sufficient lifespan, the levels of AβPP-derived *i*Aβ would eventually cross the T1 threshold and AD would ensue. Panel (**B**): The reduction in the influx of AβPP-derived *i*Aβ is sufficient to reverse its accumulation; its levels are steadily decreasing. They would not reach the T1 threshold and no AD would occur for the duration of the treatment. Panels (**A’**,**B’**): Effects of the short-term, pre-T1 crossing, administration of the ACH-based drug. Panel (**A’**): Following the cessation of the treatment, the accumulation of AβPP-derived *i*Aβ would resume at a rate equal to that exhibited prior to the treatment, the T1 threshold would be crossed and AD would ensue. In this scenario, the commencement of AD would be delayed by less than the duration of the treatment. Panel (**B’**): Upon termination of the treatment, the accumulation of AβPP-derived *i*Aβ would resume at the pre-treatment rate, the T1 threshold would be crossed, the AβPP-independent *i*Aβ production pathway would be activated, and AD would commence. In this scenario, the delay in the occurrence of the disease would be only slightly longer than the duration of the treatment.

Panels A and B of Figure 4 depict two potential modes of action of the ACH-based drugs initiated prior to the crossing of the T1 threshold and administered for a long duration. In one scenario (panel A), the influx of AβPP-derived *i*Aβ is reduced. Consequently, the rate of accumulation of AβPP-derived *i*Aβ is lowered, but its influx still outbalances the efflux and its levels continue to rise. The continuous increase in the levels of AβPP-derived *i*Aβ can result in one of two possible outcomes: (a) the T1 threshold is not crossed within the lifetime of an individual, and (b) provided the lifespan is long enough, the T1 threshold is crossed and AD develops but with a considerable delay (in comparison with the absence of the treatment).

In another scenario, illustrated in panel B of Figure 4, the drug-mediated decrease in the influx of AβPP-derived *i*Aβ is sufficient for its efflux (i.e., physiologically occurring degradation and clearance) to outbalance the influx and, consequently, its accumulation is reversed and its levels steadily decrease for the duration of the treatment. In this scenario, the T1 threshold would not be crossed within the lifetime of an individual (provided that the treatment lasts sufficiently long-term) and the disease would not occur.

### 6.2. Inefficiency of Short-Duration Treatment with ACH-Based AD Drugs

Panels A’ and B’ of Figure 4 consider the consequences of the preventive treatment with the ACH-based AD drugs administered for a short duration. Its efficiency would, obviously, depend on the degree of a decrease in the levels of AβPP-derived *i*Aβ during the treatment period. In panel A’ of Figure 4, the levels of AβPP-derived *i*Aβ do not decrease, but rather increase (albeit at a slower rate), for the duration of the treatment. Consequently, following the cessation of the treatment, the accumulation of AβPP-derived *i*Aβ would resume with a rate equal to that exhibited prior to the treatment, the T1 threshold would be crossed and AD would ensue. In this scenario, the commencement of AD would be delayed by less than the duration of the treatment (because at the end of the treatment, the levels of AβPP-derived *i*Aβ would be higher than at its beginning).

In panel B’ of Figure 4, the accumulation of AβPP-derived *i*Aβ is reversed and its levels decrease. The drug-mediated decrease in the levels of AβPP-derived *i*Aβ is, however, unlikely to be substantial. This is because of the “passive” nature of the decline: it depends on the naturally occurring clearance processes rather than on the direct action of a drug. The notion that the ACH-based drugs do not reduce or do not substantially reduce the levels of AβPP-derived *i*Aβ is consistent with the outcomes of clinical trials of lecanemab (see the following section below), where the pretreatment rate of cognitive decline resumed shortly after the cessation of the treatment. Upon termination of the short-term treatment, the accumulation of AβPP-derived *i*Aβ would resume at the pre-treatment rate, the T1 threshold would be crossed, the AβPP-independent *i*Aβ production pathway would be activated, and AD would commence. In this scenario, the delay in the occurrence of the disease (in comparison with the absence of the treatment) would be slightly longer than the duration of the treatment; more precisely, it would be equal to the duration of the treatment plus the time needed to restore AβPP-derived *i*Aβ to the pre-treatment levels at the pre-treatment rate.

## 7. Effect of Lecanemab and Donanemab in Early AD: A Dual Proof of Concepts for Inefficiency of ACH-Based Drugs in Symptomatic AD and for Their Applicability in Prevention of AD

### 7.1. Effect of Lecanemab and Donanemab in Early AD: Mechanistic Interpretation in the ACH2.0 Perspective

Two recently concluded clinical trials of the ACH-based drugs in early symptomatic AD, one of lecanemab and another of donanemab, resulted, for the first time, in a limited (very limited) success [134,135,136,137]. In both cases, the rate of cognitive decline decreased, albeit marginally, for the duration of the treatment. Both drugs are ACH-based drugs, i.e., they were designed within the ACH framework with the purpose of decreasing the levels of extracellular Aβ and thus diminishing its toxic effect. The marginal success of both drugs has been construed as evidence that the strategy worked and that the reduction in the extracellular Aβ levels indeed diminished its cytotoxicity. This, however, is inconsistent with the results of multiple preceding clinical trials where levels of extracellular Aβ were significantly reduced (to no lesser degree) by either monoclonal antibodies or by BACE inhibitors but without any efficacy whatsoever. This apparent controversy (within the ACH framework) is convincingly resolved in the ACH2.0 terms, which explain not only how the drugs worked but also why the effect was only marginal.

In both clinical trials, the drugs were administered to the patients exhibiting AD symptoms. In terms of the ACH2.0, these ACH-based drugs were not supposed to work, yet they did. Why? How? The key to understanding the “why” is the timing of the administration of the drugs. In both trials, the earliest measurable symptoms were utilized in the selection of the trials’ subjects [134,137]. In terms of ACH2.0, when symptoms manifest, the bulk of the neurons of an affected individual have already crossed the T1 threshold. However, because the T1 crossing is temporally distributed (albeit narrowly) a fraction of neurons may remain sub-T1 in early symptomatic AD, and the earlier symptoms are utilized, the more substantial this fraction is. In the lecanemab and donanemab trials, in every medicated trial subject, the drugs could not affect the over-T1 neurons but were effective in the sub-T1 neuronal fraction. As an unintended consequence of their design, both drugs, by reducing the levels of extracellular Aβ, lowered the rate of its cellular uptake, decreased the influx of *i*Aβ, and suppressed the rate of AβPP-derived *i*Aβ accumulation. This answers the “how” part: the reduction in the rate of AβPP-derived *i*Aβ accumulation delayed or prevented the crossing of the T1 threshold by and the commencement of AD pathology in the sub-T1 neuronal fraction. The effect of the drugs was marginal because they affected only a marginal fraction of neurons.

**Figure 5:**
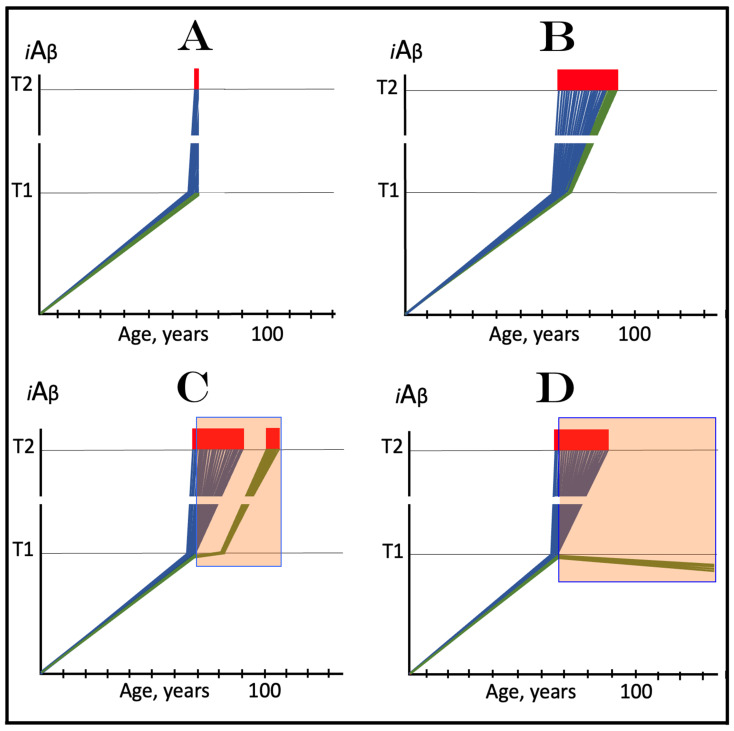
**ACH-based drugs can be only marginally effective in early symptomatic AD: lecanemab and donanemab case study.** *i*Aβ: Intraneuronal Aβ. *Blue and green lines*: Levels of *i*Aβ in individual neurons. *Green lines*: A neuronal fraction that was sub-T1 at the commencement of a drug administration. ***T1*** *threshold*: The concentration of AβPP-derived *i*Aβ that mediates elicitation of the ISR and triggers the activation of the AβPP-independent *i*Aβ production pathway. ***T2*** *threshold*: The level of *i*Aβ, produced mainly in the AβPP-independent pathway, which triggers neuronal apoptosis. *Red boxes*: Apoptotic Zone, the range of *i*Aβ concentrations that cause the commitment to apoptosis. *Orange boxes*: The duration of administration of lecanemab or donanemab. Panel (**A**): The initial state of *i*Aβ levels in individual neurons at the commencement of the treatment. Most neurons have crossed the T1 threshold but a small neuronal fraction is still sub-T1. Panel (**B**): Evolution of the initial state in the absence of a drug; AD progresses and reaches the end stage. Panels (**C**,**D**): Evolution of the initial state in the presence of lecanemab or donanemab. Importantly, the drug has no effect in the over-T1 neurons. Panel (**C**): The drug lowers the influx of AβPP-derived *i*Aβ and reduces the rate of its accumulation, but its levels are still increasing and eventually crosses the T1 threshold in the initially sub-T1 neurons; these cells will reach and cross the T2 threshold and commit apoptosis. The effect of the drug would be not only marginal but also temporary. Panel (**D**): The reduction in the influx of AβPP-derived *i*Aβ is sufficient to reverse its accumulation; its levels are steadily decreasing. They would not reach the T1 threshold for the duration of the treatment; the overall effect, however, would be only marginal. For details, see the main text.

The above reasoning is illustrated in Figure 5. Panel A of Figure 5 depicts diagrammatically the neuronal distribution immediately prior to drugs’ administration (referred to as the initial state). The bulk of neurons have crossed the T1 threshold and activated the AβPP-independent *i*Aβ production pathway, and some have reached the T2 threshold and committed apoptosis; a small fraction (depicted in green), however, remains sub-T1 at this point. Panel B illustrates the evolution of the initial state in an untreated patient. All affected neurons, including the green fraction, have crossed the T2 threshold; the disease reached its end stage. Panels C and D illustrate two potential outcomes in treated patients. The drugs have no impact on the over-T1 neuronal population; the effect is limited solely to the sub-T1 neuronal fraction. In panel C, the influx of AβPP-derived *i*Aβ is reduced, but it still accumulates, albeit at a slower rate. Eventually, it crosses the T1 threshold, activates the AβPP-independent *i*Aβ production pathway, reaches the T2 threshold, and cells commit apoptosis. This fraction was redeemed, but only temporarily. In panel D, on the other hand, the influx of AβPP-derived *i*Aβ was reduced sufficiently to reverse its accumulation. Its levels are declining and this fraction of neurons is redeemed for the duration of the treatment. It appears likely that in clinical trials either the scenario depicted in panel C played out or, if the levels of AβPP-derived *i*Aβ were actually reduced, the reduction was insignificant. This is because, upon the termination of the treatment, the pre-treatment rate of cognitive decline rapidly resumed [138].

### 7.2. Outcomes of Lecanemab and Donanemab Clinical Trials Constitute Dual Proof of Concepts: ACH-Based Drugs Are Inefficient in Symptomatic AD but Can Be Effective Preventively

It appears, therefore, that both lecanemab and donanemab worked, albeit in a very limited fashion, not because they are conceptually different from other, “failed” AD drugs but because of the early timing of their administration. In terms of the ACH2.0, it can be safely assumed that any drug lowering the extracellular Aβ levels (and, consequently, reducing its cellular uptake), including multiple Aβ-sequestering monoclonal antibodies, or inhibiting AβPP proteolysis, such as verubecestat, would have a similar effect if administered at the same early symptomatic AD stage, provided that their potential cellular toxicity would not obscure their therapeutic impact.

The outcomes of the clinical trials of lecanemab and donanemab are consistent with the notions explored in the preceding sections, namely that ACH-based drugs cannot be effective in symptomatic AD but could be efficient in the prevention of the disease. Moreover, these results, as interpreted from the ACH2.0 perspective, constitute a dual proof of concepts for these notions. In the preceding sections, when analyzing AD, we considered the “pre-symptomatic” and “symptomatic” phases of the disease. However, the analysis of the effect of lecanemab and donanemab makes it clear that such division is not entirely correct, and that in mechanistic terms, a division into pre-T1 crossing and post-T1 crossing should be always utilized when considering the effects of the ACH-based drugs. This aspect is addressed further in the following sections.

## 8. ACH-Based AD Drugs: Complications with the Long-Term Treatment for Prevention of AD

### 8.1. Long-Term Treatment with Current ACH-Based Drugs Is Inconceivable in Low-Risk Individuals

Above, in Section 6.2, we have established that the short-term preventive treatment with ACH-based drugs would have little or no effect beyond the duration of the treatment. To be effective, the treatment has to be for a long duration, potentially for the remaining lifetime of an individual, as shown in panels A and B of Figure 4 above. This, however, is entirely inconceivable for the currently available drugs. These drugs have to be delivered intravenously and frequently [134,135,136,137,138]. But this is a minor inconvenience considering their adverse effect: both lecanamab and donanemab caused life-threatening brain swelling and bleeding in treated individuals [134,135,136,137,138]. It can be firmly stated that in low-risk individuals, the possible harm from these drugs significantly outweighs their potential benefits. Conceivably, other types of drugs that reduce the influx of AβPP-derived *i*Aβ could be developed. For example, a small-molecule drug that suppresses the cellular uptake of extracellular Aβ would be no less, and possibly more, effective in the prevention of AD than lecanemab and donanemab. Inhibitors of AβPP proteolysis could be even more effective (because they would suppress both venues of the influx of AβPP-derived *i*Aβ, its retention and importation) provided their toxicity, such as was seen with verubecestat, is substantially reduced or eliminated. These proposed drugs are ACH-based. On the other hand, the present study suggests, as reflected in its title, a new, ACH2.0-based class of drugs that would be equally effective in both the prevention and treatment of AD and would render the ACH-based drugs obsolete (see below).

### 8.2. Long-Term ACH-Based Drugs Treatment for Prevention of Sporadic AD Would Be Inefficient in High-Risk Individuals

One may argue that whereas the employment of current ACH-based drugs for the prevention of AD is unacceptable in low-risk individuals, it is justified in high-risk persons. This may be correct in principle. The reality, however, is different. The reason is a profound distinction between the crossing of the T1 threshold and the manifestation of AD symptoms. The former signifies the commencement of the disease. It is closely followed by the activation of the AβPP-independent *i*Aβ production pathway. This pathway is self-sustainable and thus irreversible unless interfered with therapeutically, which is currently impossible. There is, however, a substantial gap between the times the levels of AβPP-derived *i*Aβ cross the T1 threshold and, consequently, the neurons “commit”, via the activation of the AβPP-independent *i*Aβ production pathway, to the progression of the disease, and the times AD symptoms manifest. This is the period required for sufficient accumulation of *i*Aβ produced in the AβPP-independent pathway, and it could be measured potentially in years. By definition, a high-risk individual is one who is asymptomatic but presents indications for an imminent development of AD symptoms. It could be assumed, with high probability, that in such an individual, a substantial portion, if not the majority or even the entirety, of the affected neurons have already crossed the T1 threshold. These neurons would be completely unresponsive to the ACH-based drugs. The drugs would delay or prevent the T1 crossing by a still sub-T1 neuronal fraction but its overall therapeutic effect would be insignificant.

**Figure 6:**
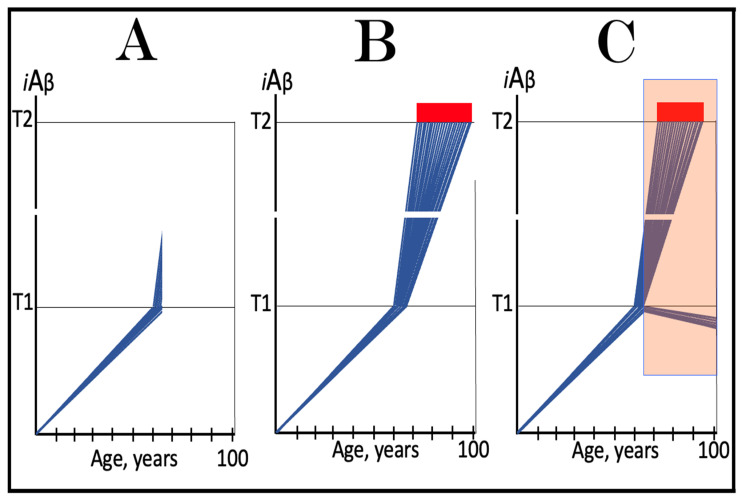
**ACH-based drugs would be inefficient for prevention of AD in high-risk individuals.** *i*Aβ: Intraneuronal Aβ. *Blue lines*: Levels of *i*Aβ in individual neurons. ***T1*** *threshold*: The concentration of AβPP-derived *i*Aβ that mediates elicitation of the ISR and triggers the activation of the AβPP-independent *i*Aβ production pathway. ***T2*** *threshold*: The level of *i*Aβ, produced mainly in the AβPP-independent pathway, which triggers neuronal apoptosis. *Red boxes*: Apoptotic Zone, the range of *i*Aβ concentrations that cause the commitment to apoptosis. *Orange box*: The duration of administration of an ACH-based drug. Panel (**A**): The initial state of the *i*Aβ levels in the neuronal population. The individual is asymptomatic, yet a significant fraction of the affected neurons have crossed the T1 threshold and activated the AβPP-independent *i*Aβ production pathway; these neurons are insensitive to the ACH-based drugs. Panel (**B**): The evolution of the initial state in the absence of the treatment. The levels of *i*Aβ cross the T1 threshold in all affected neurons. Eventually, the T2 threshold is crossed, cells commit apoptosis, and the disease reaches the end stage. Panel (**C**): The evolution of the initial stage in the presence of the ACH-based drug. The over-T1 neurons are not affected by the drug. In these cells, *i*Aβ, produced independently of AβPP, accumulates and drives the AD pathology; neurons reach and cross the T2 threshold and commit apoptosis. The sub-T1 neurons, on the other hand, are protected by the drug (to simplify discussion, the best case scenario is assumed: the drug reverses the accumulation of AβPP-derived *i*Aβ and its levels steadily decrease). This neuronal fraction is redeemed for the duration of the treatment but the overall therapeutic effect of the drug could be insignificant.

The above scenario is illustrated in Figure 6. Panel A depicts the initial state of the neuronal population of a high-risk individual. The levels of AβPP-derived *i*Aβ have crossed the T1 threshold and the AβPP-independent *i*Aβ production pathway has been activated in the majority of the affected neurons (referred to as “over-T1”), whereas a fraction of affected neurons remains sub-T1 by the time of the drug administration. Panel B of Figure 6 shows the evolution of the initial state in the absence of the treatment. The levels of AβPP-derived *i*Aβ reach and cross the T1 threshold in the initially sub-T1 neuronal fraction. The AβPP-independent *i*Aβ production pathway is activated in all affected neurons. The T2 threshold is crossed and apoptosis is triggered in all affected neurons; the disease reaches the end stage. Panel C of Figure 6 shows the evolution of the initial state of the neuronal population in a high-risk individual treated with an ACH-based drug (orange box). As discussed above, the over-T1 neurons are not affected by the drug. In these cells, *i*Aβ, produced independently of AβPP, accumulates and drives AD pathology. AD symptoms manifest, neurons reach and cross the T2 threshold, and commit apoptosis. The sub-T1 neurons, on the other hand, are protected by the drug (to simplify the discussion, the best case scenario is assumed: the drug reverses the accumulation of AβPP-derived *i*Aβ and its levels steadily decrease). This neuronal fraction is redeemed for the duration of the treatment but the overall therapeutic effect of the drug could be insignificant.

## 9. In the ACH2.0 Paradigm, ACH-Based Drugs Should Be Effective in Treatment of AACD

In terms of ACH2.0, Aging-Associated Cognitive Decline is a segment of the first stage of AD in individuals with a sufficiently high extent of the T1 threshold [2,4]. AACD commences upon the crossing of the T^0^ threshold and either continues for the remaining lifespan or morphs into AD if the T1 threshold is subsequently crossed (illustrated in Figure 3 above). Like Alzheimer’s disease, it is driven by *i*Aβ but, in contrast to AD, it is solely AβPP-derived *i*Aβ at levels ranging from the T^0^ to T1 thresholds (“AACD Zone” is defined above as sub-T1 levels of AβPP-derived *i*Aβ causing neuronal damage manifesting as AACD). It follows that the ACH-based drugs could potentially be effective in the treatment, even cure, of AACD. Indeed, if a drug is administered that lowers the rate of accumulation of AβPP-derived *i*Aβ, the rate of the progression of the condition will slow down accordingly for the duration of the treatment. Moreover, if an ACH-based drug were employed, which suppresses the influx of AβPP-derived *i*Aβ sufficiently to reverse its accumulation, its levels would be steadily declining for the duration of the treatment. Eventually, the levels of AβPP-derived *i*Aβ can cross, in a reverse direction, the T^0^ threshold. If and when this occurs, the levels of AβPP-derived *i*Aβ would be below the T^0^ threshold and the patient would be cured (technically; the degree of cure would depend on the ability of the affected neurons to restore their functionality and of cognitive functions to recover). The potential outcomes of these scenarios appear to be AACD stage-specific and are further discussed and illustrated in Section 11 below.

## 10. ACH-Based Drugs Were Spectacularly Successful in Transgenic AD Models for the Same Reason They Would Be in AACD: Mechanistic Interpretation in the ACH2.0 Perspective

In contrast to their complete inefficiency in symptomatic AD, the ACH-based drugs were spectacularly effective in transgenic AD models: they reduced, even reversed, various symptoms of the disease [8,9,10]. In terms of ACH2.0, the reason for this is the AβPP-independent *i*Aβ production pathway. In humans, it renders the Aβ production in the AβPP proteolytic pathway irrelevant to the progression of the disease and brands its targeting at symptomatic stages as futile (see above). *In mice, however, it is inoperative*. Indeed, Alzheimer’s disease appears to be human-specific, or at least species-specific; it does not occur even in long-living mammals such as elephants. As discussed elsewhere [4], four physiologically occurring mechanisms could be responsible for the operation of the AβPP-independent *i*Aβ production pathway in humans. Of those, the most plausible is the asymmetric RNA-dependent amplification of AβPP mRNA [4]. While human AβPP mRNA is eligible for this process, its mouse counterpart is not, and human AβPP mRNA expressed in transgenic models is also rendered ineligible due to its altered (during transgene construction) 5′ terminus [4].

The absence of the AβPP-independent *i*Aβ generation pathway in transgenic AD models explains why they never develop the full spectrum of AD pathology, most notably neurofibrillary tau tangles. Moreover, it can be unequivocally stated that *the current transgenic AD models can never develop AD; they are not really “AD models”*. This is in accordance with the definition of AD as a condition initiated and driven by the AβPP-independent *i*Aβ production pathway and with the presumed mechanistic inability to reach the adequate AD pathology-driving levels of *i*Aβ with the AβPP-independent pathway of its production inoperative. From this perspective, the transgenic AD models are, in fact, models of AACD, or of “enhanced” AACD. As in AACD, transgenic models accumulate AβPP-derived *i*Aβ via its intraneuronal retention and through cellular uptake of secreted Aβ. Because, in these models, Aβ is acutely overproduced from multiple AβPP transgenes (typically containing FAD and/or PSEN mutations), AβPP-derived *i*Aβ accumulates faster and to a higher extent, and could cause symptoms more severe than in typical AACD, hence “enhanced AACD”. In addition, in these models, the levels of *i*Aβ are sufficient to elicit the ISR, which, in turn, causes the impairment of learning and long-term memory formation due to suppression of protein synthesis. Therefore, it can be stated that the ACH-based drugs were spectacularly effective in the treatment of symptoms in transgenic models precisely for the same reason they should be effective in AACD, namely due to their ability to reduce or reverse the rate of AβPP-derived *i*Aβ accumulation. The current transgenic “AD” models can be useful for multiple purposes, but their utility in the investigation of AD is acutely limited. The principles of design and construction of an adequate human-neuronal-cell-based AD model are described elsewhere [1,2,4]. Eventually, with a fuller understanding of the molecular mechanism underlying the AβPP-independent *i*Aβ generation pathway, transgenic animal models can be developed where this pathway is operative, and which faithfully epitomizes the disease. In the ACH2.0 paradigm, the operative AβPP-independent *i*Aβ production pathway is the fundamental requirement for any such model.

## 11. ACH-Based Drugs in Treatment of Different Stages of AACD

The present section addresses only the effects of the long-duration employment of ACH-based drugs in the treatment of AACD. This is because the short-term deployment of these drugs is inapplicable in AACD for the same reason they are unsuitable, as discussed in Section 6.2 above, for the prevention of AD, with the effect lasting not much longer, and possibly shorter, than the duration of the treatment.

**Figure 7:**
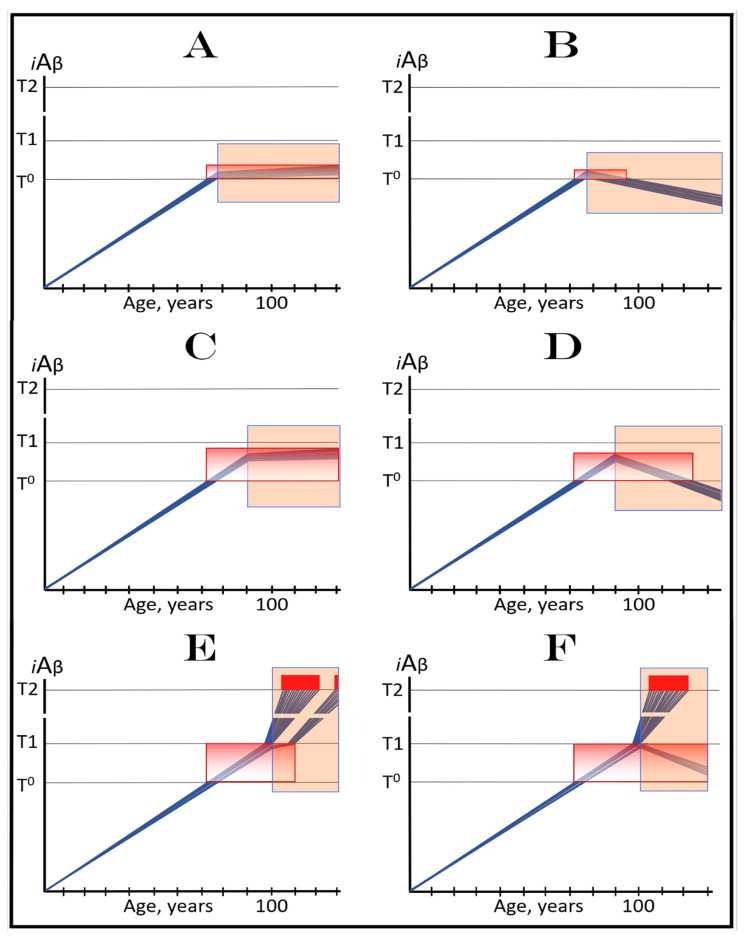
**ACH-based drugs could be effective in the treatment of AACD but not at its late stages.** *i*Aβ: Intraneuronal Aβ. *Blue lines*: Levels of *i*Aβ in individual neurons. ***T*^0^** *threshold*: The concentration of *i*Aβ that triggers the neurodegeneration manifesting as AACD. ***T1*** *threshold*: The concentration of AβPP-derived *i*Aβ that mediates elicitation of the ISR and triggers the activation of the AβPP-independent *i*Aβ production pathway. ***T2*** *threshold*: The level of *i*Aβ, produced mainly in the AβPP-independent pathway, which triggers neuronal apoptosis. *Pink gradient boxes*: AACD Zone, the range of concentration of AβPP-derived *i*Aβ between the T^0^ and T1 boundaries. *Red boxes*: Apoptotic Zone, the range of *i*Aβ concentrations that cause the commitment to apoptosis. *Orange boxes*: The duration of administration of an ACH-based drug. Panels (**A**,**B**): ACH-based drugs in early AACD. Panel (**A**): The drug reduces the rate of AβPP-derived *i*Aβ accumulation and its levels increase significantly slower than pre-treatment. Since the severity of the disease is a function of the levels of AβPP-derived *i*Aβ within the AACD Zone, the condition is substantially milder than it would have been if untreated. Panel (**B**): The drug reverses the rate of the AβPP-derived *i*Aβ accumulation. Its levels steadily decrease and eventually cross, in reverse, the T^0^ threshold. At this point the patient is technically cured and remains disease-free for the duration of the treatment. Panels (**C**,**D**): Effect of an ACH-based drug at mid-stage of AACD. Panel (**C**): The drug reduces the rate of AβPP-derived *i*Aβ accumulation. AACD progresses significantly slower and is milder than it would in the absence of a drug. Panel (**D**): The drug reverses the rate of accumulation of AβPP-derived *i*Aβ and causes steady decrease in its levels; the condition of a patient is likely to stabilize and even improve for the duration of the treatment. Panels (**E**,**F**): ACH-based drugs in late AACD. The patient is asymptomatic for AD but a substantial neuronal fraction has crossed the T1 threshold and was rendered insensitive to ACH-based drugs; these neurons eventually cross the T2 threshold and commit apoptosis. In panel (**E**), the drug reduces the rate of AβPP-derived *i*Aβ accumulation. The initially sub-T1 neurons cross the T1 and proceed toward the T2 threshold and eventual apoptosis but with a considerable delay. In panel (**F**), the rate of AβPP-derived *i*Aβ accumulation is reversed. The sub-T1 neurons are protected for the duration of the treatment but the overall effect in panels (**E**,**F**) is insignificant.

In virtually any disease, the earliest possible intervention is the most beneficial. This is certainly the case with AACD. The essence of the treatment of AACD is either the reduction in the rate of accumulation of AβPP-derived *i*Aβ or the reversal of its accumulation and the decrease in its levels (the latter is, of course, preferable, but it depends on the efficiency of the drug in suppressing the influx of AβPP-derived *i*Aβ). The sooner after the diagnosis the treatment starts, the lower the AβPP-derived *i*Aβ baseline is, the less neurodegeneration occurs, and the more effective the treatment will be. Figure 7 depicts two possible modes of action of the ACH-based drugs administered at the early and progressively advancing stages of AACD.

In panels A and B of Figure 7, the levels of AβPP-derived *i*Aβ have just crossed the T^0^ threshold in all affected neurons of AACD patients; the condition has commenced and is progressing. It has a long way to go: in a typical AACD case, the extent of the T1 threshold appears to be substantially higher than that of T^0^, as suggested by the observation that AACD patients develop AD only infrequently. Panel A shows the effect of an ACH-based drug that reduces the rate of AβPP-derived *i*Aβ accumulation. The accumulation continues but the *i*Aβ levels increase significantly slower than pre-treatment. Consequently, since the severity of the disease is a function of the levels of AβPP-derived *i*Aβ within the AACD Zone [4], the condition is substantially milder than it would have been if untreated. Panel B of Figure 7 illustrates the effect of an ACH-based drug that reduces the influx of AβPP-derived *i*Aβ sufficiently to reverse its accumulation. Its levels steadily decrease and eventually cross, in reverse, the T^0^ threshold. At this point, the patient is technically cured and remains disease-free for the duration of the treatment. Arguably, this is one of only a few scenarios where the utilization of the current ACH-based drugs could be justified, due to the certainty of the outcomes.

In panels C and D of Figure 7, the levels of AβPP-derived *i*Aβ in the affected neurons of AACD patients have traversed about half the distance between the T^0^ and T1 thresholds. This is the initial state at the time of the administration of a drug. Panel C illustrates the effect of an ACH-based drug that reduces the rate of accumulation of AβPP-derived *i*Aβ. Its levels continue to increase as a function of time but much slower than prior to the commencement of the treatment. Likewise, AACD progresses significantly slower and is milder than it would in the absence of a drug. Moreover, in contrast to an untreated patient, the levels of AβPP-derived *i*Aβ would not cross, as shown, the T1 threshold within the lifetime of an individual (or would cross it much later) and AACD would not evolve into AD (or would with a significant delay). Panel D of Figure 7 depicts the effect of an ACH-based drug that reverses the rate of accumulation of AβPP-derived *i*Aβ and causes a steady decrease in its levels. As a result, the condition of a patient is likely to stabilize and even improve for the duration of the treatment.

Panels E and F of Figure 7 depict a scenario where a fraction of the affected neurons have reached and crossed the T1 threshold but an AACD patient is asymptomatic for AD. The evolution of this initial state in the presence of a drug is more complex than in the two preceding scenarios. Regardless of the effect of an ACH-based drug on the rate of accumulation of AβPP-derived *i*Aβ, it will have no therapeutic effect on the over-T1 neurons because in these cells, the AβPP-independent *i*Aβ production pathway has been already activated. The levels of *i*Aβ generated in this pathway will steadily increase and cross the T2 threshold; cells will commit apoptosis and this neuronal fraction will be lost. Any ACH-based drug would affect only the neurons that are still sub-T1. In panel E of Figure 7, an ACH-based drug suppresses the rate of accumulation of AβPP-derived *i*Aβ but its levels keep increasing. Eventually, they will reach and cross the T1 threshold in all initially sub-T1 neurons. The AβPP-independent *i*Aβ production pathway will be activated, and AACD will evolve into AD. In this scenario, therefore, a fraction of affected neurons would be redeemed by an ACH-based drug but only temporarily. In panel F of Figure 7, on the other hand, an ACH-based drug suppresses the influx of AβPP-derived *i*Aβ sufficiently to reverse the rate of its accumulation and its levels are steadily decreasing. In this scenario, although the over-T1 neuronal fraction will be eventually lost, the sub-T1 neurons will be redeemed for the duration of the treatment. In either case (panels E and F), however, the overall effect of the ACH-based drug would be insignificant.

## 12. Symptoms of AACD-Related Cognitive Impairment Overlap with and Are Indistinguishable from Those of AD-Associated MCI: Ramifications for ACH-Based Drugs Therapy

**Figure 8:**
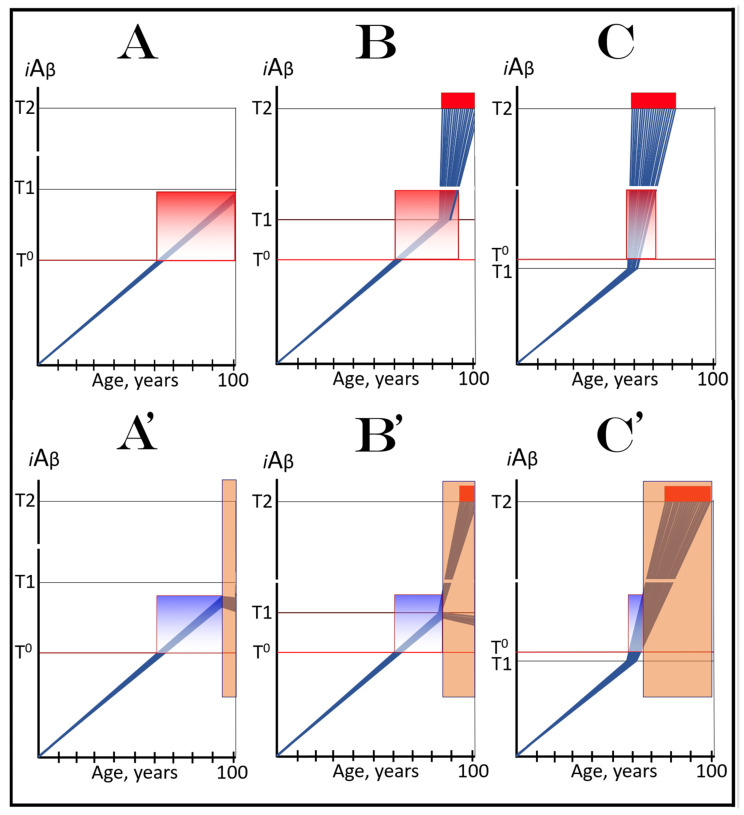
**Symptoms of AACD overlap with and are indistinguishable from those of AD-Associated MCI: Ramifications for ACH-based drugs therapy.** *i*Aβ: Intraneuronal Aβ. *Blue lines*: Levels of *i*Aβ in individual neurons. ***T*^0^*** threshold:* The concentration of *i*Aβ that triggers the neurodegeneration manifesting as AACD. ***T1*** *threshold*: The concentration of AβPP-derived *i*Aβ that mediates elicitation of the ISR and triggers the activation of the AβPP-independent *i*Aβ production pathway. ***T2*** *threshold*: The level of *i*Aβ, produced mainly in the AβPP-independent pathway, which triggers neuronal apoptosis. *Pink gradient boxes*: AACD Zone or its equivalents in terms of the range of concentrations of AβPP-derived *i*Aβ. *Blue boxes*: The defined range of concentrations of AβPP-derived *i*Aβ within AACD Zone or its equivalents. *Red boxes*: Apoptotic Zone, the range of *i*Aβ concentrations that cause the commitment to apoptosis. *Orange boxes*: The duration of administration of an ACH-based drug. Panel (**A**): The T1 threshold is not crossed. AACD commences with the crossing of the T^0^ threshold and continues for the remaining lifespan; the *i*Aβ–caused cognitive impairment is entirely attributable to AACD. Panel (**B**): The T1 threshold is lowered. The same range of *i*Aβ consists of the pre-T1 crossing and post-T1 crossing portions. The symptoms caused by the former are AACD-associated whereas those caused by the latter constitute AD-associated mild cognitive impairment. Panel (**C**): The T1 threshold is further lowered. The same range of *i*Aβ concentrations is wholly post-T1 crossing, and the corresponding symptoms are attributable entirely to AD-associated MCI. Panel (**A’**): ACH-based drug is administered prior to the T1 crossing and protects the entire neuronal population for the duration of the treatment. Panel (**B’**): The drug is administered when a significant neuronal fraction has already crossed the T1 threshold. This fraction is eventually lost; only the sub-T1 neuronal fraction is protected for the duration of the treatment. Panel (**C’**): ACH-based drug is administered within the same *i*Aβ range, but after the entire neuronal population has crossed the T1 threshold; it has no protective effect whatsoever. For details, see the main text.

The AACD Zone is, by definition, a range of *i*Aβ concentrations that trigger the neurodegeneration, which manifests as AACD [4]. The lower boundary of this range is the T^0^ and the upper boundary is the T1 threshold. However, whereas the former is tangible, the latter is not. For the sake of argument, we can define AACD Zone as a range of *i*Aβ concentrations starting at the T^0^ threshold and continuing upward to some arbitrary level. If the upper level happens to be the T1 threshold, the two definitions are identical. Alternatively, we can envision this scenario as the end of the lifespan coinciding with the moment when AβPP-derived *i*Aβ reaches (but does not cross) the T1 threshold, as shown in Figure 8, panel A. But what if the T1 threshold is lower than the chosen upper level, or if AβPP-derived *i*Aβ crosses the T1 threshold within the range defined above? This is an important question because the same symptoms could be attributable to different sources. One situation where this issue arises is depicted in panel B of Figure 8. As we reasoned earlier, upon the T1 crossing, the AβPP-independent *i*Aβ production pathway is activated, AD commences, and AACD morphs into AD. Since, by definition, the extent of the neurodegeneration and, accordingly, the symptoms reflect the *i*Aβ concentrations, the same symptoms would manifest within the same *i*Aβ range (gradient pink boxes; the extents of the T^0^ threshold are identical in all panels of Figure 8). While in panel A, these symptoms can be clearly defined as AACD-related cognitive impairment, in panel B, they are composed of AACD-related impairment and AD-associated mild cognitive impairment (MCI). An even more drastic situation is presented in panel C of Figure 8. In this panel, the extent of the T^0^ threshold is the same as in panels A and B, but it is higher than the T1 threshold. Technically, there is neither an AACD Zone nor an AACD Zone. Here, the range of *i*Aβ concentrations corresponding to that considered in panels A and B consists mostly of *i*Aβ generated independently of AβPP, but since the range (gradient pink box) is the same in panels A, B, and C, so are the symptoms. In panel C, however, the symptoms are attributable, in their entirety, to AD-associated MCI. The point of this discussion is to demonstrate that within a certain range, symptoms of AACD on MCI are overlapping and indistinguishable (they can be properly attributed only by measuring the activity of the AβPP-independent *i*Aβ production pathway; see below). In the above scenarios, the only tangible difference is that when the levels of *i*Aβ cross the T1 threshold, the integrated stress response is elicited. At this point, as discussed above, cellular protein synthesis is reprogrammed and largely inhibited. Eventually, this inhibition would affect learning and long-term memory formation, which require de novo protein synthesis; there is, however, a considerable gap between the elicitation of the ISR and the manifestation of these consequences. It could be argued that the difference in the attribution of similar symptoms to AACD or AD is purely a semantic exercise; however, it is not.

The designations of symptoms as AACD-related cognitive impairment of AD-associated MCI are of great functional importance because they implicate fundamentally different underlying mechanisms. AACD-related impairment is propagated by *i*Aβ produced by AβPP proteolysis, whereas AD-associated MCI is driven by *i*Aβ generated in the AβPP-independent pathway. Thus, the distinction between etiological origins of potentially overlapping symptoms is not semantic but a functional and practical issue, as illustrated in panels A’ through C’ of Figure 8. In panel A’, the treatment with an ACH-based drug is implemented when the levels of AβPP-derived *i*Aβ are within the AACD Zone, i.e., below the T1 and above the T^0^ thresholds (the *i*Aβ range between the T^0^ threshold and the level of the treatment implementation is shown as blue boxes), and the symptoms are, unquestionably, AACD-related cognitive impairment. The ACH-based drug would be very effective in this situation (to simplify the discussion, the “best case scenario” is shown: the drug reverses the accumulation of AβPP-derived *i*Aβ and causes a steady decrease in its levels), levels of AβPP-derived *i*Aβ would steadily decrease for the duration of the treatment, and the condition of the patient would stabilize and potentially improve. In panel B’ of Figure 8, the treatment is administered when the levels of *i*Aβ have reached the same extent as in panel A’. At this time, however, a fraction of the affected neurons have already crossed the T1 threshold and activated the AβPP-independent *i*Aβ production pathway. The drug is effective only in the still sub-T1 neurons; in this neuronal subpopulation, the accumulation of AβPP-derived *i*Aβ is reversed and cells are redeemed for the duration of the treatment. On the other hand, the drug has no effect on the over-T1 neuronal fraction; it reaches the T2 threshold and is eventually lost. In panel C’ of Figure 8, the entire “blue” range is above the T1 threshold, and the patient’s condition is unquestionably AD-associated MCI. Because the AβPP-independent *i*Aβ production pathway has been activated, the drug has no effect; *i*Aβ levels would progress to the T2 threshold and trigger apoptosis in the entire neuronal population. The bottom line is that in panels A’ through C’, the ACH-based drug was administered at the same symptomatic stage but with drastically different consequences. The reason for this is that the treatment was implemented at different conditions (i.e., AACD versus AD), indistinguishable to us due to the current inability to detect the T1 crossing in human patients (practically, the T1 crossing can be ascertained only via the detection of the operation of the AβPP-independent *i*Aβ production pathway, not feasible currently in human subjects). It follows that if a drug would be able to reduce the levels of not only AβPP-derived *i*Aβ but also of *i*Aβ generated independently of AβPP, it would be equally effective in all three situations (panels A’ through C’). Just such a class of drugs, the ACH2.0-based drugs, is, in fact, described below.

## 13. Clinical Trials of ACH-Based Drugs in Prevention of AD with All Participants at Sub-T1 Levels of *i*Aβ: Apparently Straightforward but Impractical

Above, we have established that ACH-based drugs are limited in their utility. Indeed, their short-term employment is ineffective, their long-term engagement is highly problematic, and their implementation at the symptomatic stages of AD is futile. Severe limitations of ACH-based drugs apply not only to their utilization but extend also to the evaluation of their efficiency in clinical trials. The present and two following sections analyze the potential outcomes of clinical trials of ACH-based drugs in the prevention of AD. The decisive variable in such trials is the composition of a trial’s cohorts. The present section considers an ideal scenario and concludes that it is impractical. The following section analyzes the outcomes of a trial with cohorts reflecting the general population of age close to or exceeding the statistical age of the late onset of AD and decides that they could be grossly misleading. Section 15 examines the outcomes of a trial with cohorts composed of high-risk individuals and establishes that they would be either impossible or uninformative.

**Figure 9:**
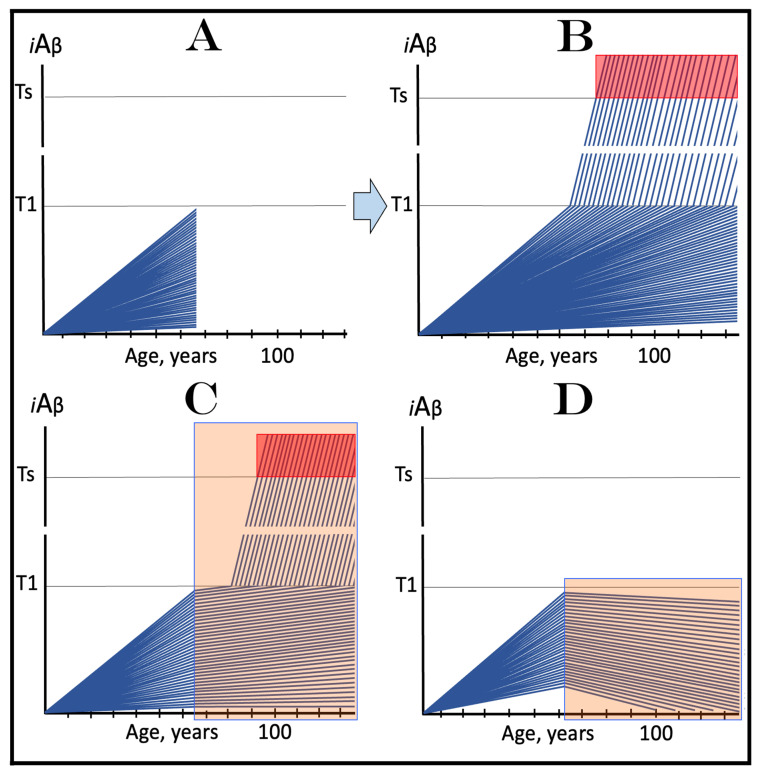
**Clinical trials of ACH-based drugs in prevention of AD with all trial subjects at sub-T1 levels of *i*Aβ.** *i*Aβ: Intraneuronal Aβ. *Blue lines*: The levels of *iA*β in individual trial subjects. ***T1*** *threshold*: The concentration of AβPP-derived *i*Aβ that mediates elicitation of the ISR and triggers the activation of the AβPP-independent *i*Aβ production pathway. ***Ts*** *threshold*: The levels of *i*Aβ that trigger the manifestation of AD symptoms. *Pink boxes*: Symptomatic Zone, the range of *i*Aβ concentrations above the Ts threshold. *Orange boxes:* The duration of administration of an ACH-based drug. Panel (**A**): The initial state of *i*Aβ levels in individual trial subjects at the commencement of the treatment. Panel (**B**): The evolution of the initial state in the untreated individuals (the placebo group). In the majority of subjects, the levels of *i*Aβ do not cross the T1 threshold within the lifespan of an individual. In those subjects where it does, the AβPP-independent pathway of *i*Aβ production is triggered, the levels of *i*Aβ cross the Ts threshold and AD symptoms manifest. Panels (**C**,**D**): The evolution of the initial state in the medicated trial participants. Panel (**C**): The drug lowers the rate of accumulation of AβPP-derived *i*Aβ but its levels keep increasing. In a fraction of participants, they cross the T1 threshold and trigger the AD symptoms. The outcome is principally the same as in the placebo group, but is preceded by a lag period; in another fraction, the delay is sufficient to prevent the disease. Panel (**D**): The drug causes the reversal of the accumulation of AβPP-derived *i*Aβ. Its levels do not cross the **T1** threshold; no disease occurs in trial participants for the duration of the treatment.

Figure 9 illustrates the outcomes of an “ideal” clinical trial of an ACH-based drug in the prevention of AD, with every line representing an individual trial subject. Such a trial is “ideal” in that all its subjects are not just asymptomatic for AD but sub-T1, i.e., the levels of AβPP-derived *i*Aβ in all their neurons are below the T1 threshold. Panel A of Figure 9 shows the initial state of the levels of *i*Aβ in trial subjects immediately prior to the administration of a drug. Panel B depicts the evolution of the initial state in the placebo group. In this cohort, a fraction of trial participants would cross the T1 threshold and activate the AβPP-independent *i*Aβ generation pathway. The levels of *i*Aβ produced in this pathway would rapidly increase. Upon reaching the Ts threshold (Ts signifies the symptomatic threshold) and crossing into the Symptomatic Zone (pink box), they would trigger the manifestation of symptoms of AD.

Panels C and D of Figure 9 illustrate the evolution of the initial state in medicated trial subjects (orange boxes show the duration of the treatment). In panel C, the ACH-based drug decreases the influx of AβPP-derived *i*Aβ and thus suppresses the rate of its accumulation. The reduction in the rate of accumulation, however, is not sufficient to reverse it, and the levels of AβPP-derived *i*Aβ keep increasing. Following the T1 crossing, the AβPP-independent *i*Aβ production pathway, which is insensitive to the ACH-based drugs, would be activated and the progression of the disease would be identical to that in the placebo cohort; the manifestation of the symptoms of AD would eventually occur but with a significant delay. In some participants, this delay is sufficient to prevent the occurrence of AD within their lifetimes. In panel D of Figure 9, the reduction in the influx of AβPP-derived *i*Aβ is sufficient to reverse the rate of its accumulation. The levels of AβPP-derived *i*Aβ steadily decrease and would not cross the T1 threshold for the duration of the treatment. In this scenario, the drug would prevent the disease.

In such an “ideal” trial for the prevention of AD, the outcomes would be clear and easily interpretable. Such a trial, however, is impractical. The problem is the selection of trial subjects with levels of AβPP-derived *i*Aβ certainly below the T1 threshold. Sporadic AD symptoms start manifesting, statistically, at about the age of 65. The T1 threshold, however, is crossed long, probably years, before the manifestation of the symptoms [4]. Upon the T1 crossing, the AβPP-independent *i*Aβ generation pathway is activated, which renders ACH-based drugs completely inadequate: they would still suppress the influx of AβPP-derived *i*Aβ but now its contribution to the cellular *i*Aβ pool is only marginal and thus irrelevant to the progression of the disease. Since currently, as discussed above, we lack tools for detecting the T1 crossing in human subjects, the only means to ascertain that over-T1 individuals are not included in a trial is to limit the age of the trial subject to that well below the statistical age of the late onset of AD. This, however, translates into a long wait, possibly a decade or more, for AD cases to develop (at least in the placebo cohort), thus rendering this type of trial impractical. The following two sections address the consequences of the inclusion of asymptomatic over-T1 individuals in clinical trials.

## 14. Clinical Trials of ACH-Based Drugs in Prevention of AD with Participants Representing a Cross-Section of the General Population Aged 65 and Over: Results Could Be Grossly Misleading

### 14.1. The Overall Picture

**Figure 10:**
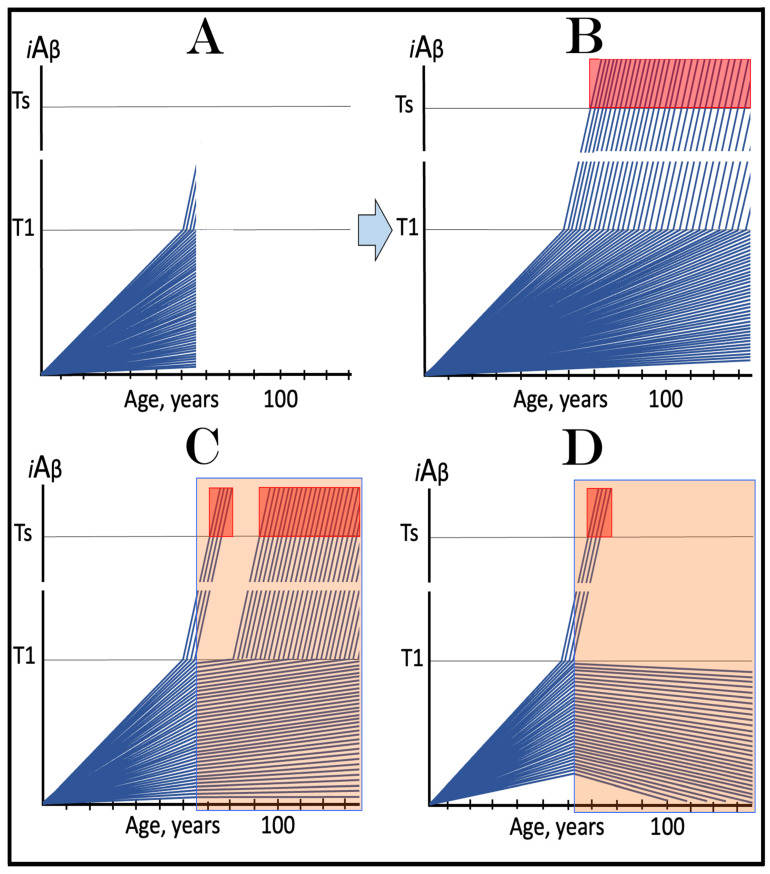
**Clinical trials of ACH-based drugs in prevention of AD with a cross-section of the general population aged 65 and over.*** i*Aβ: Intraneuronal Aβ. *Blue lines*: The levels of *i*Aβ in individual trial subjects. ***T1*** *threshold*: The concentration of AβPP-derived *i*Aβ that mediates elicitation of the ISR and triggers the activation of the AβPP-independent *i*Aβ production pathway. ***Ts*** *threshold*: The levels of *i*Aβ that trigger the manifestation of AD symptoms. *Pink boxes*: Symptomatic Zone, the range of *i*Aβ concentrations above the Ts threshold. *Orange boxes:* The duration of administration of an ACH-based drug. Panel (**A**): The initial state of *i*Aβ levels in individual trial subjects at the commencement of the treatment. Importantly, in a fraction of the subjects, these levels have crossed the T1 threshold. Panel (**B**): The evolution of the initial state in the untreated individuals (the placebo cohort). It is principally identical to that shown in panel B of Figure 9, except it occurs faster. Panels (**C**,**D**): The evolution of the initial state in the medicated trial participants. Note that in both panels, the initial over-T1 fraction is not affected by the drug due to the operation of the AβPP-independent *i*Aβ production pathway. Panel (**C**): In the initial sub-T1 fraction, the rate of accumulation of AβPP-derived *i*Aβ is lowered but its levels keep increasing. After a delay (in comparison with the placebo group), they cross the **T1** threshold and eventually reach the Symptomatic Zone in a fraction of the subjects. In another fraction, the delay is sufficient to prevent the disease. Panel (**D**): In the initial sub-T1 fraction, the drug causes the reversal of the accumulation of AβPP-derived *i*Aβ. Its levels do not cross the **T1** threshold; no disease occurs in these trial participants for the duration of the treatment. However, in both panels (**C**,**D**), the initial over-T1 neuronal fraction progresses into the Symptomatic Zone, unaffected by the drug. Therefore, for a considerable duration, the trial outcomes in the treated cohort would be nearly identical to the outcomes in the placebo cohort thus obscuring the effect of the drug.

The present section considers clinical trials of ACH-based drugs in the prevention of AD with participants representing a cross-section of the general population close to and over the statistical age of the late onset of AD (about 65 years). At this age, only a minor fraction of the population is going to develop AD (about 10% at 65 and increasing with age). All trial subjects are asymptomatic for AD. It is, however, inevitable that in some the levels of AβPP-derived *i*Aβ have already crossed the T1 threshold, and the AβPP-independent *i*Aβ production pathway has been already activated. Such an initial state is depicted in panel A of Figure 10. The evolution of this initial state in the placebo cohort, shown in panel B of Figure 10, is identical to that presented in Figure 9 above. In some participants, the levels of AβPP-derived *i*Aβ will not cross the T1 threshold within their lifetime. In those trial subjects whose levels of AβPP-derived *i*Aβ will cross the T1 threshold, the AβPP-independent *i*Aβ generation pathway would become operational, and its product will rapidly accumulate, cross the Ts threshold, and trigger the manifestation of the symptoms of AD.

The evolution of the initial state in the medicated (orange boxes) subjects (panels C and D of Figure 10) is complex. With the exception of cases where *i*Aβ levels were over T1 prior to the drug administration, it is the same as presented in the preceding section. In panel C, the ACH-based drug decreases the influx of AβPP-derived *i*Aβ and thus reduces the rate of its accumulation. The reduction, however, is not sufficient to reverse it, and the levels of AβPP-derived *i*Aβ continue to increase. Following the T1 crossing, the AβPP-independent *i*Aβ production pathway, which is insensitive to the ACH-based drugs, would be activated and the progression of the disease would be identical to that in the placebo cohort; the manifestation of the symptoms of AD would eventually occur but with a significant delay. In some participants, this delay is sufficient to prevent the occurrence of AD within their lifetimes. In panel D of Figure 10, the reduction in the influx of AβPP-derived *i*Aβ is sufficient to reverse the rate of its accumulation, and its levels steadily decrease. They would not cross the T1 threshold and AD would not occur for the duration of the treatment. In both scenarios, however, the drug would be ineffective in initially over-T1 trial subjects. In these cases, the progression of AD would continue uninterrupted, and the levels of *i*Aβ, produced mainly independently of AβPP, would cross the Ts threshold and trigger the manifestation of AD symptoms. Therefore, for a significant period of time, measured in years, the outcomes in the drug recipients would closely resemble the outcomes in the placebo cohort. Since in asymptomatic initially over-T1 medicated individuals, a fraction of neurons could be still sub-T1 and therefore would be protected by the drug for the duration of the treatment, AD symptoms may develop marginally slower than in the placebo group. But, even in such a case, the actual effect of an ACH-based drug would be obscured or distorted by the outcome of a trial. Eventually, the outcomes would diverge but the wait could be impractically long. In view of the importance of these considerations, they are analyzed further and in greater detail in the following sub-section below.

### 14.2. Detailed Analysis: Duration of Trials Could Be Prohibitively Excessive Prior to the Divergence of the Outcomes in Medicated and Placebo Groups

**Figure 11:**
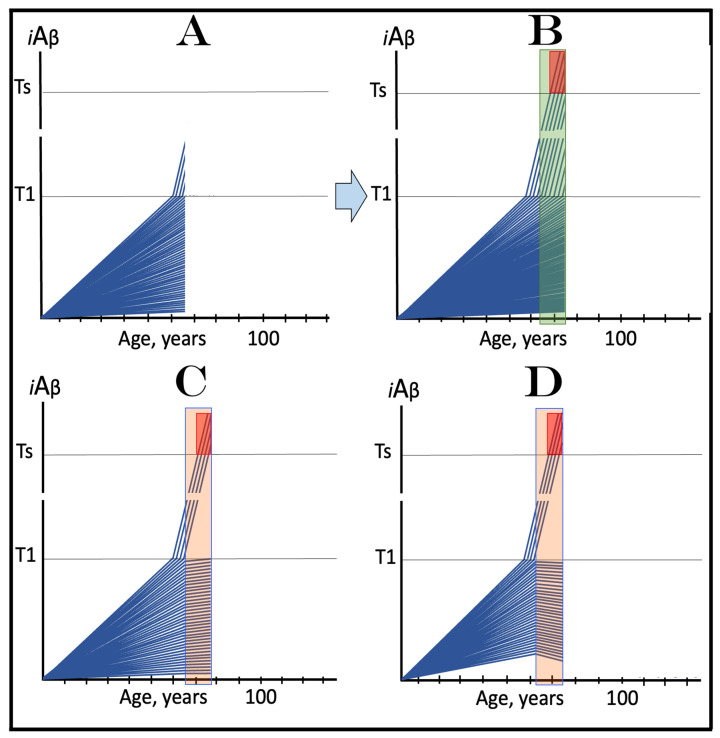
**Clinical trials of ACH-based drugs in prevention of AD with a cross-section of the general population: a detailed analysis.*** i*Aβ: Intraneuronal Aβ. *Blue lines*: The levels of *i*Aβ in individual trial subjects. ***T1*** *threshold*: The concentration of AβPP-derived *i*Aβ that mediates elicitation of the ISR and triggers the activation of the AβPP-independent *i*Aβ production pathway. ***Ts*** *threshold*: The levels of *i*Aβ that trigger the manifestation of AD symptoms. *Pink boxes*: Symptomatic Zone, the range of *i*Aβ concentrations above the Ts threshold. *Orange boxes:* The duration of administration of an ACH-based drug. *Green box:* The duration of the evolution of the initial over-T1 fraction of trial subjects (“time interval of concern”). Panel (**A**): The initial state of *i*Aβ levels in individual trial subjects at the commencement of the treatment. Importantly, in a fraction of the subjects these levels have crossed the T1 threshold. Panel (**B**): Evolution of the initial state in the placebo cohort for the duration of the time interval of concern. The initial over-T1 fraction crosses the Ts threshold and the AD symptoms manifest. During this time interval, additional participants cross the T1 threshold but none reaches the Ts threshold. Panels (**C**,**D**): The evolution of the initial state in the medicated cohort during the time interval of concern. In both panels the initial over-T1 fraction crosses into the Symptomatic Zone and AD symptoms manifest. No additional crossings of the T1 threshold occur during the period of interest. The outcomes in the medicated and placebo cohorts within the time interval of concern are differentiated only by the presence in the placebo group of additional participants who crossed the T1 threshold but remain asymptomatic and thus “invisible” in the analysis of the results.

To better understand the nature of the overlap in the evolution of the medicated and placebo cohorts in a scenario considered above, this sub-section examines a limited time interval (the “time interval of concern”): from the commencement of the drug administration until all initially over-T1 subjects cross the Ts threshold and enter the Symptomatic Zone. This analysis is illustrated in Figure 11. Panel A of Figure 11 depicts the initial state. Its main attribute of concern is the inclusion of asymptomatic subjects whose levels of *i*Aβ are over T1 and where the AβPP-independent *i*Aβ production pathway has already been activated. Panel B depicts the evolution of the initial state in the placebo cohort within the time interval of concern (green box). In the initially over-T1 subjects, *i*Aβ levels steadily increase, subjects enter the Symptomatic Zone, and AD symptoms manifest. Within the same time interval of concern, the levels of AβPP-derived *i*Aβ cross the T1 threshold in multiple additional participants from the placebo cohort. In these trial participants, the AβPP-independent *i*Aβ generation pathway is activated and the second AD stage commences. Importantly, by the conclusion of the time interval of concern, the levels of *i*Aβ (produced mainly independently of AβPP) have not yet reached the Ts threshold in the initially sub-T1 trial subjects.

In panels C and D of Figure 11, the administration of an ACH-based drug prevents the crossings of the T1 threshold in the initially sub-T1 trial subjects for the time interval of concern. Within the same time period, however, the initially over-T1 individuals cross the Ts threshold and enter the Symptomatic Zone at nearly the same rate as in the placebo group. At the conclusion of the time interval of concern, there is, apparently, a marked distinction between the medicated and the placebo cohorts: In both, the initially over-T1 subjects entered the Symptomatic Zone and developed AD symptoms, but only in the latter did additional numerous subjects cross the T1 threshold. These additional subjects, however, did not reach the Ts threshold. They remain asymptomatic and thus “invisible” in the evaluation of the outcomes of the trial. Therefore, within the time interval of concern, the outcomes in the medicated and the placebo cohorts would be similar, possibly indistinguishable, despite the efficiency of a drug. This time interval could be of a substantial duration. Following it, when the “additional” placebo cases enter the Symptomatic Zone, the outcomes would diverge drastically. But were the trial terminated within the time interval of concern, it would be considered a failure.

## 15. Clinical Trials of ACH-Based Drugs in Prevention of AD with High-Risk Participants: Practically Impossible and Self-Defeating

**Figure 12:**
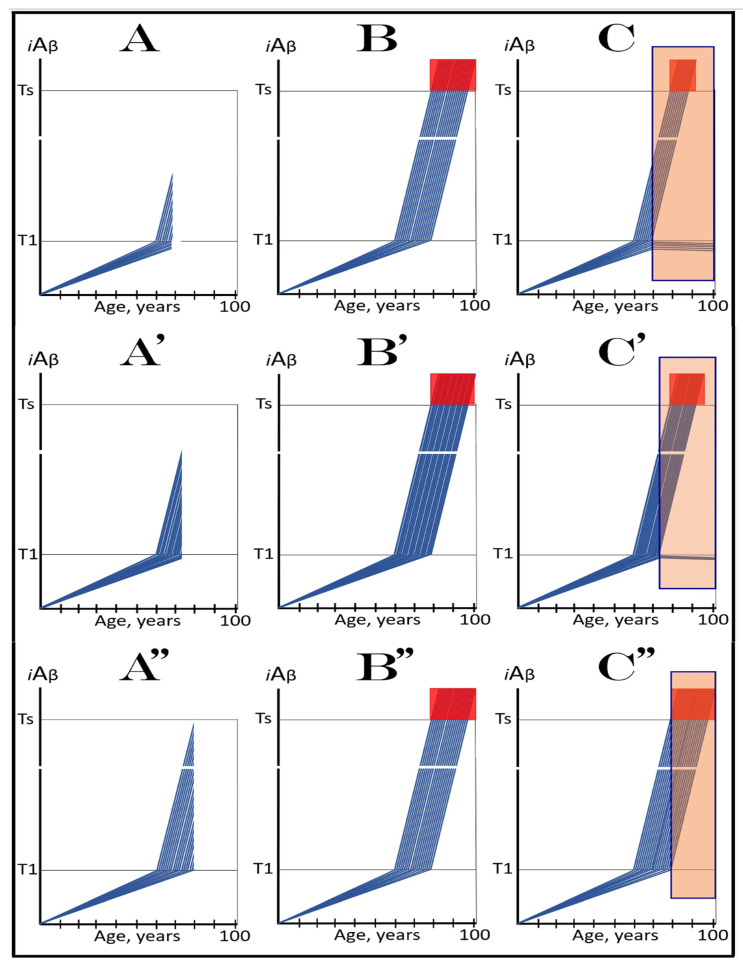
**Clinical trials of ACH-based drugs in prevention of AD with high-risk cohorts.*** i*Aβ: Intraneuronal Aβ. *Blue lines*: The levels of *i*Aβ in individual trial subjects. ***T1*** *threshold*: The concentration of AβPP-derived *i*Aβ that mediates elicitation of the ISR and triggers the activation of the AβPP-independent *i*Aβ production pathway. ***Ts*** *threshold*: The levels of *i*Aβ that trigger the manifestation of AD symptoms. *Pink boxes*: Symptomatic Zone, the range of *i*Aβ concentrations above the Ts threshold. *Orange boxes*: The duration of administration of an ACH-based drug. Panel (**A**): All trial subjects are asymptomatic but about 50% have crossed the T1 threshold and activated the AβPP-independent *i*Aβ production pathway. Panel (**B**): The evolution of the initial stage in the placebo group. The levels of AβPP-derived *i*Aβ cross the T1 threshold and the AβPP-independent *i*Aβ generation pathway becomes operational in all trial subjects. *i*Aβ levels increase and cross the Ts threshold, subjects enter the Symptomatic Zone and AD symptoms manifest. Panel (**C**): The evolution of the initial state in the presence of an ACH-based drug (orange box). To simplify the discussion, only the best-case scenario is considered: a drug that is capable of reversing the accumulation of AβPP-derived *i*Aβ and causing a steady decline in its levels. In the sub-T1 trial subjects, the influx of AβPP-derived *i*Aβ is suppressed and its rate of accumulation is reversed. These subjects would not cross the T1 threshold for the duration of the treatment. However, the drug would be ineffective in the initially over-T1 trial participants. Those would progress toward the Ts threshold, enter the Symptomatic Zone, and AD symptoms would manifest at a rate similar to or indistinguishable from that in the placebo group. The outcomes in the medicated and the placebo group would eventually diverge but only after an impracticably long period of overlap. Panels (**A’**–**C’**,**A”**–**C”**) reiterate the same sequence as shown in panels A through C but with increasing proportions of asymptomatic over-T1 subjects: 75% in (**A’**–**C’**) and 100% in (**A”**–**C”**). In both cases, the evolutions of the initial states in the absence and the presence of the ACH-based drug are similar to those described above but the overlap between the outcomes in the medicated and placebo cohorts increases in the former and the two are practically indistinguishable in the latter, reflecting the notion that selection of high-risk subjects for AD-preventive trials of ACH-based drugs is self-defeating.

The problem with the currently ongoing clinical trials of lecanemab and donanemab in the prevention of AD is that they employ, and apparently are limited to, high-risk participants [138]. Importantly, this problem is not specific to lecanemab and donanemab but is general for any ACH-based drug. The high-risk individuals are asymptomatic persons who, by a number of indicators, would develop AD symptoms shortly or imminently. They are also the individuals who, highly probably, have already crossed the T1 threshold. Whereas in a cross-section of the general population aged 65 or over, considered above, the initially over-T1 asymptomatic individuals represent only a few percentage points, in a high-risk cohort, they would constitute the majority of, if not the entire cohort. The consequences of the utilization of high-risk asymptomatic persons in clinical trials of ACH-based drugs in the prevention of AD are considered in Figure 12.

Three parts of Figure 12 (upper, middle, and bottom) depict three situations with different proportions of asymptomatic over-T1 subjects in a selected cohort (the initial state). In the upper part of Figure 12, the initial state is such that about half of the selected participants are already over T1 (panel A). In these subjects, the AβPP-independent *i*Aβ production pathway has been activated but the levels of *i*Aβ (produced at this stage mainly independently of AβPP) have not yet reached the Ts threshold. Panel B shows the evolution of the initial stage in the placebo group. The levels of AβPP-derived *i*Aβ cross the T1 threshold in all trial subjects. The AβPP-independent *i*Aβ generation pathway becomes operational, *i*Aβ levels increase and cross the Ts threshold, subjects enter the Symptomatic Zone and AD symptoms manifest (provided a sufficiently long lifespan). Panel C depicts the evolution of the initial state in the presence of an ACH-based drug (orange box). To simplify the discussion, only the best-case scenario is considered: a drug that is capable of reversing the accumulation of AβPP-derived *i*Aβ and causing a steady decline in its levels. In the sub-T1 trial subjects, the influx of AβPP-derived *i*Aβ is suppressed and its rate of accumulation is reversed. These subjects would not cross the T1 threshold for the duration of the treatment. However, the drug would be ineffective in the initially over-T1 trial participants. Those would progress toward the Ts threshold, enter the Symptomatic Zone, and AD symptoms would manifest at a rate similar to or indistinguishable from that in the placebo group. The outcomes in the medicated and the placebo group would eventually diverge but only after an impracticably long, well over a decade, period of overlap.

In the middle and the bottom parts of Figure 12, the proportion of asymptomatic but over-T1 trial subjects in the initial state increases. In the middle part of Figure 12, it is about three-quarters of the cohort (panel A’); in the bottom part of Figure 12, it is the entire cohort (panel A”). The evolution of the initial state in the placebo groups for the middle and the bottom parts of Figure 12, shown in panels B’ and B”, respectively, is no different from that depicted in panel B. In the initially over-T1 subjects the disease progresses, and the sub-T1 participants cross the T1 threshold. Eventually, all subjects reach the Ts threshold, cross into the Symptomatic Zone, and AD symptoms manifest. The evolution of the initial state in the presence of the drug (orange boxes) for the middle and the bottom parts of Figure 12 is shown in panels C’ and C”, respectively. In panel C’, the drug is ineffective in three-quarters of the trial subjects who already have crossed the T1 threshold and activated the AβPP-independent *i*Aβ production pathway. Only the initially sub-T1 subjects are protected for the duration of the treatment. However, the overlap between the outcomes in the medicated and placebo cohorts is even greater than that shown in panel C. In panel C”, the entire cohort is largely unresponsive to the ACH-based drug. The evolution of the initial state is principally the same as in the placebo group, and it would not be conceptually possible to gain any information regarding the efficiency of the drug. It should be emphasized here that the composition of high-risk cohorts would differ from trial to trial but, on average, it would most resemble that depicted in the lower panel of Figure 12 (panel A”). The bottom line for this and the preceding sections is that the selection of low-risk subjects for clinical trials of the ACH-based drugs in the prevention of AD is impractical, whereas the selection of high-risk trial participants is self-defeating.

## 16. Clinical Trials of ACH-Based Drugs in Treatment of AACD

### 16.1. Clinical Trials of ACH-Based Drugs in Treatment of AACD with Subjects including Late-Stage Patients: Potential Complications

**Figure 13:**
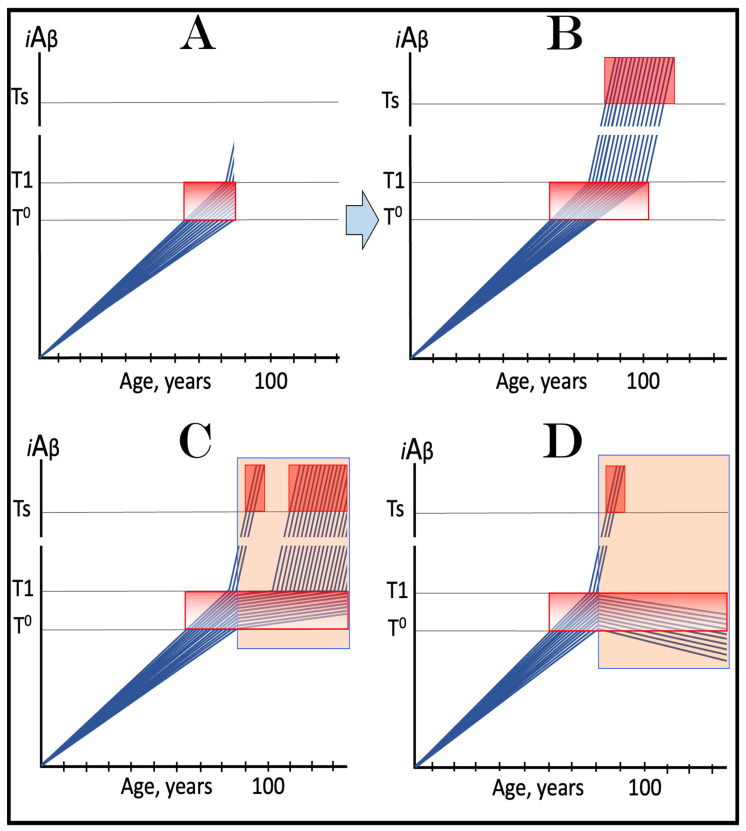
**Clinical trials of ACH-based drugs in treatment of AACD with subjects including late-stage patients.*** i*Aβ: Intraneuronal Aβ. *Blue lines*: The levels of *iA*β in individual trial subjects. ***T^0^*** *threshold*: The concentration of *i*Aβ that triggers the neurodegeneration manifesting as AACD. ***T1*** *threshold*: The concentration of AβPP-derived *i*Aβ that mediates elicitation of the ISR and triggers the activation of the AβPP-independent *i*Aβ production pathway. ***Ts*** *threshold*: The levels of *i*Aβ that trigger the manifestation of AD symptoms. *Pink gradient boxes*: AACD Zone, the range of concentration of AβPP-derived *i*Aβ between the T^0^ and T1 boundaries. *Pink boxes*: Symptomatic Zone, the range of *i*Aβ concentrations above the Ts threshold. *Orange boxes*: The duration of administration of an ACH-based drug. Panel (**A**): The initial state. All subjects exhibit the AACD symptoms and are asymptomatic for AD. In the majority of trial participants, the levels of *i*Aβ are confined between the boundaries of the T^0^ and T1 thresholds. In a fraction of trial subjects, the levels of *i*Aβ have crossed the T1 threshold and triggered the activation of the AβPP-independent *i*Aβ production pathway. Panel (**B**): The evolution of the initial state in the placebo group. The initial sub-T1 fraction of the trial subjects crossed the T1 threshold; the AβPP-independent *i*Aβ production pathway is now operative in all participants. *i*Aβ rapidly accumulates, crosses the Ts threshold, and the AD symptoms manifest. Panels (**C**,**D**): The evolution of the initial state in the medicated trial subjects. In both panels, the drug has no effect in the initial over-T1 fraction; in these subjects, AD progresses and, when the Ts threshold is crossed, its symptoms manifest. Panel (**C**): The drug lowers the rate of accumulation of AβPP-derived *i*Aβ. In a fraction of subjects, this is sufficient to prevent the T1 crossing and the occurrence of AD. In another fraction of the trial participants, the T1 threshold is crossed and AD ensues but with a considerable delay. Panel (**D**): The drug reverses the rate of AβPP-derived *i*Aβ accumulation. The T1 threshold would not be crossed and AD would not occur in the initial sub-T1 fraction. Moreover, if/when the T^0^ threshold would be reverse-crossed, the patients would be technically cured. However, because of the presence of the initially over-T1 subjects, the outcomes in the medicated and placebo cohorts would overlap for a substantial duration before they diverge drastically.

A clinical trial of a drug for the treatment of a condition is always easier to conduct than a trial for its prevention. This is because the selection of the trial subjects is greatly simplified. Potentially, the trial’s cohort can be composed of patients at different stages of a disease; such inclusiveness may enrich the outcomes and conclusions of a trial. This, however, is not the case with clinical trials of ACH-based drugs in the treatment of AACD. The potential problem with such a trial arises if it includes late-stage patients. The consequences of such inclusion are illustrated in Figure 13.

If the criterion for selection of the trial subjects is solely the diagnosis of AACD, regardless of its stage, it is inevitable that the trial cohort would include late-stage AACD patients who are asymptomatic for AD but nevertheless have the *i*Aβ levels exceeding the T1 threshold (over-T1 trial subjects). The initial state of such a scenario is depicted in panel A of Figure 13. In all subjects, the levels of AβPP-derived *i*Aβ have crossed the T^0^ threshold and all exhibit AACD symptoms (pink gradient box). A fraction of the late-stage patients has also crossed the T1 threshold and activated the AβPP-independent *i*Aβ generation pathway but remains asymptomatic for AD. The evolution of this initial state in the placebo group is shown in panel B. The levels of AβPP-derived *i*Aβ increase, cross the T1 threshold, and trigger the activation of AβPP-independent *i*Aβ production pathway in all initially sub-T1 patients (provided a sufficient lifespan). The levels of *i*Aβ, produced now mainly independently of AβPP, reach and cross the Ts threshold, and AD symptoms manifest.

Panels C and D of Figure 13 illustrate the evolution of the initial state in the presence of an ACH-based drug (orange boxes). The drug is effective only in patients who were still sub-T1 at the time of its administration and can yield two possible outcomes. In panel C, the drug reduces the influx of AβPP-derived *i*Aβ, the rate of its accumulation decreases but its levels are still increasing. In a fraction of the trial subjects, the decrease in the rate of AβPP-derived *i*Aβ accumulation is sufficient to prevent the T1 crossing (and AD) within the lifetime of a patient. In the rest of the initially sub-T1 subjects, the levels of AβPP-derived *i*Aβ would cross the T1 threshold, the AβPP-independent *i*Aβ production pathway would be activated, and AD would ensue but with a considerable delay in comparison with the placebo group. In panel D of Figure 13, the reduction in the influx of AβPP-derived *i*Aβ is sufficient to reverse its accumulation. Its levels are steadily decreasing for the duration of the treatment. There will be no T1 crossing and no AD in these subjects. Moreover, their condition would improve and some of them will be even cured upon the reverse crossing of the T^0^ threshold.

However, the drug will be inefficient in the initially over-T1 subjects because of the operation of the AβPP-independent *i*Aβ production pathway, which is insensitive to the ACH-based drugs. In these subjects, the levels of *i*Aβ (produced mostly independently of AβPP) would increase and eventually cross the Ts threshold. Consequently, this fraction of trial participants would enter the Symptomatic Zone and exhibit AD symptoms at a rate similar to that in the placebo group. The problem with such clinical trial, therefore, is akin to that discussed above: the outcomes in the medicated and the placebo cohorts would be similar for a substantial duration, measured in years, before diverging, and would obscure and distort the actual effect of the drug.

### 16.2. Clinical Trials of ACH-Based Drugs in Treatment of Early AACD: The Only Unproblematic Clinical Trials of ACH-Based Drugs

**Figure 14:**
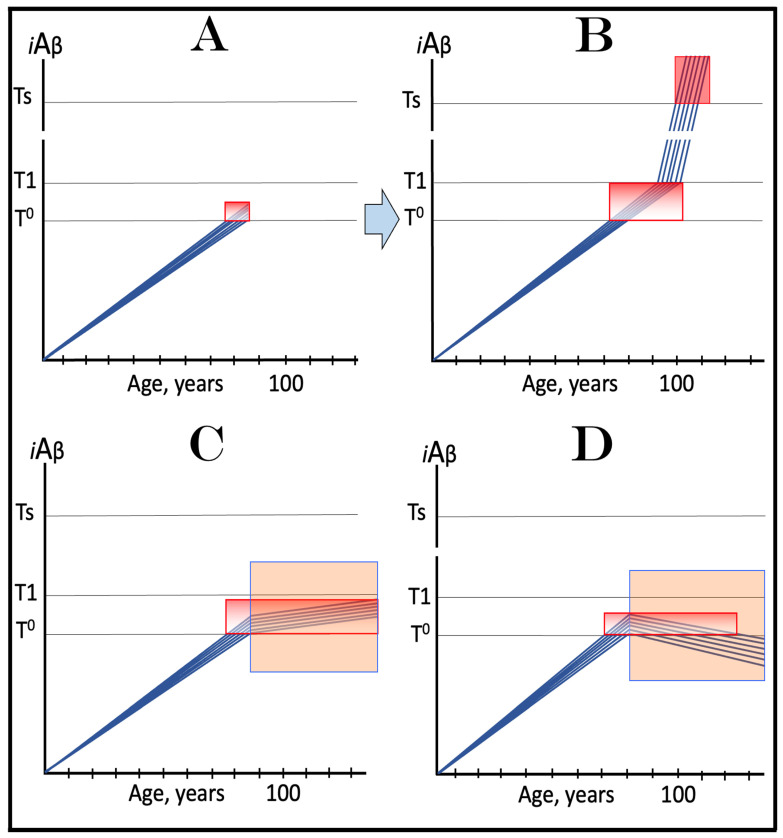
**Clinical trials of ACH-based drugs in treatment of early AACD.*** i*Aβ: Intraneuronal Aβ. *Blue lines*: The levels of *i*Aβ in individual trial subjects. ***T*^0^** *threshold*: The concentration of *i*Aβ that triggers the neurodegeneration manifesting as AACD. ***T1*** *threshold*: The concentration of AβPP-derived *i*Aβ that mediates elicitation of the ISR and triggers the activation of the AβPP-independent *i*Aβ production pathway. ***Ts*** *threshold*: The levels of *i*Aβ that trigger the manifestation of AD symptoms. *Pink gradient boxes*: AACD Zone, the range of concentration of AβPP-derived *i*Aβ between the T^0^ and T1 boundaries. *Pink boxes*: Symptomatic Zone, the range of *i*Aβ concentrations above the Ts threshold. *Orange boxes*: The duration of administration of an ACH-based drug. Panel (**A**): The initial state. All trial participants exhibit only early AACD symptoms; the AβPP-derived *i*Aβ levels have just crossed the T^0^ threshold. Panel (**B**): The evolution of the initial state in the untreated trial subjects (the placebo group). AβPP-derived *i*Aβ steadily accumulates. If and when its levels cross the T1 threshold (subject to the longevity of trial participants), AD ensues. Panels (**C**,**D**): The evolution of the initial state in participants treated with an ACH-based drug. Panel (**C**): The drug reduces the rate of accumulation of the AβPP-derived *i*Aβ and its levels do not reach the T1 threshold for the duration of the treatment. AACD progresses, but much slower than in the absence of the drug. Panel (**D**): The drug reverses the rate of AβPP-derived *i*Aβ accumulation and its levels are steadily decreasing. The condition of the patients is expected to improve and, when the *i*Aβ levels reverse-cross the T^0^ threshold, they will be technically cured. In this trial there is no overlap between the outcomes in the medicated and the placebo cohorts; it is the only type of clinical trial of ACH-based drugs (due to the feasibility of selection of the trial subjects that are highly likely to be sub-T1) that is capable of generating clear and unequivocal results.

It follows that the reliable approach to conducting clinical trials of ACH-based drugs in the treatment of AACD is to employ only trial subjects at the early AACD stages. Because, as discussed above, the extent of the T1 threshold appears to be substantially greater than that of the T^0^ threshold, the inclusion of the over-T1 trial subjects is highly unlikely if only the early-stage AACD patients are selected. This approach and its possible outcomes are illustrated in Figure 14. Panel A depicts the initial state. All selected subjects have crossed the T^0^ threshold and are exhibiting the early symptoms of AACD. Panel B of Figure 14 illustrates the evolution of the initial state in the placebo group. AβPP-derived *i*Aβ continues to accumulate and the condition progresses in all subjects in the placebo cohort. Eventually, provided a sufficiently long lifespan, the levels of AβPP-derived *i*Aβ would cross the T1 threshold and trigger the activation of the AβPP-independent *i*Aβ production pathway and the commencement of AD. The rate of accumulation of *i*Aβ (now produced mostly independently from AβPP) would increase. Its levels would reach and cross the Ts threshold in all participants in the placebo group and AD symptoms would manifest.

Panels C and D of Figure 14 depict diagrammatically the evolution of the initial state in the presence of an ACH-based drug. In panel C, the drug reduces the influx of AβPP-derived *i*Aβ and suppresses its rate of accumulation. Its levels, however, continue to increase albeit much slower than prior to the drug administration. The T1 threshold would not be crossed and a low-grade, rather mild, AACD would persist for the remaining portions of the lifespans of treated trial subjects or for the duration of the treatment. In panel D of Figure 14, the reduction in the influx of AβPP-derived *i*Aβ is sufficient to reverse its accumulation, and its levels are steadily decreasing. The condition of the medicated subjects would be improving. Moreover, when the levels of AβPP-derived *i*Aβ would reverse-cross the T^0^ threshold, the patients would be technically cured (subject to full restoration of neuronal functionality). It should be emphasized that this scenario, i.e., trials of treatment of early AACD, is the only type of clinical trial of the ACH-based drugs that is capable of generating clear and unequivocal results.

## 17. The ACH2.0-Guided Next Generation Therapeutic Strategy for AD and AACD: The Depletion of *i*Aβ via Its Targeted Degradation

### 17.1. Effective AD Therapy Requires Both the Reduction of the Levels of iAβ and Cessation of the Operation of the AβPP-Independent Pathway of Its Production

To briefly summarize the preceding sections, the ACH-based drugs could be highly problematic both in their utilization and in their evaluation. The focal point of all principal problems with ACH-based drugs is the occurrence of the operational AβPP-independent *i*Aβ generation pathway. Indeed, as discussed above, the ACH-based drugs are rendered completely ineffective as soon as the AβPP-independent *i*Aβ production pathway is activated. That the ACH-based drugs would be effective were it not for the operation of the AβPP-independent *i*Aβ generation pathway is exemplified by numerous studies with transgenic mice lacking the operational AβPP-independent *i*Aβ production pathway and overexpressing Aβ solely in the AβPP proteolytic pathway, where the suppression of its production or the removal of extracellular Aβ (both leading to the reduction in the levels of *i*Aβ) relieved or reversed the neurodegeneration [8,9,10]. These results are consistent with and strongly support the major tenet of the ACH2.0, namely that *i*Aβ, produced in AD mainly in the AβPP-independent pathway, triggers and drives the neurodegeneration and that its depletion would be therapeutically beneficial. Thus, the first component of the ACH2.0-guided Next Generation Therapeutic Strategy for AD and AACD is the reduction in the levels of total *i*Aβ, regardless of its origin. This, however, is not enough, because the continuous and very efficient production of *i*Aβ in the AβPP-independent pathway would be unceasingly replenishing its levels. Therefore, for the Next Generation Therapeutic Strategy to be effective, another component needs to be enacted: cessation of the operation of the AβPP-independent *i*Aβ generation pathway. Propitiously, as reasoned below, achieving the former accomplishes the latter.

### 17.2. A Sufficient Depletion of iAβ Would Cease the Activity of the AβPP-Independent iAβ Production Pathway

Indeed, the activation and operation of the AβPP-independent *i*Aβ production pathway requires sufficiently high, more specifically over-T1, levels of *i*Aβ. Those are initially supplied by the AβPP proteolysis. When the levels of AβPP-derived *i*Aβ cross the T1 threshold, the AβPP-independent *i*Aβ production pathway is activated and transformed into the AD Engine. This is because the increasing levels of *i*Aβ stimulate its own AβPP-independent production and thus perpetuate the operation of the Engine. As discussed elsewhere [1,4], of several ways to stop the operation of the AβPP-independent *i*Aβ production pathway (regardless of the nature of its underlying mechanism), only one is viable and feasible: the depletion of *i*Aβ below the T1 threshold. With insufficient *i*Aβ levels, the operation of the AβPP-independent pathway of its production would cease. The production of *i*Aβ in the AβPP proteolytic pathway, on the other hand, would continue, and as soon as AβPP-derived *i*Aβ levels are restored to the T1 threshold, they would re-ignite the AD Engine. The accumulation of AβPP-derived *i*Aβ is, however, a very slow process: it takes decades to build up its levels to the T1 threshold in sporadic AD cases, and in the majority of the population, the T1 threshold is not reached (and AD does not occur) within the lifetime of an individual. Therefore, if the *i*Aβ levels were depleted sufficiently deep, close to its initial baseline, its accumulation would resume, solely in the AβPP proteolytic pathway, from a low starting point. This would provide additional decades of AD- and AACD-free lifetime. In fact, both conditions would be unlikely to occur (if the treatment were preventive) or recur (if the treatment were curative) within an individual’s lifespan. Thus, the Next Generation Therapeutic Strategy can be enacted through the substantial depletion of *i*Aβ via its targeted degradation by the drugs specifically designed for this purpose, the ACH2.0-based drugs.

### 17.3. Inhibition of the ISR Is Not a Viable Option for AD

#### In a Fraction of AD Cases, the Effective Therapy May Require Anti-Inflammatory Treatment in Addition to the Depletion of iAβ

In terms of ACH2.0, the elicitation of the integrated stress response in neuronal cells results in the production of the component(s) that are necessary and sufficient for the activation of the AβPP-independent *i*Aβ production pathway and the commencement of AD. It follows that the elicitation of the ISR by means other than AβPP-derived *i*Aβ-mediated activation of eIF2α kinases could be sufficient to activate the AβPP-independent *i*Aβ generation pathway and trigger the disease. In such a scenario, the depletion of *i*Aβ below the T1 threshold alone in AD would not cease the operation of the AβPP-independent *i*Aβ production pathway; the cause of the ISR elicitation should be removed or reduced as well. This scenario is exemplified by the instances of AD in individuals who are predisposed to the disease due to persistent neuronal inflammation (resulting, for example, from a concussion or from multiple concussions). In this category, prior to the activation of the AβPP-independent *i*Aβ production pathway, the long-term anti-inflammatory therapy would protect from the onset of AD (by precluding the elicitation of the ISR and, consequently, of the AβPP-independent *i*Aβ production pathway), whereas after the commencement of AD, the implementation of the transient *i*Aβ depletion via its targeted degradation would be effective if it is paralleled by the long-term anti-inflammatory therapy.

In terms of the ACH2.0, potentially, the activation of the AβPP-independent *i*Aβ generation pathway can be precluded or its operation suppressed, and, accordingly, AD could be prevented or its progression stopped by the inhibition of the ISR, e.g., with the small molecule ISR inhibitor ISRIB. However, due to the ISR’s pivotal role in cellular physiology, its long-term suppression (it would be effective in both prevention and treatment of AD only if implemented long-term) is highly likely to result in widespread adverse effects and is not a viable option.

## 18. ACH2.0-Based Drugs for Prevention and Treatment of AD and AACD: A Definition

In accordance with the preceding section, the ACH2.0-based drugs are the agents that enact or mediate the targeted degradation of *i*Aβ, thus causing its depletion. This class of drugs differs fundamentally from the ACH-based drugs. The latter lower or, hopefully, reverse the rate of accumulation of only AβPP-derived *i*Aβ and do it rather indirectly, “passively”, by limiting its influx. As long as the influx occurs, the ACH-based drugs cannot cause the reduction in AβPP-derived *i*Aβ levels on their own; for this, they rely on the naturally occurring clearance processes. Thus, treatment with the ACH-based drugs can potentially (if the rate of accumulation is reversed) result in a limited depletion of AβPP-derived *i*Aβ, but this depletion is “passive” and, even if it occurs, is apparently insignificant, as suggested by recent results with lecanemab and donanemab [134,135,136,137,138].

In contrast, ACH2.0-based drugs are capable of enacting the “active” depletion of total *i*Aβ, regardless of its origin. The agents can be potentially designed that penetrate inside the neuron, recognize *i*Aβ, and specifically cleave it. Another category of ACH2.0-based drugs includes the agents devised to activate or enhance the intrinsic neuronal activities capable of specific degradation of *i*Aβ. This approach is, in fact, feasible; two highly plausible prime candidates are discussed in Section 26 below. Such degradation is “active” because it is actively forced; in comparison, “passive” degradation is not forced but relies upon physiologically occurring processes. The direct, targeted degradation of *i*Aβ could be substantially more effective than its “passive” counterpart. It is conceivable, therefore, that the treatment with ACH2.0-based drugs could achieve its purpose, i.e., a substantial depletion of *i*Aβ, in a transient manner, within a duration measured in days, akin to that of an antibiotic treatment. As discussed in the following sections, such treatment can be implemented preventively, prior to the manifestation of AD or AACD symptoms; in similarity to their ACH counterparts, ACH2.0-based drugs can also treat symptomatic AACD. Ultimately and uniquely, as described below, ACH2.0-based drugs can deliver the “Holy Grail” of Alzheimer’s research: the efficient treatment of AD at its symptomatic stages. When implemented prior to the T1 crossing, such treatment can be relatively short because, in the absence of the operational AβPP-independent *i*Aβ production pathway, the cellular pool of *i*Aβ would be relatively low. The duration of the treatment would be probably longer in symptomatic AD, where, due to the activity of the AβPP-independent *i*Aβ generation pathway, the levels of *i*Aβ would be significantly higher. In sharp contrast to the ACH-based drugs, their ACH2.0-based counterparts can be easily and unambiguously evaluated in clinical trials for both the prevention and treatment of AD and AACD (see below). Remarkably, as described in the following sections, the effective transient treatment with an ACH2.0-based drug opens up the possibility of its once-in-a-lifetime administration for the prevention of AD and AACD and for their efficient treatment at symptomatic stages.

## 19. Implementation of ACH2.0-Based Drugs in Prevention of AD by Depletion of *i*Aβ via Its Transient, Short-Duration, Targeted Degradation

### 19.1. Prevention of AD by Transient Depletion of iAβ via Its Targeted Degradation in Asymptomatic Low-Risk Individuals with the Entire Neuronal Population Sub-T1

**Figure 15:**
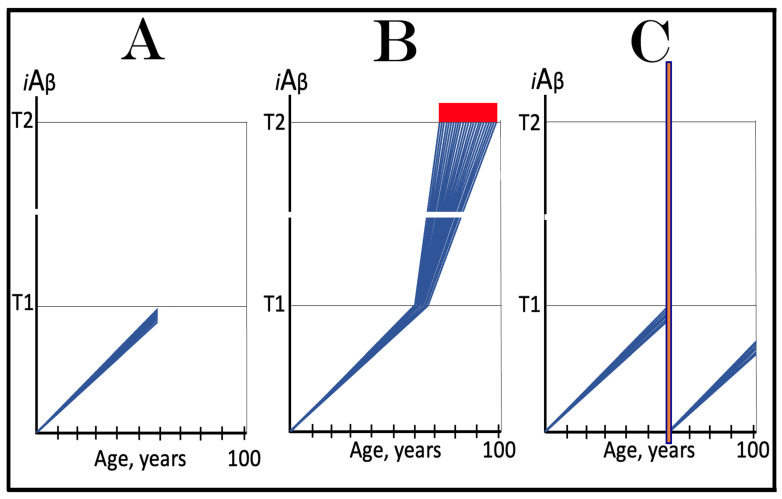
**Prevention of AD by transient depletion of iAβ via its targeted degradation in asymptomatic low-risk individuals.** *i*Aβ: Intraneuronal Aβ. *Blue lines*: Levels of *i*Aβ in individual neurons. ***T1*** *threshold*: The concentration of AβPP-derived *i*Aβ that mediates elicitation of the ISR and triggers the activation of the AβPP-independent *i*Aβ production pathway. ***T2*** *threshold*: The level of *i*Aβ, produced mainly in the AβPP-independent pathway, which triggers neuronal apoptosis. *Red box*: Apoptotic Zone, the range of *i*Aβ concentrations that cause the commitment to apoptosis. *Orange box*: The duration of the transient administration of an ACH2.0-based drug. Panel (**A**): The initial state of the levels of *i*Aβ in the neuronal population of an individual; they all are below the T1 threshold. Panel (**B**): The evolution of the initial state in the absence of the treatment. The levels of *i*Aβ in individual neurons would continue to increase and eventually would cross the T1 threshold. The AβPP-independent *i*Aβ generation pathway would be activated; its product would rapidly accumulate, reach and cross the T2 threshold, and trigger apoptosis. Panel (**C**): The evolution of the initial state following the *i*Aβ depletion treatment with an ACH2.0-based drug. The treatment substantially reduces the levels of *i*Aβ and its accumulation resumes from a low baseline. If the depletion is deep enough and if the treatment were administered mid-life, as shown in the figure, the levels of AβPP-derived *i*Aβ would not reach the T1 threshold within the lifetime of the treated individual. Thus, the single, once-in-a-lifetime-only transient *i*Aβ depletion by an ACH2.0-based drug is potentially capable of protecting from AD for life.

Figure 15 considers the prevention of AD via transient depletion of *i*Aβ by its targeted degradation. In this Figure, it is assumed that not only is an individual asymptomatic for AD but also that the levels of *i*Aβ have not yet crossed the T1 threshold in the entire neuronal population. Moreover, the case under consideration is that of an individual who would, unless preventively treated, develop the disease. To simplify the discussion, it is also assumed that the depletion treatment resets the levels of *i*Aβ completely or nearly completely to the original baseline. Panel A of Figure 15 depicts the initial state of the levels of *i*Aβ in the neuronal population of an individual: they all are below the T1 threshold. Panel B shows the evolution of the initial state in the absence of the treatment. The levels of *i*Aβ in individual neurons would continue to increase and eventually would cross the T1 threshold. The AβPP-independent *i*Aβ generation pathway would be activated; its product would rapidly accumulate, reach and cross the T2 threshold, and trigger apoptosis. Panel C of Figure 15 illustrates the evolution of the initial state following the *i*Aβ depletion treatment with an ACH2.0-based drug. The treatment substantially reduces the levels of *i*Aβ and its accumulation resumes from a low baseline. If the depletion is deep enough and if the treatment were administered mid-life, as shown in the figure, the levels of AβPP-derived *i*Aβ would not reach the T1 threshold within the lifetime of the treated individual. Thus, the single, once-in-a-lifetime-only transient *i*Aβ depletion by an ACH2.0-based drug is potentially capable of protecting from AD for life.

### 19.2. Prevention of AD by Transient Depletion of iAβ via Its Targeted Degradation in Asymptomatic High-Risk Individuals with Over-T1 Neuronal Subpopulation

**Figure 16:**
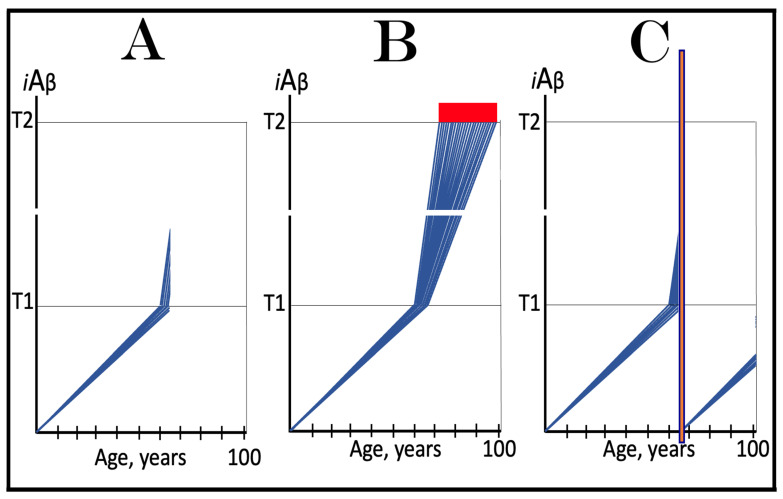
**Prevention of AD by transient depletion of iAβ via its targeted degradation in asymptomatic high-risk individuals.** *i*Aβ: Intraneuronal Aβ. *Blue lines*: Levels of *i*Aβ in individual neurons. ***T1*** *threshold*: The concentration of AβPP-derived *i*Aβ that mediates elicitation of the ISR and triggers the activation of the AβPP-independent *i*Aβ production pathway. ***T2*** *threshold*: The level of *i*Aβ, produced mainly in the AβPP-independent pathway, which triggers neuronal apoptosis. *Red box*: Apoptotic Zone, the range of *i*Aβ concentrations that cause the commitment to apoptosis. *Orange box*: The duration of the transient administration of an ACH2.0-based drug. Panel (**A**): The initial state of the levels of *i*Aβ in the neuronal population of an individual. A significant fraction of the neuronal population has already crossed the T1 threshold and activated the AβPP-independent *i*Aβ production pathway. Panel (**B**): The evolution of the initial state in the absence of the treatment. The initially sub-T1 neurons cross the T1 threshold; the AβPP-independent *i*Aβ generation pathway is now active in all affected neurons. The levels of *i*Aβ rapidly increase, cross the T2 threshold and trigger apoptosis. When a sufficient proportion of neurons lose their functionality or die, the disease enters the end stage. Panel (**C**): The evolution of the initial state following the *i*Aβ depletion treatment with an ACH2.0-based drug. *i*Aβ is depleted in all neurons, including the initial over-T1 neuronal fraction, and the operation of the AβPP-independent *i*Aβ production pathway in this neuronal subpopulation ceases. The de novo accumulation of *i*Aβ, produced at this point solely in the AβPP proteolytic pathway, resumes from the low baseline. Provided the depletion was sufficiently deep, the T1 threshold would not be reached and AD would not occur within the lifetime of the treated individual. Thus, the one-time-only transient administration of the ACH2.0-based drug can potentially provide lifelong protection from AD even in high-risk individuals. Note that this result is in sharp contrast to the outcome of the long-term employment of the ACH-based drugs in a similar setting, shown in Figure 6 above.

Figure 16 considers a situation where the transient *i*Aβ depletion treatment is administered to an asymptomatic high-risk individual. Whereas no AD symptoms have yet manifested, a fraction of the neuronal population has already crossed the T1 threshold and activated the AβPP-independent *i*Aβ production pathway. This initial state of the neuronal population is depicted in panel A of Figure 16. The evolution of this initial state in an untreated individual is shown in panel B of Figure 16. In addition to the initially over-T1 neuronal subpopulation, the remaining, initially sub-T1, affected neurons cross the T1 threshold in a relatively narrow temporal window [1,4]. The AβPP-independent *i*Aβ generation pathway is now active in all affected neurons. The levels of *i*Aβ rapidly increase, cross the T2 threshold, and trigger apoptosis. When a sufficient proportion of neurons lose their functionality or die, the disease enters the end stage. The evolution of the initial state following the transient *i*Aβ depletion treatment with an ACH2.0 drug is illustrated in panel C of Figure 16. *i*Aβ is depleted in all neurons, including the initial over-T1 neuronal fraction, and the operation of the AβPP-independent *i*Aβ production pathway in this neuronal subpopulation ceases. The de novo accumulation of *i*Aβ, produced at this point solely in the AβPP proteolytic pathway, resumes from the low baseline. Provided the depletion was sufficiently deep, the T1 threshold would not be reached and AD would not occur within the lifetime of the treated individual. Thus, the one-time-only transient administration of the ACH2.0-based drug can potentially provide lifelong protection from AD even in high-risk individuals. It should be emphasized that the ACH2.0-based drugs-mediated *i*Aβ depletion treatment would confer protection from symptomatic AD if administered any time prior to the manifestation of symptoms of the disease (rather than prior to the crossing of the T1 threshold, as is the case with the ACH-based drugs; compare with Figure 6 in Section 8.2 above).

## 20. Implementation of ACH2.0-Based Drugs in Prevention and Treatment of AACD

### 20.1. Transient iAβ Depletion Treatment in Prevention of AACD

**Figure 17:**
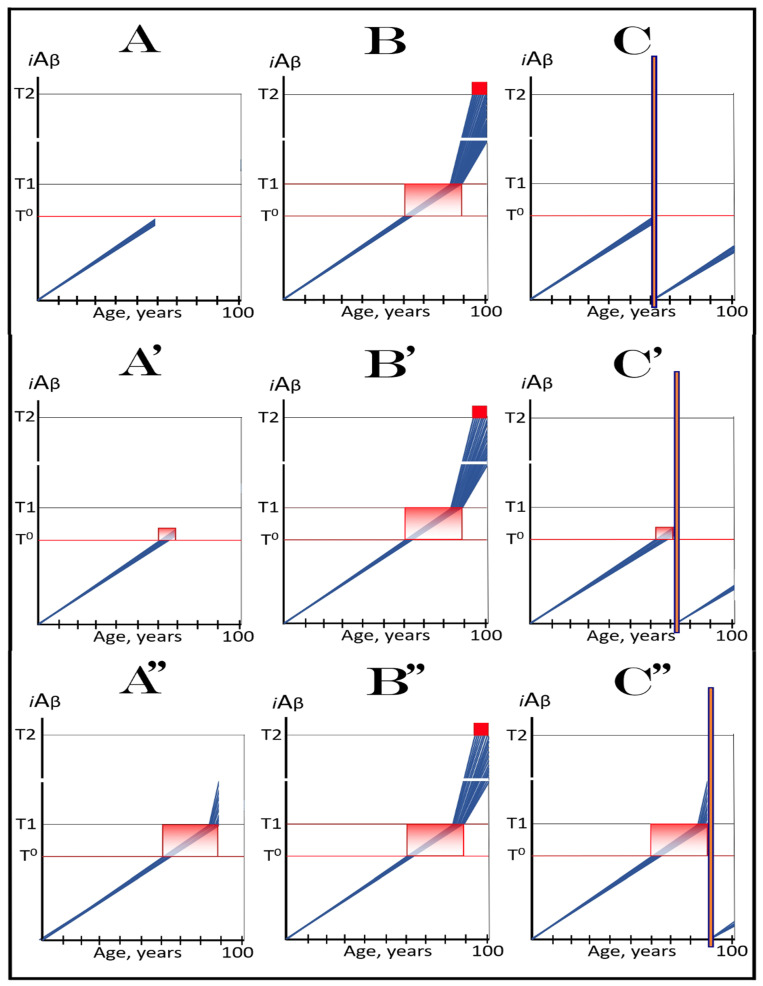
**ACH2.0-based drugs in prevention and treatment of AACD.*** i*Aβ: Intraneuronal Aβ. *Blue lines*: Levels of *i*Aβ in individual neurons. ***T*^0^** *threshold*: The concentration of *i*Aβ that triggers the neurodegeneration manifesting as AACD. ***T1*** *threshold*: The concentration of AβPP-derived *i*Aβ that mediates elicitation of the ISR and triggers the activation of the AβPP-independent *i*Aβ production pathway. ***T2*** *threshold*: The level of *i*Aβ, produced mainly in the AβPP-independent pathway, which triggers neuronal apoptosis. *Pink gradient boxes*: AACD Zone, the range of concentration of AβPP-derived *i*Aβ between the T^0^ and T1 boundaries. *Red boxes*: Apoptotic Zone, the range of *i*Aβ concentrations that cause the commitment to apoptosis. *Orange boxes*: The duration of the transient administration of an ACH2.0-based drug. Panels (**A**–**C**): Transient *i*Aβ depletion treatment in prevention of AACD. Panel (**A**): The initial state of the levels of AβPP-derived *i*Aβ in the neuronal population of an individual; they have not yet reached the T^0^ threshold, and no AACD symptoms have yet manifested. Panel (**B**): The evolution of the initial state in the untreated individual. The AβPP-derived *i*Aβ levels reach the T^0^ threshold and cross into the AACD Zone. AACD symptoms manifest and the condition progresses in parallel with the increase in the levels of AβPP-derived *i*Aβ. Provided a sufficient lifespan, the levels of AβPP-derived *i*Aβ would cross the T1 threshold, trigger the activation of the AβPP-independent *i*Aβ production pathway, and AD would ensue. Panel (**C**): The evolution of the initial state following the transient *i*Aβ depletion treatment with an ACH2.0-based drug. The *i*Aβ levels are substantially reset, and the de novo accumulation of AβPP-derived *i*Aβ resumes from a low baseline. Provided the *i*Aβ depletion is sufficiently deep, its levels would not reach the T^0^ threshold within the lifetime of the treated individual and no AACD (and, moreover, no AD) would occur. Panels (**A’**–**C’**): Transient *i*Aβ depletion treatment in early AACD. Panel (**A’**): The initial state of the *i*Aβ levels in the neuronal population of the patient; they all have crossed the T^0^ threshold and the early AACD symptoms have manifested; none has yet crossed the T1 threshold. Panel (**B’**): The evolution of the initial state in the untreated AACD patient; essentially, the same as in panel (**B**) above. Panel (**C’**): The effect of the transient *i*Aβ depletion treatment on the evolution of the initial state. The levels of *i*Aβ are substantially reduced; at this point, the patient is technically cured. The de novo accumulation of AβPP-derived *i*Aβ commences from a low baseline and the restoration of its levels would require decades. Neither the crossing of the T^0^ threshold would occur, nor AACD would recur within the lifetime of the treated patient. Panels (**A”**–**C”**): Transient *i*Aβ depletion treatment in late AACD. Panel (**A”**): the initial state of the *i*Aβ levels in the neuronal population of a late-stage AACD patient. In all neurons, the *i*Aβ levels have crossed the T^0^ threshold and progressed deep into the AACD Zone. The patient is asymptomatic for AD but in a subpopulation of his neurons, the levels of *i*Aβ have already crossed the T1 threshold, and the AβPP-independent *i*Aβ production pathway is operational. Panel (**B”**): The evolution of the initial state in the untreated patient. The initially sub-T1 neuronal fraction crosses the T1 threshold, the AβPP-independent *i*Aβ generation pathway becomes operational in all affected neurons, and the AD progresses toward the end stage. Panel (**C”**): The evolution of the initial state following the transient administration of an ACH2.0-based drug. *i*Aβ is substantially depleted, and its levels are reduced to nearly the initial baseline in *all* neurons, both sub-T1 and over-T1; the operation of the AβPP-independent *i*Aβ production pathway ceases. At this stage, the patient is cured, subject to the restoration of full functionality in the affected neurons. The de novo accumulation of *i*Aβ, produced now solely in the AβPP proteolytic pathway, resumes from the low baseline. The levels of AβPP-derived *i*Aβ would not reach the T^0^ threshold and neither AACD would recur nor AD would occur within the lifetime of the treated patient.

In terms of ACH2.0, preventing AACD requires precluding the levels of AβPP-derived *i*Aβ from reaching the T^0^ threshold. Panels A through C of Figure 17 depict the implementation of an ACH2.0-based drug in achieving this objective. Panel A shows the initial state of the levels of AβPP-derived *i*Aβ in the neuronal population of an individual. They have not yet reached the T^0^ threshold, and no AACD symptoms have manifested. Panel B of Figure 17 shows the evolution of the initial state in the untreated individual. The AβPP-derived *i*Aβ levels reach the T^0^ threshold and cross into the AACD Zone (defined above). AACD symptoms manifest and the condition progresses in parallel with the increase in the levels of AβPP-derived *i*Aβ. Provided the sufficient lifespan of the individual, the levels of AβPP-derived *i*Aβ would reach and cross the T1 threshold, trigger the activation of the AβPP-independent *i*Aβ production pathway, and AD would ensue. Panel C of Figure 17 illustrates the evolution of the initial state following the transient *i*Aβ depletion treatment with an ACH2.0-based drug. As a result of the treatment, the *i*Aβ levels are substantially reset, and the de novo accumulation of AβPP-derived *i*Aβ resumes from a low baseline. Provided the *i*Aβ depletion is sufficiently deep, its levels would not reach the T^0^ threshold within the lifetime of the treated individual and no AACD (and, moreover, no AD) would occur. It should be noted that since the age of the onset of AACD is statistically greater than that of AD (discussed above), the potentially once-in-a-lifetime AD-preventive transient iAb depletion treatment would also protect from AACD.

### 20.2. Transient iAβ Depletion Treatment in Early AACD

The ACH2.0-based drugs are potentially also capable of treating and curing AACD. Indeed, in the ACH2.0 framework, this condition is driven by AβPP-derived *i*Aβ within a certain range of concentrations (T^0^ to T1 thresholds; the AACD Zone). The successful treatment, therefore, requires the depletion of the *i*Aβ levels below the T^0^ level. If, following the *i*Aβ depletion treatment, the affected neurons are capable of restoring their functionality, the treated AACD patient would be cured. Panels A’ through C’ of Figure 17 show the implementation of the transient *i*Aβ depletion therapy in the treatment of early AACD. Panel A’ depicts the initial state of the *i*Aβ levels in the neuronal population of the patient. They all have crossed the T^0^ threshold and the early AACD symptoms have manifested; none have yet crossed the T1 threshold. Panel B’ of Figure 17 shows the evolution of the initial state in the untreated AACD patient. It is, essentially, the same as in panel B of the same figure: AβPP-derived *i*Aβ would further accumulate and the condition would progress. If and when (depending on the longevity of the patient), its levels cross the T1 threshold, the AβPP-independent *i*Aβ production pathway would be activated, and the AD would commence. Panel C’ of Figure 17 illustrates the effect of the transient *i*Aβ depletion treatment on the evolution of the initial state. The levels of *i*Aβ are substantially reduced; at this point, the patient is technically cured. The de novo accumulation of AβPP-derived *i*Aβ commences from a low baseline and, assuming it proceeds at the pre-treatment rate, the restoration of its levels would require decades. Consequently, neither the crossing of the T^0^ threshold would occur nor would AACD recur within the lifetime of the treated patient.

### 20.3. Transient iAβ Depletion Treatment in Late AACD

In terms of ACH2.0, AACD is driven by AβPP-derived *i*Aβ. Therefore, as discussed above, AACD can be potentially treated with ACH-based drugs. These drugs, however, are ineffective in the late AACD cases that are asymptomatic for AD but where a fraction of, and, possibly, the entire neuronal population have already crossed the T1 threshold (see Section 11 above) and where *i*Aβ is produced mainly in the AβPP-independent pathway, which is insensitive to ACH-based drugs. On the other hand, such a scenario presents no difficulties for ACH2.0-based drugs. Indeed, as illustrated in panels A” through C” of Figure 17, the transient *i*Aβ depletion therapy would be as effective in the late AACD cases as it presumably is in the early instances of this condition. Panel A” of Figure 17 depicts the initial state of the *i*Aβ levels in the neuronal population of a late-stage AACD patient. In all neurons, the *i*Aβ levels have crossed the T^0^ threshold and progressed deep into the AACD Zone. The patient is asymptomatic for AD but in a subpopulation of his neurons, the levels of *i*Aβ have already crossed the T1 threshold, and the AβPP-independent *i*Aβ production pathway is operational. Panel B” of Figure 17 shows the evolution of the initial state in the untreated patient. The initially over-T1 neuronal subpopulation continues to accumulate *i*Aβ, produced mainly in the AβPP-independent pathway. The initially sub-T1 neuronal fraction crosses the T1 threshold, the AβPP-independent *i*Aβ generation pathway becomes operational in all affected neurons, and the AD progresses toward the end stage. Panel C” of Figure 17 illustrates the evolution of the initial state following the transient administration of an ACH2.0-based drug. *i*Aβ is substantially depleted, and its levels are reduced to nearly the initial baseline in *all* neurons, both sub-T1 and over-T1. At this stage, the patient is cured, subject to the restoration of full functionality in the affected neurons. In the initially over-T1 neuronal subpopulation, the operation of the AβPP-independent *i*Aβ production pathway ceases. The de novo accumulation of *i*Aβ, produced now solely in the AβPP proteolytic pathway, resumes from the low baseline. The levels of AβPP-derived *i*Aβ would not reach the T^0^ threshold and neither AACD would recur nor would AD occur within the lifetime of the treated patient. In this situation, the remarkable efficiency of the ACH2.0-based drug is due to its ability to switch off the operation of the AβPP-independent *i*Aβ production pathway by interrupting the self-sustaining cycle and depriving the pathway of its stimulant. This ability of the ACH2.0-based drugs is at the core of its potential effect in symptomatic AD, discussed in the following section.

## 21. The Proverbial “Holy Grail” of the Alzheimer’s Research Attained: ACH2.0-Based Drugs Are Capable of Treatment of AD at Its Symptomatic Stages

Arguably, the prevention of AD is of the greatest strategic importance in addressing the disorder. Practically and emotionally, however, the ability to treat and possibly cure the disease at its devastating symptomatic stages has always been and remains the “Holy Grail” of Alzheimer’s research. No drug developed to date has even approached this goal. As discussed above, in terms of the ACH2.0, the implementation of the ACH-based drugs, although potentially useful in the prevention of AD, conceptually would be, and was, in fact, shown in multiple clinical trials to be futile at the symptomatic stages of Alzheimer’s disease. This is because, following the crossing of the T1 threshold and the commencement of AD, its pathology is driven by *i*Aβ generated mainly in the AβPP-independent pathway, which is completely insensitive to the ACH-based drugs. The key to the efficient treatment of AD at its symptomatic stages is, therefore, the reduction in the levels of *i*Aβ (i.e., the removal of the driver of the disease) and the cessation of its supply, i.e., the termination of the operation of the AβPP-independent *i*Aβ production pathway. The ACH2.0-based drugs fulfill both objectives: by achieving the former, they accomplish the latter. Indeed, conceptually, the ACH2.0-based drugs are the agents capable of the efficiently targeted degradation of *i*Aβ (see Section 26 below on the highly plausible prime candidates for this role). On the other hand, the operation of the AβPP-independent *i*Aβ production pathway requires, depends upon, and is propagated and perpetuated by sufficient levels of *i*Aβ; this is the essence of the AD Engine defined above. When the *i*Aβ levels are reduced to those below the T1 threshold, the operation of the AβPP-independent *i*Aβ generation pathway ceases and the AD Engine stops. Following its transient depletion, the accumulation of *i*Aβ resumes de novo but, at this point, its only source is the AβPP proteolysis. If the ACH2.0-based drug-mediated *i*Aβ depletion is “deep” enough, the single, once-in-a-lifetime transient treatment could be sufficient to preclude the AβPP-derived *i*Aβ levels from reaching the T1 threshold within the lifetime of the treated AD patient. In the absence of its driver, the progression of the disease would stop; the condition of the patient would at least stabilize and possibly improve, subject to the stage of intervention, the proportion of the remaining viable neurons, and their capacity for functional restoration.

**Figure 18:**
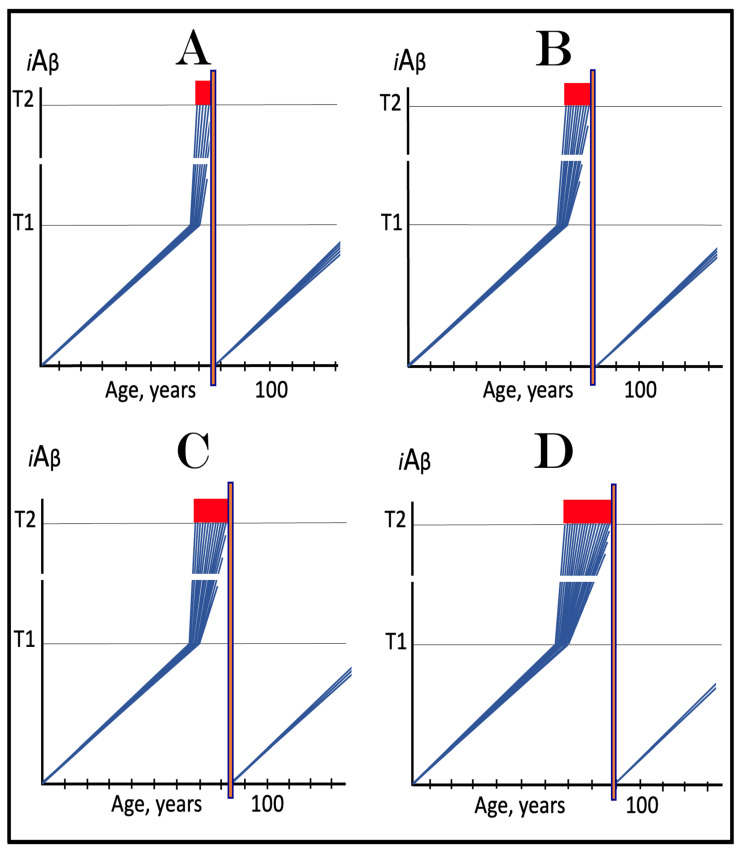
**ACH2.0-based drugs are capable of treatment of AD at its symptomatic stages.** *i*Aβ: Intraneuronal Aβ. *Blue lines*: Levels of *i*Aβ in individual neurons. ***T1*** *threshold*: The concentration of AβPP-derived *i*Aβ that mediates elicitation of the ISR and triggers the activation of the AβPP-independent *i*Aβ production pathway. ***T2*** *threshold*: The level of *i*Aβ, produced mainly in the AβPP-independent pathway, which triggers neuronal apoptosis. *Red boxes*: Apoptotic Zone, the range of *i*Aβ concentrations that cause the commitment to apoptosis. *Orange boxes*: The duration of the transient administration of an ACH2.0-based drug. Panel (**A**): The transient administration of the ACH2.0-based drug is implemented at the early stage of AD. At this time the majority of the affected neurons have not yet reached the T2 threshold and are still viable and capable of recovering and reconnecting. The treatment resets the levels of *i*Aβ to nearly their initial baseline. The operation of the AβPP-independent *i*Aβ production pathway ceases, and the *i*Aβ accumulation commences de novo from the low baseline and solely in the AβPP proteolytic pathway. Provided the post-treatment rate of accumulation of AβPP-derived *i*Aβ is similar to that occurring pre-treatment, the T1 threshold would not be reached and the disease would not recur within the remaining lifespan of the treated patient. Panels (**B**–**D**): The transient administration of the ACH2.0-based drug is executed at more and more advanced stages of the disease. The outcomes are principally identical to the one described above except that with the advancement of the disease, the progressively increasing proportions of the affected neurons cross the T2 threshold and commit apoptosis; this leaves fewer and fewer viable neurons capable of recovering their functionality. In this case, the progression of AD would stop but the recovery of cognitive functions would depend of the proportion of the redeemed neurons.

The above scenario is illustrated in Figure 18. The transient *i*Aβ depletion treatment is administered at the different stages of AD. It is assumed that the treatment completely, or nearly completely, depletes the levels of *i*Aβ and that the rate of AβPP-derived *i*Aβ accumulation is the same both pre- and post-treatment. In panel A of Figure 18, the transient *i*Aβ depletion treatment is administered at the early symptomatic stage. A fraction of the patient’s neurons have crossed both the T1 and T2 thresholds, initiated apoptosis, and triggered the manifestation of AD symptoms. This neuronal fraction is unredeemable. A substantial neuronal subpopulation, the majority at this early symptomatic stage, did not yet reach the T2 threshold. In these neurons, cellular damage is either insubstantial or reversible. The *i*Aβ depletion therapy resets the levels of *i*Aβ to a low baseline. The operation of the AβPP-independent *i*Aβ production pathway ceases. The accumulation of *i*Aβ resumes de novo from the initial or the nearly initial baseline, and is supported solely by its production in the AβPP proteolytic pathway. Its levels would not reach the T1 threshold and the disease would not re-ignite within the lifetime of the treated patient. The progression of the disease would be certainly stopped. The condition of the patient and their cognitive functions are expected to improve. Provided the still-viable neurons are capable of recovering their functionality and reconnecting, the patient would be at least partially cured.

Panels B through D of Figure 18 illustrate the effects of the administration of the transient *i*Aβ depletion treatment at various symptomatic stages of AD. Conceptually, the effect at the advanced stages of AD is the same as at its early stage, depicted in panel A. The difference is the proportion of remaining viable neurons at the time of the treatment administration. As the disease advances, progressively more affected neurons cross the T2 threshold and commit apoptosis; they are either dead or unresponsive to the treatment. This leaves a progressively smaller number of still-viable, treatment-responsive neurons. In these cells, following the reduction in the *i*Aβ levels, the operation of the AβPP-independent *i*Aβ generation pathway would stop. The accumulation of *i*Aβ would recommence de novo from the low baseline and only in the AβPP proteolytic pathway. Its levels would not reach the T1 threshold and the disease would not resume within the remaining lifespan of the treated patient. The treatment would certainly stop or decelerate the progression of the disease and the conditions of the treated patients are expected to improve but the restoration of the cognitive functions would be progressively less likely at more advanced stages of AD.

It should be emphasized that at the symptomatic stages of AD, the duration of the *i*Aβ depletion treatment depends on both the reduction in the levels of *i*Aβ to a sufficiently low baseline and the cessation of the operation of the AβPP-independent *i*Aβ production pathway. This is because, in these circumstances, the former does not directly and immediately trigger the latter. The *i*Aβ-driven AD pathology involves the generation of various stressors, such as substantial levels of unfolded and/or misfolded proteins. As a result, if the ISR alone is sufficient to maintain the activity of the AβPP-independent *i*Aβ generation pathway, then even after the depletion of *i*Aβ below the T1 threshold, these stressors may sustain the operation of the pathway until their levels are sufficiently reduced through the action of cellular chaperones. Consequently, if the *i*Aβ depletion therapy is terminated while the AβPP-independent *i*Aβ production pathway is still operational, *i*Aβ could rapidly accumulate to over-T1 levels and re-ignite the AD Engine.

## 22. Clinical Trials of ACH2.0-Based Drugs in Prevention of AD: Unambiguous Evaluation Even with High-Risk Over-T1 Participants

**Figure 19:**
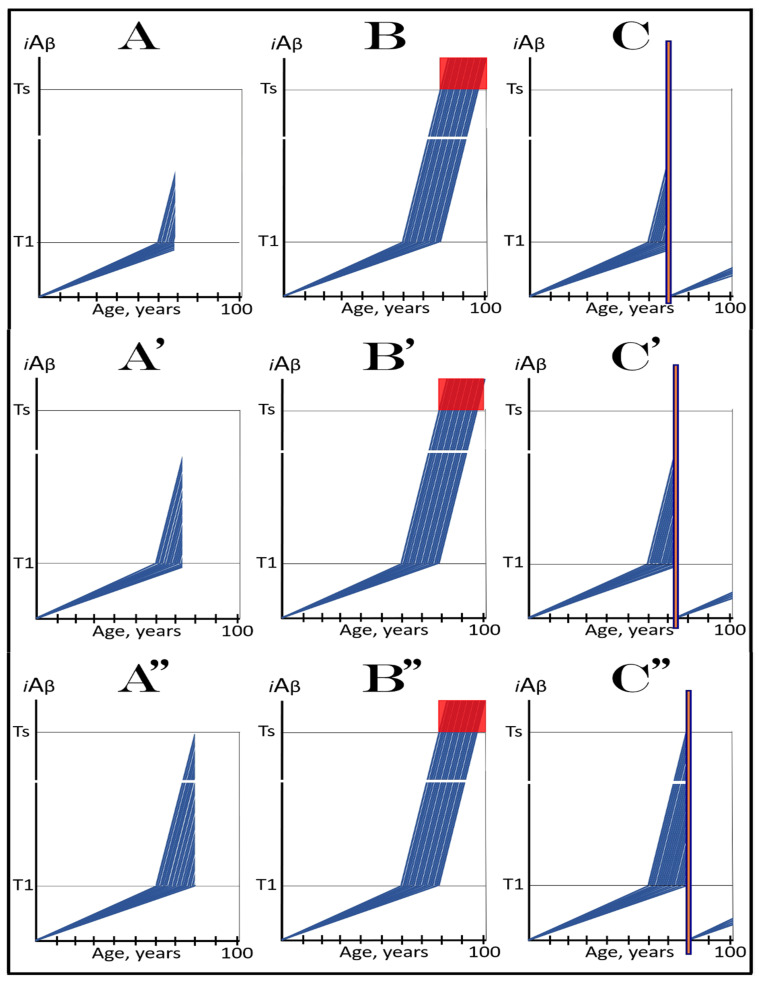
**Clinical trials of ACH2.0-based drugs in prevention of AD with high-risk cohorts.** *i*Aβ: Intraneuronal Aβ. *Blue lines*: The levels of *i*Aβ in individual trial subjects. ***T1*** *threshold*: The concentration of AβPP-derived *i*Aβ that mediates elicitation of the ISR and triggers the activation of the AβPP-independent *i*Aβ production pathway. ***Ts*** *threshold*: The levels of *i*Aβ that trigger the manifestation of AD symptoms. *Pink boxes*: Symptomatic Zone, the range of *i*Aβ concentrations above the Ts threshold. *Orange boxes*: The duration of administration of an ACH-based drug. Panel (**A**): All trial subjects are asymptomatic but about 50% have crossed the T1 threshold and activated the AβPP-independent *i*Aβ production pathway. Panel (**B**): The evolution of the initial stage in the placebo group. The levels of AβPP-derived *i*Aβ cross the T1 threshold and the AβPP-independent *i*Aβ generation pathway becomes operational in all trial subjects. *i*Aβ levels increase and cross the Ts threshold, subjects enter the Symptomatic Zone and AD symptoms manifest. Panel (**C**): The evolution of the initial state following the transient administration of an ACH2.0-based drug (orange box). The transient *i*Aβ depletion via its targeted degradation resets the levels of *i*Aβ to nearly its initial baseline in all trial subjects, both over-T1 and sub-T1. The operation of the AβPP-independent *i*Aβ production pathway ceases and the accumulation of *i*Aβ resumes de novo supported now only by its production in the AβPP proteolytic pathway. The levels of AβPP-derived *i*Aβ would not reach the T1 threshold and AD would not occur (for initially sub-T1 patients) or recur (for initially over-T1 patients) within the remaining lifetimes of the treated individuals. Panels (**A’**–**C’**,**A”**–**C”**) reiterate the same sequence as shown in panels A through C but with increasing proportions of asymptomatic over-T1 subjects: 75% in (**A’**–**C’**) and 100% in (**A”**–**C”**). In both cases, the evolutions of the initial states in the treated and the placebo cohorts are identical to those described above. In all three scenarios, the outcomes in the treated cohorts are unequivocally distinct from those in the placebo groups and are unambiguously interpretable.

The present and the two following sections analyze the evaluation of the efficiency of the ACH2.0-based drugs in clinical trials. These sections consider three situations where the assessment of the ACH-based drugs was shown problematic or impossible. They conclude that the evaluation of the efficacy of the ACH2.0-based drugs in the same circumstances would yield unequivocal conclusions. The present section examines clinical trials of ACH2.0-based drugs for the prevention of AD in high-risk individuals (compared with Section 15 above, which concluded that clinical trials of the ACH-based drugs in the same setting are practically impossible and/or self-defeating). Figure 19 presents three different situations identical to those considered in Figure 12 (Section 15 above) but with drastically distinct outcomes. In all three parts of Figure 19, all selected subjects are asymptomatic for AD but are at high risk for developing the disease. As discussed above, it is inevitable that a substantial proportion of trial participants or even the entire cohort are over T1, i.e., the levels of AβPP-derived *i*Aβ in their neurons have crossed the T1 threshold. Panel A in the upper part of Figure 19 depicts the initial state where about 50% of the trial participants are over T1. In these individuals, the AβPP-independent *i*Aβ production pathway is operational; its product (*i*Aβ generated independently of AβPP) is rapidly accumulating but has not yet reached the Ts threshold (“symptomatic threshold”), and the subjects remain asymptomatic. Panel B in the upper part of Figure 19 shows the evolution of the initial state in the untreated (placebo) cohort. The levels of AβPP-derived *i*Aβ in initially sub-T1 trial subjects cross the T1 threshold and trigger the activation of the AβPP-independent *i*Aβ production pathway. At this point, all subjects are over T1. The levels of *i*Aβ, produced mainly independently of AβPP, rapidly increase and eventually cross the Ts threshold. All subjects enter the Symptomatic Zone (pink box) and the AD symptoms manifest. Panel C in the upper part of Figure 19 illustrates the evolution of the initial state following the transient *i*Aβ depletion therapy (orange box). As a result of the treatment, the levels of *i*Aβ are substantially reduced in *all* subjects, and the operation of the AβPP-independent *i*Aβ generation pathway in over-T1 subjects ceases. The de novo accumulation of *i*Aβ, now produced solely in the AβPP proteolytic pathway, resumes from the low baselines in all treated participants. Its levels would not reach the T1 threshold within the lifetime of the treated subjects; the disease would be prevented in all subjects, there would be no overlap of any sort with the outcomes in the placebo cohort, and the outcomes of the trial would be unambiguously clear and lucidly interpretable.

In the middle part of Figure 19, panel A’ depicts the initial state where about 75% of the trial subjects are over T1 and produce *i*Aβ in the AβPP-independent pathway. The levels of *i*Aβ, however, have not yet reached the Ts threshold and these subjects remain asymptomatic for AD. The evolution of this initial state, shown in panel B’ of Figure 19, is similar to that depicted in panel B of the same Figure: the levels of *i*Aβ, produced mainly independently of AβPP, increase, reach the Ts threshold, cross into the Symptomatic Zone, and the AD symptoms manifest in all subjects. Panel C’ in the middle part of Figure 19 illustrates the evolution of the initial state following the implementation of the transient *i*Aβ depletion therapy. The levels of *i*Aβ substantially decreased in all trial participants. The AβPP-independent *i*Aβ production pathway is rendered inoperative, and Aβ, and consequently *i*Aβ, are produced only by the AβPP proteolysis. The accumulation of AβPP-derived *i*Aβ resumes de novo and its levels would not reach the T1 threshold within the remaining lifespans of treated trial participants. The disease is fully prevented in the medicated cohort, there is no overlap with the outcomes in the placebo group, and the conclusions of the trial are unequivocal.

Panel A” in the bottom part of Figure 19 shows the initial state where the levels of AβPP-derived *i*Aβ have crossed the T1 threshold and the AβPP-independent *i*Aβ production pathway has been activated in all trial subjects; they all are over T1, but remain asymptomatic. As a practical aspect, it should be emphasized that this is the most likely situation if only high-risk subjects are selected. Panel B” in the bottom part of Figure 19 depicts the evolution of this initial state. Conceptually, it is identical to those depicted in panels B and B’, only faster. The levels of *i*Aβ reach the Ts threshold, cross into the Symptomatic Zone, and the symptoms of AD manifest. In panel C” of Figure 19, the participants are subjected to transient *i*Aβ depletion therapy (orange box). This results in the levels of *i*Aβ being reduced to the initial or nearly initial baseline. The activity of the AβPP-independent *i*Aβ production pathway stops. The accumulation of *i*Aβ is resumed de novo and only in the AβPP proteolytic pathway. Its levels would not reach the T1 threshold within the lifetime of the treated subjects; the disease is prevented in all treated trial participants. This outcome is drastically distinct from results with the ACH-based drugs in the same situation, where all treated participants develop AD and the outcomes in the medicated and placebo cohorts are indistinguishable. With ACH2.0-based drugs, in sharp contrast, the outcomes of the trial are unambiguous.

## 23. Clinical Trials of ACH2.0-Based Drugs in Treatment of AACD: No Complications Even with Late-Stage Participants

**Figure 20:**
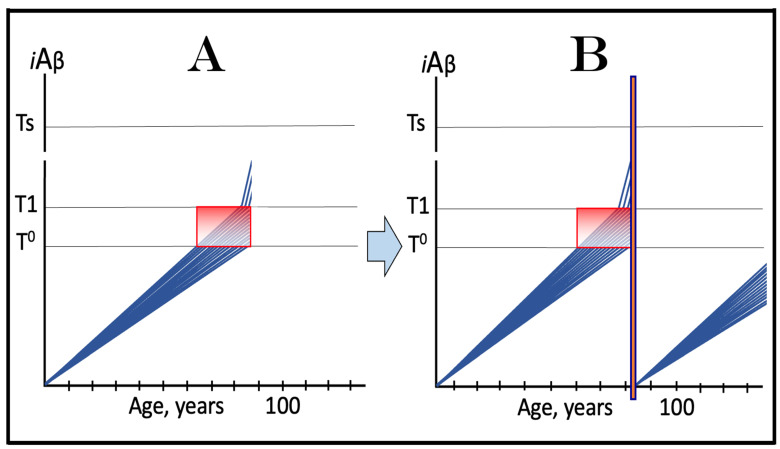
**Clinical trials of ACH2.0-based drugs in treatment of AACD are feasible even with late-stage patients.** *i*Aβ: Intraneuronal Aβ. *Blue lines*: The levels of *i*Aβ in individual trial subjects. ***T^0^*** *threshold*: The concentration of *i*Aβ that triggers the neurodegeneration manifesting as AACD. ***T1*** *threshold*: The concentration of AβPP-derived *i*Aβ that mediates elicitation of the ISR and triggers the activation of the AβPP-independent *i*Aβ production pathway. ***Ts*** *threshold*: The levels of *i*Aβ that trigger the manifestation of AD symptoms. *Pink gradient boxes*: AACD Zone, the range of concentration of AβPP-derived *i*Aβ between the T^0^ and T1 boundaries. *Orange box*: The duration of administration of an ACH-based drug. Panel (**A**): The initial state. All subjects exhibit the AACD symptoms and are asymptomatic for AD. In the majority of trial participants, the levels of *i*Aβ are confined between the boundaries of the T^0^ and T1 thresholds. In a fraction of trial subjects the levels of *i*Aβ have crossed the T1 threshold and triggered the activation of the AβPP-independent *i*Aβ production pathway. The evolution of this initial state in the placebo cohort is depicted in panel B of Figure 13 and is not shown here (briefly, all subjects cross the T1 threshold and AD ensues). Panel (**B**): The evolution of the initial state following the *i*Aβ depletion via transient administration of the ACH2.0-based drug (orange box). The transient *i*Aβ depletion via its targeted degradation resets the levels of *i*Aβ to nearly its initial baseline in all trial subjects, both over-T1 and sub-T1. The operation of the AβPP-independent *i*Aβ production pathway ceases. At this point, the treated trial participants are cured. The accumulation of *i*Aβ resumes de novo supported now only by its production in the AβPP proteolytic pathway. The levels of AβPP-derived *i*Aβ would not reach the T^0^ threshold and AACD would not recur within the remaining lifetimes of the treated individuals. The outcome in the treated cohort is distinctly different from that in the placebo group and can be unambiguously interpreted.

Above, a setting for the AD-preventive clinical trials was examined that is self-defeating for the ACH-based drugs but informatively straightforward for the ACH2.0-based drugs. Another conceptually similar example is the evaluation of a drug’s efficiency in the treatment of AACD in a setting that includes late-stage AACD patients. The analysis presented in Section 16 above concluded that such an assessment would be unfeasible for ACH-based drugs. The present section examines the performance of the ACH2.0-based drugs in an identical setting and establishes that it would result in unequivocal conclusions. The situation under discussion is illustrated in Figure 20. Panel A of Figure 20 depicts the initial state of clinical trials for the treatment of AACD. The only criteria for participation in this trial are the presentation of the AACD symptoms. In all selected participants the levels of AβPP-derived *i*Aβ have crossed the T^0^ threshold and entered the AACD Zone (pink gradient box); all participants are asymptomatic for AD. In a fraction of participating late-stage AACD patients, however, AβPP-derived *i*Aβ has crossed the T1 threshold and triggered the activation of the AβPP-independent *i*Aβ production pathway, yet these participants remain asymptomatic for AD. The evolution of this initial state in the untreated trial subjects is not shown here since it is presented in panel B of Figure 13 above. Briefly, AACD would progress, the levels of AβPP-derived *i*Aβ would eventually cross the T1 threshold in all initially sub-T1 subjects, and, provided sufficiently long lifespans, AD would ensue. The evolution of the initial state following the transient *i*Aβ depletion therapy (orange box) is shown in panel B of Figure 20. As a result of the treatment, the levels of *i*Aβ are substantially reduced in all patients, including the initially over-T1 fraction. In this fraction, the operation of the AβPP-independent *i*Aβ production pathway ceases. All treated trial subjects are technically cured. The de novo accumulation of *i*Aβ resumes solely in the AβPP proteolytic pathway. Its levels would not reach the T^0^ threshold and AACD would not recur within the lifetime of the treated trial subjects. The outcomes in the medicated cohort would not overlap in any way with those in the placebo group and thus would be unambiguously interpretable.

## 24. Clinical Trials of ACH2.0-Based Drugs in Treatment of Symptomatic AD: Lucidly Interpretable Outcomes at All Stages of the Disease

**Figure 21:**
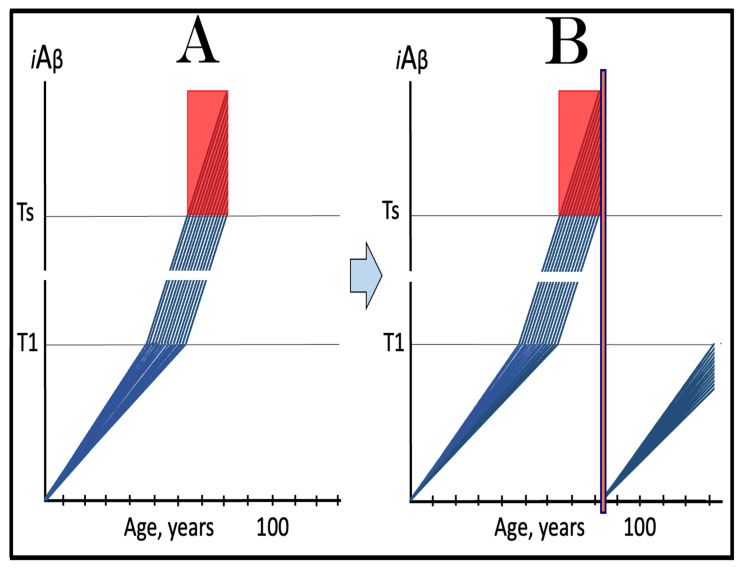
**Clinical trials of ACH2.0-based drugs in treatment of symptomatic AD.** *i*Aβ: Intraneuronal Aβ. *Blue lines*: The levels of *i*Aβ in individual trial subjects. ***T1*** *threshold*: The concentration of AβPP-derived *i*Aβ that mediates elicitation of the ISR and triggers the activation of the AβPP-independent *i*Aβ production pathway. ***Ts*** *threshold*: The levels of *i*Aβ that trigger the manifestation of AD symptoms. *Pink boxes*: Symptomatic Zone, the range of *i*Aβ concentrations above the Ts threshold. *Orange box*: The duration of the transient administration of an ACH2.0-based drug. Panel (**A**): The initial state of *i*Aβ levels in individual trial subjects at the commencement of the treatment. All trial participants are symptomatic for AD; this is the only criterion for their inclusion. The levels of *i*Aβ, produced mainly in the AβPP-independent pathway, have crossed the Ts threshold and entered the Symptomatic Zone (pink box) in all trial subjects. The cohort covers the entire spectrum of AD pathology: in different subjects, the symptoms vary from very early and mild to very advanced and severe. The evolution of this initial state in the placebo group is obvious: in all subjects, the disease progresses toward the end stage. Panel (**B**): Evolution of the initial state following the *i*Aβ depletion treatment. The levels of *i*Aβ are reset and substantially reduced in all trial subjects. The operation of the AβPP-independent pathway of *i*Aβ production ceases and it is now generated solely in the AβPP proteolytic pathway. Due to the insufficient levels of its principal driver, the progression of AD is arrested. The accumulation of the *i*Aβ initiates de novo, supported only by the AβPP proteolysis. The AβPP-derived *i*Aβ levels would not reach the T1 threshold and the disease would not resume within the lifetimes of the treated patients, the outcome that is unequivocally distinguishable from that occurring in the placebo cohort.

The final example of AD clinical trials, impossible with the ACH-based drugs but straightforward and informative with the ACH2.0-based drugs, is their evaluation in the treatment of symptomatic AD. Such trials are impossible with the ACH-based drugs because these drugs would have at best only marginal effect in very early AD (when a fraction of the neurons may not yet have crossed the T1 threshold), as exemplified by the clinical trials of lecanemab and donanemab, and would yield no beneficial effect whatsoever in the advanced stages of the disease when the AβPP-independent *i*Aβ production pathway has been activated in all affected neurons. In contrast, as discussed in Section 19 above, ACH2.0-based drugs would be effective in stopping the progression of the disease regardless of its stage. The design and the outcomes of the clinical trial examining the effect of ACH2.0-based drugs in symptomatic AD are considered in Figure 21. Panel A of Figure 21 depicts the initial state of the trial. All trial participants are symptomatic for AD; this is the only criterion for their inclusion. The levels of *i*Aβ, produced mainly in the AβPP-independent pathway, have crossed the Ts threshold and entered the Symptomatic Zone (pink box) in all trial subjects. The cohort covers the entire spectrum of AD pathology: in different subjects, the symptoms vary from very early and mild to very advanced and severe.

The evolution of the initial state in untreated trial subjects is obvious: the symptoms develop further, and the disease progresses and reaches the end stage in all untreated participants. The evolution of the initial state following the *i*Aβ depletion treatment is shown in panel B of Figure 21. The levels of *i*Aβ are reset and substantially reduced in all trial subjects. The operation of the AβPP-independent pathway of *i*Aβ production ceases and it is now generated solely in the AβPP proteolytic pathway. Due to the insufficient levels of its principal driver, the progression of AD is arrested. The accumulation of the *i*Aβ initiates de novo, supported only by the AβPP proteolysis. The AβPP-derived *i*Aβ levels would not reach the T1 threshold and the disease would not resume within the lifetimes of the treated patients. The prognostic outcomes would differ individually depending on the initial symptomatic stage of a patient. At the early stages, the majority of the neuronal population of an individual trial participant remains viable at the time of the treatment and is redeemed. It could be expected that the affected yet still-viable neurons would recover their functionality and reconnect; the cognitive functions would improve and the patient could be cured, at least partially. At more advanced stages of the disease, fewer neurons remain viable and thus redeemable by the treatment. The progression of the disease would cease or decelerate at every stage, however advanced, but the cognitive recovery would be increasingly less certain.

## 25. The Uniform Mechanistic Effect of the ACH2.0-Based Drugs Elicits Distinctly Stage-Specific Outcomes

The preceding sections establish that, if effective in the depletion of *i*Aβ via its targeted degradation, the ACH2.0-based drugs are therapeutically multi-potential. They are capable of the prevention of AACD and AD, they are efficient in curing AACD, and they are effective in the treatment of AD at its symptomatic stages. Such versatility of the ACH2.0-based drugs may create an impression of their mechanistic variability, i.e., that their mechanism of action is different in different circumstances. This impression, however, would be erroneous. The ACH-based drugs act in one way only: they actively deplete or mediate the direct “active” (as defined above) depletion of *i*Aβ. What differs distinctly and is the disease stage-specific, are the effects (rather than mechanisms of action) of the ACH2.0-based drugs and, accordingly, the outcomes of their deployment in different settings. In every case, the transient utilization of the ACH2.0 drugs would deplete *i*Aβ, force the reduction in its levels to the nearly initial baseline, and necessitate its de novo decades-long accumulation. When the levels of AβPP-derived *i*Aβ have not yet reached the T1 or T^0^ thresholds, this would preclude the T1 and/or T^0^ crossing, and thus prevent AACD and/or AD. At symptomatic stages of AACD and AD, the action of the ACH2.0-based drugs would be precisely the same, namely the reduction in the levels of *i*Aβ and the enforcement of its de novo accumulation from a low baseline. In these settings, however, the outcomes would be not the prevention but the treatment and, potentially, the cure of AACD or AD. Despite the dissimilarity of the outcomes, the underlying mechanism is the same: when a condition is treated, the driver of this condition is eliminated; when a condition is prevented, its potential driver is not allowed to come into play.

## 26. Activators of Physiologically Occurring Intra-*i*Aβ-Cleaving Capabilities of BACE1 and/or BACE2: Highly Plausible ACH2.0-Based Drugs for Prevention and Treatment of AD and AACD

The ACH2.0-based drugs were defined above as the agents that enact or mediate the targeted degradation of *i*Aβ, thus causing its “active”, forced depletion. This definition would include, for example, a small molecule capable of crossing into the brain, penetrating the neurons, and catalyzing the targeted, i.e., specific, degradation of *i*Aβ. This, however, is easier said than done. On the other hand, the enzymatic activities capable of performing precisely this, the targeted degradation of *i*Aβ by its intra-molecular cleavage, are encoded in humans and expressed in the neuronal cells and could potentially be manipulated therapeutically. There are at least two such activities and, incidentally, they are either the major or the ancillary components of the two familiar players in the Alzheimer’s play script: BACE1 and BACE2. Moreover, one of these activities confers protection from both AD and AACD when it is enhanced, whereas another activity operates in a physiologically protective capacity and its weakening causes the early onset of AD. In fact, these two activities are best known for their pivotal role in the functional outcomes of the two prominent Aβ mutations: “Icelandic” and “Flemish”.

The Icelandic mutation, which substitutes an “A” in position 2 of Aβ with a “T”, confers upon its carriers protection from both AD and AACD [21,22]. It does so by enhancing the efficiency of the BACE1-mediated cleavage at the alternative site within *i*Aβ. The primary cleavage site of BACE1 is known as the β-site. The alternative cleavage site, located ten residues downstream within *i*Aβ, is designated the β’-site. Cleavage at the β’-site of *i*Aβ degrades the molecule and thus depletes its levels. The augmentation of the β’-site cleavage is not limited to the carriers of the Icelandic mutation. It can be accomplished, apparently, by any increase in the activity of BACE1, for example, via its exogenous overexpression [139,140,141,142]. Indeed, the exogenous overexpression of human BACE1 in mouse models greatly elevated the rate of cleavage at the β’-site, increased the ratio of the N-truncated to the full-size Aβ, and significantly decreased the deposition of Aβ in mouse brain [140,141]. The β’-site is not the only alternative BACE1 cleavage site within the *i*Aβ molecule. As was shown in multiple investigations, BACE1 or BACE1-associated activity is also capable of cleaving between residues 34 and 35 of human iAβ [143,144,145,146]. Moreover, the exogenous overexpression of human BACE1 was demonstrated to substantially increase the rate of the *i*Aβ cleavage at residues 34/35 thus producing an intermediate in the iAβ clearance process [145].

As for the Flemish mutation, which causes the early onset of AD (FAD), the occurrence of this phenomenon is indicative of the protective physiological function of the BACE1 analog, BACE2. In contrast to BACE1 operation, where the major cleavage activity occurs at the β-site (thus generating Aβ), the main cleavage activity of BACE2 is at the two positions within *i*Aβ, at its residues 19 and 20, both phenylalanines [147]. Apparently, the physiological function of BACE2 is a protective one: to control and, if necessary, to limit the production and the levels of *i*Aβ [148]. Indeed, the inhibition of BACE2 in model systems resulted in a substantial elevation in the production of Aβ [62,148]. Moreover, the recent results obtained with the human pluripotent stem cell-derived brain organoids indicated that BACE2 could protect neuronal cells from Aβ-mediated apoptosis, whereas the deficiency of BACE2 activity may represent a common pathological mechanism not only for AD but also for Hirschsprung disease [149]. The notion that BACE2 is a physiologically occurring protective agent is strongly supported by the effect of Flemish mutation. This mutation occurs at the residue 21 of Aβ, contiguously downstream from the BACE2 cleavage sites. It substantially reduces the main cleavage activity of BACE2 and, consequently, leads to the elevation of the levels of *i*Aβ [62]. This, in turn, results in the early onset of AD.

It appears, therefore, highly plausible that a sufficient increase, potentially mediated by small-molecule activators, of the main activity of BACE2 or of the ancillary *i*Aβ-cleaving activities of BACE1 would be capable of the “active”, forced depletion of *i*Aβ either in the preventive or in the curative settings discussed in the preceding sections above. The transient activation of either BACE2 alone or of only the intra-*i*Aβ-cleaving activities of BACE1 could be sufficient to achieve a substantial, therapeutically meaningful depletion of *i*Aβ and the reduction in its levels. On the other hand, the concerted activation of the intra-*i*Aβ-cleaving capabilities of both, BACE1 and BACE2 would greatly synergize the process because these activities target different sites within *i*Aβ and, in addition, operate in different sub-cellular locations [150]. If, however, only one activity could be feasibly manipulated at a time, BACE2 appears the natural choice because the enhancement of its activity would constitute the augmentation of its physiological protective function. On the other hand, since the intra-*i*Aβ cleavage is the main activity of BACE2, it could be, evolutionary, already optimized physiologically and its efficiency may be hard to improve upon. In contrast, the intra-*i*Aβ-cleaving capabilities of BACE1 are its secondary, minor activities and, as such, could possibly be more amenable to optimization and enhancement, as exemplified by the effect of the Icelandic mutation.

It should be strongly emphasized that the activation of the intra-*i*Aβ-cleaving activities of BACE1 and/or BACE2 is the first choice in terms of therapeutic strategy for prevention and treatment of AD and AACD only because it is both feasible and plausible. Importantly, however, *any* suitable agent capable of a substantial depletion of *i*Aβ via its direct or mediated targeted degradation would constitute a potential ACH2.0-based drug.

## 27. Conclusions

The present study introduces the Amyloid Cascade Hypothesis 2.0, ACH2.0, a novel theory of Alzheimer’s disease and of Aging-Associated Cognitive Decline, and describes the class of potentially highly effective drugs suggested by this theory; this class is designated as the ACH2.0-based drugs. The study offers a comparative evaluation of the therapeutic potentials of the ACH2.0-based drugs versus those of the drugs whose design was informed by the Amyloid Cascade Hypothesis, referred to here as the ACH-based drugs. This analysis concludes that the utility of the ACH-based drugs is severely limited. Their short-term employment is ineffective, their long-term engagement is highly problematic, their implementation at the symptomatic stages of AD is futile, and their assessment in conventional clinical trials for prevention of AD is at best impractical, at worst impossible, and could be deceitful in between. In sharp contrast, the ACH2.0-based drugs have none of those shortcomings and are capable of delivering the proverbial “Holy Grail” of Alzheimer’s research: the ability to treat and potentially cure the disease at its symptomatic stages. The ACH2.0-based drugs would also be effective in the prevention of AD and AACD and in the treatment of the latter. Remarkably, the *modus operandi* of the ACH2.0-based drugs opens up the possibility of once-in-a-lifetime only, transient, short-duration therapy for the treatment and prevention of both AD and AACD.

The ACH2.0-based drugs act by substantially reducing the levels of *i*Aβ and necessitating its de novo accumulation from a low baseline. This may not be sufficient in cases where the ISR in neuronal cells is elicited by means other than AβPP-derived *i*Aβ-mediated activation of eIF2α kinases, such as in individuals predisposed to AD due to the persistent neuronal inflammation (resulting, for example, from repeated concussions). In these individuals, as discussed above, either the T1 threshold is lowered due to the increased basal level of cellular (neuronal) stress or the ISR is elicited and, subsequently, the AβPP-independent *i*Aβ production pathway is activated as the consequence of the inflammatory processes-associated stressors in neuronal cells even if the levels of AβPP-derived *i*Aβ do not reach the T1 threshold. In the latter cases, the anti-inflammatory treatment would act preventively prior to the onset of AD (i.e., prior to the activation of the AβPP-independent *i*Aβ production pathway), whereas, following its commencement, the transient implementation of the ACH2.0-based drugs should be combined with the long-term anti-inflammatory therapy.

In the framework of the ACH2.0, potentially, as discussed in [4], AD could be prevented, or its progression could be ceased via long-term inhibition of the ISR, for example with the small molecule ISR inhibitor ISRIB; this would preclude or stop the operation of the AβPP-independent *i*Aβ generation pathway and, consequently, of the AD Engine. This, apparently, is not a viable option, since, due to the central role of the integrated stress response in cellular physiology, its long-term suppression is highly likely to result in significant adverse effects.

In addition, the present study offers a solution for the conundrum of the spectacular efficiency of the ACH-based drugs in animal models versus their complete inefficacy in human clinical trials for symptomatic AD. Lastly, it posits that the prime candidates for the efficient ACH2.0-based drugs are the activators of the intra-*i*Aβ-cleaving capabilities of BACE1 and BACE2.
